# Design and synthesis of novel HDAC6 inhibitor dimer as HDAC6 degrader for cancer treatment by palladium catalysed dimerisation

**DOI:** 10.1080/14756366.2025.2468355

**Published:** 2025-02-27

**Authors:** Ching Lin, Jui-Ling Hsu, Yu-Tung Hsu, Kuo-Chen Fan, Sian-Siou Wu, Miao-Hsia Lin, Jih-Hwa Guh, Chao-Wu Yu

**Affiliations:** a School of Pharmacy, National Taiwan University, Taipei, ROC; b Department of Nursing, Chang Gung University of Science and Technology, Taoyuan City, ROC; c Division of Hematology-Oncology, Department of Internal Medicine, Chang Gung Memorial Hospital, Linkou Medical Center, Taoyuan City, ROC; d Department and Graduate Institute of Medical Microbiology, College of Medicine, National Taiwan University, Taipei, ROC

**Keywords:** HDAC6, protein degrader, anticancer, palladium, coupling

## Abstract

The enigmatic histone deacetylase 6 (HDAC6) is one of a kind among its family. Recent reports revealed that HDAC6 CD1 exhibits E3 ligase activity. Inspired by these researches, we attempted to develop drugs targeting HDAC6 *via* novel mechanism. Herein, we report a palladium catalysed transformation and purification method for hydroxamic acid dimers, and series of HDAC6 inhibitor-based dimer showing outstanding biological activities and capability of inducing auto-degradation. Our proof-of-concept was highlighted with 2-amino benzamide-based HDAC6 inhibitor dimers that exhibit great HDAC6 inhibition activity (3.9–15.4 nM), good HDAC1/6 selectivity (95–577), and excellent cytotoxicity against human hormone-resistant prostate cancer (HRPC) PC-3 and non-small cell lung cancer (NSCLC) A549 cell lines (5.9–11.3 and 6.6–17.9 μM, respectively) while simultaneously inducing HDAC6 degradation. These dimers not only induce apoptosis and autophagy but also interfere with kinetochore attachment by the detection of BUBR1 phosphorylation at S670.

## GRAPHICAL ABSTRACT

**Figure:**
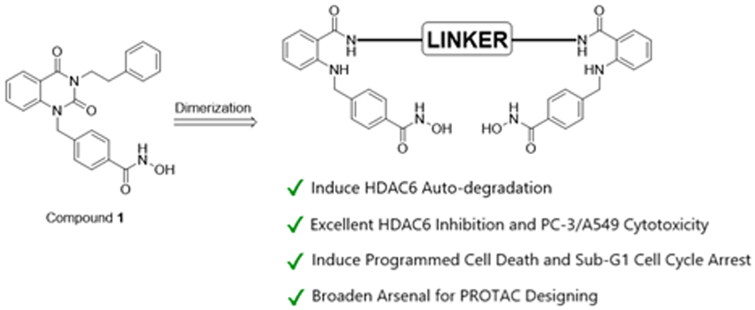

## Introduction

Histone deacetylases (HDACs) are a class of epigenetic regulatory enzymes that mediate gene expression through the deacetylation of histones. By catalysing the removal of acetyl groups, HDACs facilitate the compaction of chromatin, leading to the repressing gene transcription^1^. Currently, HDACs can be categorised into class I (HDAC1, 2, 3, 8), class II (HDAC4, 5, 6, 7, 9, 10), class III (sirtuins), and class IV (HDAC11), based on their sequence homology to yeast^2^. Class II enzymes can be further subdivided into class IIa (HDAC4, 5, 7, 9) and IIb (HDAC6, 10) according to their domain compositions. Notably, HDAC6 is known for its ability to deacetylate non-histone proteins, such as α-tubulin, cortactin, heat shock protein (Hsp90), etc^3^. Despite still being poorly understood, HDAC6’s diverse regulatory mechanisms and involvement in various diseases make it an attractive target for novel therapeutic interventions^4^.

Structurally, HDAC6 is unique among other subtypes in the HDAC family, possessing an extra zinc-finger ubiquitin binding domain (ZnF-UBD) and two catalytic domains (CDs) instead of one, with the one located near the N-terminus termed CD1 and the other one termed CD2^5^. Despite the two CDs of HDAC6 are highly conserved when compared to other class II HDACs, only CD2 exhibits full deacetylase activity while CD1 exhibiting compromised deacetylase activity due to a L353K mutation^6^^,^^7^. The actual function of HDAC6 CD1 and how it evolves remain unknown. Nonetheless, recently it has been reported that HDAC6 CD1 might functioned as a ubiquitin E3 ligase^8^^,^^9^. *In vitro* studies suggested that HDAC6 ubiquitinates MutS protein homolog 2 (MSH2) and checkpoint kinase 1 (Chk1), targeting them for degradation through the ubiquitin-proteasome system (UPS) pathway.

Previously, a series of HDAC6 proteolysis targeting chimaera (PROTAC) degraders have been reported. These PROTAC molecules consist of an E3 ligase ligand conjugated to an HDAC6 ligand *via* a rationally designed linker. Notably, most reported HDAC6-targeting PROTACs were designed based on the cereblon (CRBN) E3 ligase ligand^10–15^, with only one study developed Von Hippel-Lindau (VHL)-based degrader^16^. These bifunctional molecules could induce the degradation of HDAC6, demonstrating potential for the treatment of various cancers and inflammatory disorders. However, to date, none of the studies have utilised the ubiquitination activity of HDAC6 itself, which could play an important role in the UPS. These researches have inspired us to make use of this property to fill in gaps in designing PROTACs, understanding the role of HDAC6 in cellular events, and developing drugs towards diseases.

**Figure 1.**
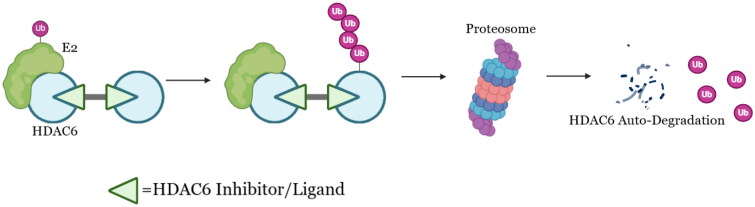
Working hypothesis of HDAC6 degrading dimers.

We report herein a series of HDAC6 degraders based on a selective HDAC6 inhibitor (**1**) previously developed by our group^17^. A palladium-catalysed dimerisation method under mild condition for generating HDAC6 ligand dimers was also reported in this article. The design of these HDAC6 degrading dimers stemmed from the auto-degradation phenomenon of homo-PROTACs^18–20^. The hypothetic working mechanism of the dimers is depicted in Figure 1. We successfully proven HDAC6 indeed functioned as ubiquitin E3 ligase. This result is attractive as it unlocks a whole new arsenal for functionable PROTAC designing. Moreover, our group has recently demonstrated HDAC6 inhibition as an effective treatment against TGF-β-induced idiopathic pulmonary fibrosis, we believe taking advantage of this new property of HDAC6 will boost our strategy towards treating the disease^21^. Last but not least, these HDAC6 degraders are potent against lung and prostate cancer cell lines with potentially novel mechanisms.

## Results and discussion

### Chemistry

**Scheme 1.**
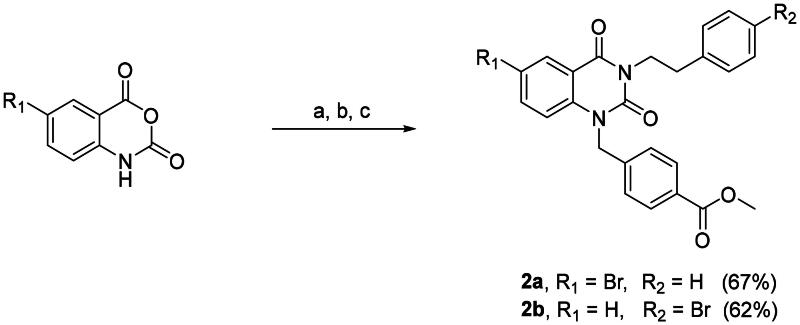
Synthesis of compounds **2a** and **2b**. Reagents and conditions: (a) Phenethylamine, EtOAc, rt, 1 h; (b) CDI, EtOAc, rt, 19 h; (c) K_2_CO_3_, methyl 4-(bromomethyl)benzoate, acetone, 60 °C, 3 h.

Dimers **4a–c** and **6a–c** are based on a potent selective HDAC6 inhibitor-bearing quinazolin-2,4-dione scaffold from our in-house library. Bromine atom was integrated into **1** as conjugation site following reported method (Scheme 1).

In the next step, halide substituted **2a** and **2b** were dimerised *via* Buchwald–Hartwig amination^22^^,^^23^. The reaction conditions were studied using the synthesis of **3b** from **2a** as model reaction, shown in Table 1. At the outset, the condition from the literature was applied (Table 1, entry 1); however, only trace amounts of the product were observed^24^. Then, a bulkier ligand was tested (Table 1, entry 2) and subjected to microwave irradiation expecting for faster reaction rate. However, only traces of the product were observed as well. We surmised that using a milder base and lower reaction temperature would better tolerate the labile ester group (Table 1, entry 3), which successfully improved the yield. We then investigated the effect of different ligands (Table 1, entries 4 and 5), revealing that BrettPhos is the optimal ligand. Table 1, entries 6–9 examined the effects of temperature and base. As we surmised, the ester group does not tolerate strong base such as NaO*t*-Bu, milder inorganic bases served as a better choice. Temperature is also crucial in this transformation. A threshold temperature of approximately 70 °C was observed for the reaction, as limited product was isolated at 60 °C (Table 1, entry 6), slightly higher yield under refluxing THF (Table 1, entry 3), and dramatically improved yield at 100 °C (Table 1, entry 8). Pushing the temperature slightly higher in refluxing dioxane did not further improve the yield (Table 1, entry 10).

**Table 1.** Condition optimisation for Buchwald–Hartwig amination^a^.

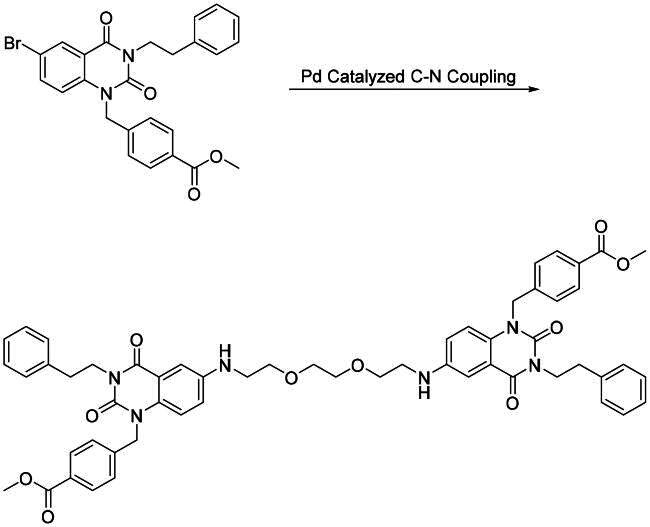					

Entry	Solvent	Catalyst	Base	Heating condition	Yield (%)^d^
1	Dioxane	BrettPhos G3 Precatalyst^c^	NaO*t*-Bu	80 °C, 22 h	Trace
2	Dioxane	AdBippyPhos G3 Precatalyst	NaO*t*-Bu	MW, 80 °C, 1 h	Trace
3	THF	BrettPhos G3 Precatalyst	Cs_2_CO_3_	Reflux, 24 h	13
4	THF	PEPPSI-iPent	Cs_2_CO_3_	Reflux, 24 h	Trace
5	THF	DPPF G3 Precatalyst	Cs_2_CO_3_	Reflux, 24 h	Trace
6	THF	BrettPhos G3 Precatalyst	Cs_2_CO_3_	60 °C, 24 h	Trace
7	THF	BrettPhos G3 Precatalyst	NaO*t*-Bu	60 °C, 24 h	Trace
8	Dioxane	BrettPhos G3 Precatalyst	Cs_2_CO_3_	100 °C, 24 h	69
9	Dioxane	BrettPhos G3 Precatalyst	NaO*t*-Bu	100 °C, 24 h	Trace
10^b^	Dioxane	BrettPhos G3 Precatalyst	Cs_2_CO_3_	Reflux, 24 h	69

^a^Reactions were performed on a 200 mg scale. 5 mol% catalyst, 3 equiv. base, 0.6 equiv. diamine, solvent (0.1 M). ^b^Gram scale. ^c^1 mol%. ^d^Isolated yield.

**Scheme 2.**
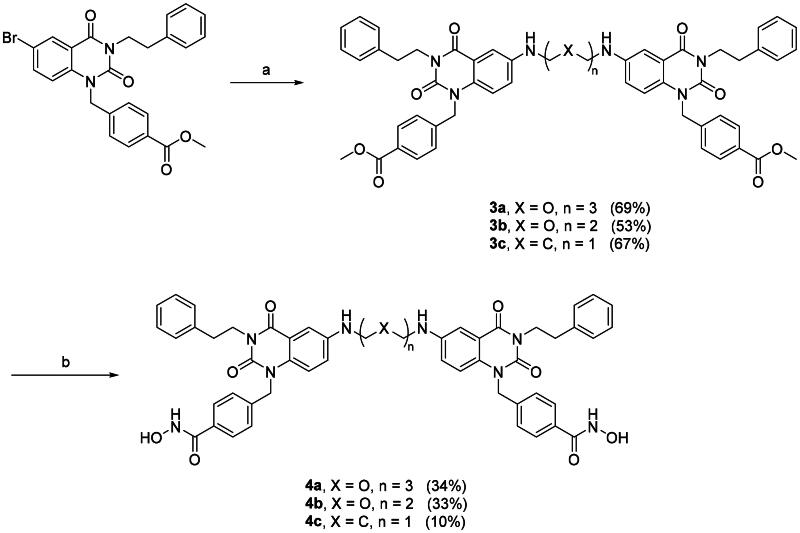
Synthesis of compounds **4a–c**. Reagents and conditions: (a) Diamine, Cs_2_CO_3_, μ-OMs dimer, BrettPhos, dioxane, reflux, 24 h; (b) NH_2_OH in THF/CH_2_Cl_2_/MeOH (2 M), rt, 2 h.

**Scheme 3.**
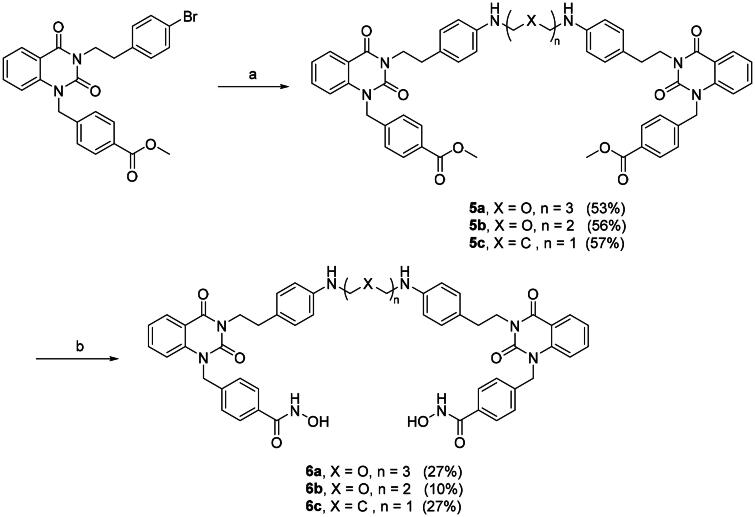
Synthesis of compounds **6a–c**. Reagents and conditions: (a) Diamine, Cs_2_CO_3_, μ-OMs dimer, BrettPhos, dioxane, reflux, 24 h; (b) NH_2_OH in THF/CH_2_Cl_2_/MeOH (2 M), rt, 2 h.

With the optimal condition in hand, **2a** and **2b** were set out to synthesise **3a–c** (Scheme 2, step a) and **5a–c** (Scheme 3, step a). Slightly lowered yields were observed in the reactions involving **2b**, this is mainly due to the halide is *para-* to a weakly electron donating group, rendering **2b** to a deactivated substrate. Dimers **3a–c** and **5a–c** were then converted into di-hydroxamic acids **4a–c** and **6a–c**, respectively.

**Scheme 4.**
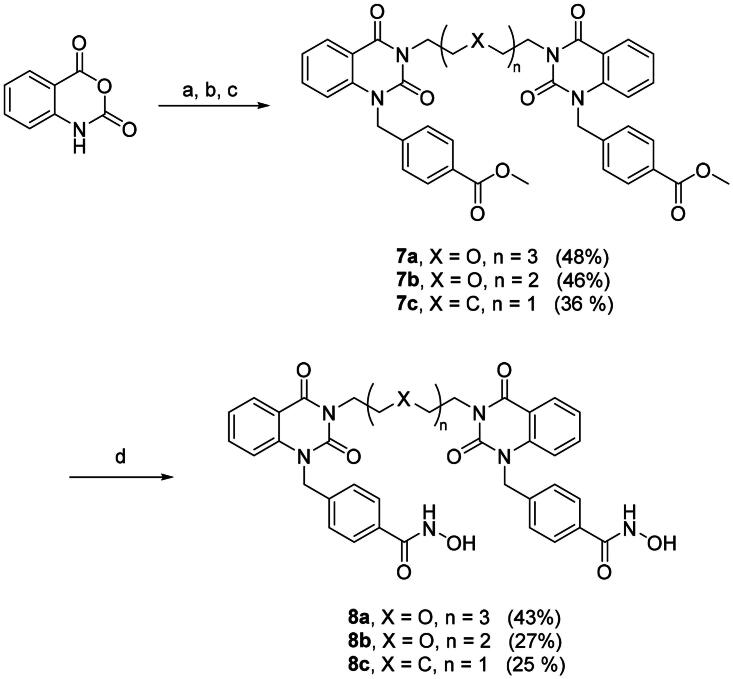
Synthesis of compounds **8a–c**. Reagents and conditions: (a) Diamine, EtOAc, rt, 1 h; (b) CDI, EtOAc, rt, 19 h; (c) K_2_CO_3_, methyl 4-(bromomethyl)benzoate, DMF, rt, 2 h; (d) NH_2_OH in THF/CH_2_Cl_2_/MeOH (2 M), rt, 2 h.

**Scheme 5.**
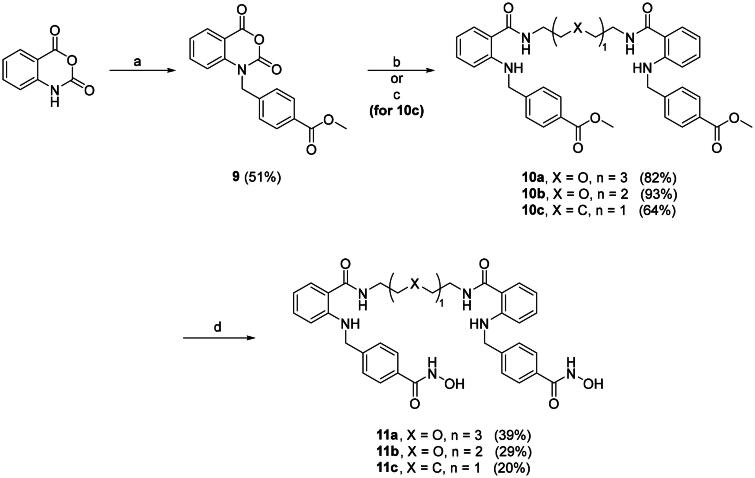
Synthesis of Compounds **11a–c**. Reagents and conditions: (a) K_2_CO_3_, methyl 4-(bromomethyl)benzoate, DMF, rt, 2 h; (b) Diamine, EtOAc, rt, 1 h; (c) Diamine, DMAC, MW 250 W, reflux, 12 min; (d) NH_2_OH in THF/CH_2_Cl_2_/MeOH (2 M), rt, 2 h

To further optimise the HDAC6 degraders, dimers **8a–c** and **11a–c** were synthesised. Utilising similar method described in Scheme 1, the di-esters **7a–c** were obtained starting with isatoic anhydride then transformed to di-hydroxamic acids **8a–c** (Scheme 4). The synthetic route for dimer **11a–c** is shown in Scheme 5, the benzyl linker was first coupled to the isatoic anhydride then ring-opening/dimerisation to yield di-esters **10a–c**. Milder condition at room temperature is suitable for PEG_3_ and PEG_2_ diamine linkers but does not seem to accommodate shorter 1,5-pentanediamine linker. Therefore, a microwave assisted method was employed for the synthesis of **10c**. Smooth transformation was achieved from **9** to **10c** under microwave irradiation despite lower yield. This is mainly due to the relatively harsher reaction condition of microwave heating. Finally, di-esters **10a–c** were treated with hydroxylamine to afford the desired aminobenzamide di-hydroxamic acid dimers **11a–c**.

Unlike the synthesis of monomeric hydroxamic acids, the reaction mixture produced from di-hydroxamic acid transformations is way more complicated. The highly polar nature of di-hydroxamic acids poses difficulties in purifying the target compounds. We found no any conventional purification methods provide satisfactory results. Therefore, we invented a novel purification method with working mechanism similar to affinity chromatography, termed Trap and Release (TNR), to address the problem. In the TNR method, a small fraction of NH2 modified silica gel connected to normal phase silica gel in series was used as the stationary phase. Eluting with MeOH/CH_2_Cl_2 _= 10% washed out most side products, leaving the desired di-hydroxamic acid along with small amount of impurities trapped in the NH2-modified silica gel due to electrostatic attraction. When most side products had been eluted, the mobile phase was switched to MeOH/CH_2_Cl_2 _= 0–5% containing 0.1% trifluoroacetic acid. Trifluoroacetic acid released the trapped di-hydroxamic acid *via* -NH_2_ salt forming competition **(**Figure S1**)**. Finally, the eluted pure target compound was concentrated and then precipitated out with NaHCO_3(aq.)_ treatment. All pure target di-hydroxamic acid dimers were successfully obtained *via* the TNR purification.

### Designing HDAC6 inhibitor dimers

**Figure 2.**
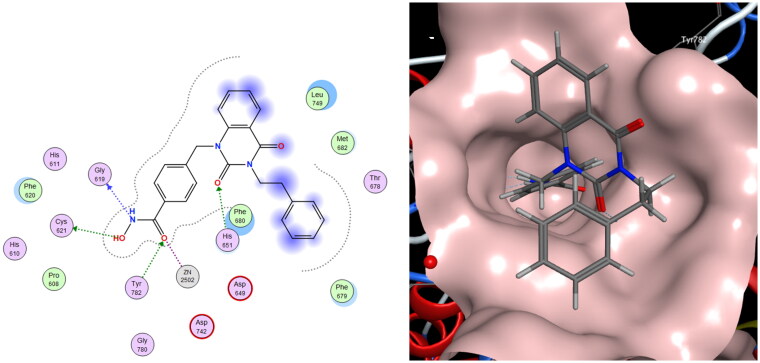
Molecular docking analysis of **1** to *h*HDAC6 CD2 *via* MOE. (Left) Interaction diagram of **1** with the binding pocket. (Right) Top-down view of **1** docking to *h*HDAC6 CD2 with the receptor coloured in pink. (PDB ID: 5EDU).

Parameters including ligand, conjugation site, linker length and types are all crucial in designing proximity-inducing drugs^25^. A highly selective HDAC6 inhibitor **1** from our in-house library served as a good starting point^17^. There are no known HDAC6-compound **1** complex crystal structures been reported. Therefore, molecular docking analysis was performed to gain structural insights in determining the conjugation sites. Based on the docking results shown in Figure 2, position 5 and 6 of the quinazolin-2,4-dione and the entire phenethyl moiety are exposed, which serve as ideal conjugation sites for dimerisation without disrupting receptor binding. Position 6 of the quinazolin-2,4-dione and the *para*-position of the phenethyl moiety were chosen as conjugation sites to generate two series of compound **1-**based dimer each protrudes in different orientations. Three linkers with length ranging from roughly 7–13 atoms, 1,5-pentanediamine, PEG_2_-diamine, and PEG_3_-diamine, were selected for optimal chain length screening.

### Bioactivities and physical properties of compound 1-based HDAC6 inhibitor dimers

**Figure 3.**
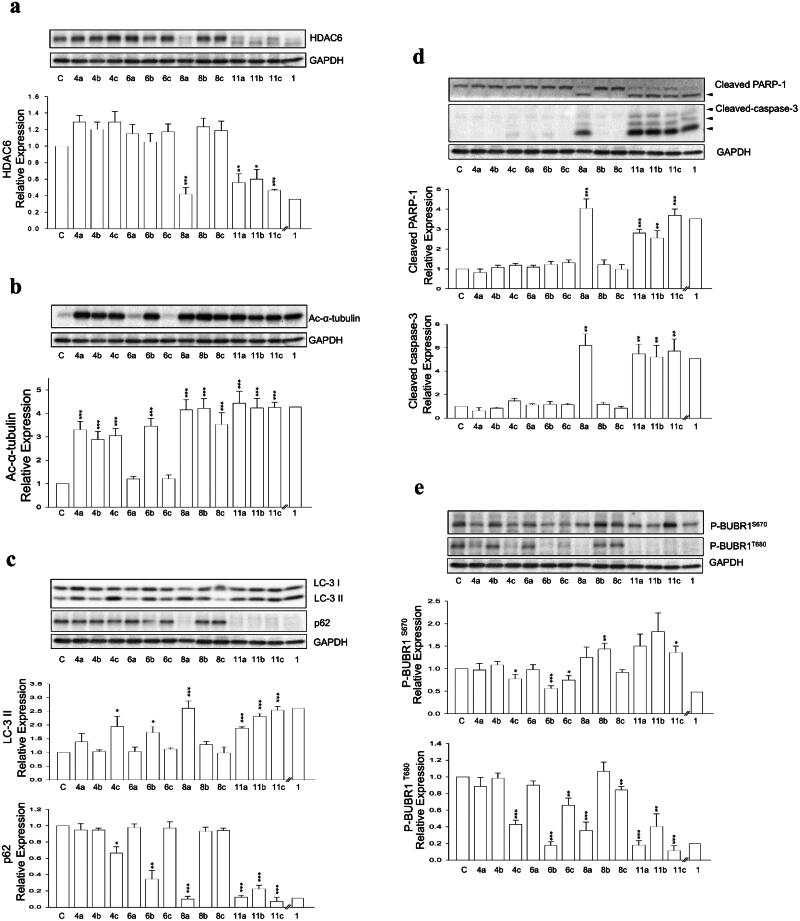
Western blotting analysis of (a) HDAC6 protein in PC-3 cells treated with various dimer compounds at 30 μM for 24 h, (b) Ac-α-tubulin protein in PC-3 cells treated with various dimer compounds at 30 μM for 24 h, (c) LC-3 I, LC-3 II, and p62 proteins in PC-3 cells treated with various dimer compounds at 30 μM for 24 h, (d) PARP-1 and Caspase-3 proteins in PC-3 cells treated with various dimer compounds at 30 μM for 24 h, (e) p-BUBR1^S670^ and p-BUBR1^T680^ proteins in PC-3 cells treated with various dimer compounds at 30 μM for 24 h. Glyceraldehyde 3-phosphate dehydrogenase (GAPDH) was used as the loading control.

Bioactivities and physical properties data of two series of **1**-based dimers, **4a–c** and **6a–c**, were reported in Table 2. Conjugation at the 6-position impaired inhibition activity towards HDAC6 and exhibited limited cytotoxicity towards both PC-3 and A549 cell lines. This might possibly due to steric arises from protein backbone or residues outside of the receptor that were not considered and shown in docking analysis. HDAC6 degradation was not observed in **4a–c** as well (Figure 3a). Despite not as potent as compound **1**, significant decrease in HDAC6 activity was still observed for all three compounds (**4a–c**) as acetylated α-tubulin levels appreciably raised (Figure 3(b)). HDAC inhibition profile indicated **4b** was slightly selective over HDAC1 (Table 2).

**Table 2.** Biological activities and physical properties of ****1**, 4a–c, 6a–c, 8a–c**, and **11a–c**.

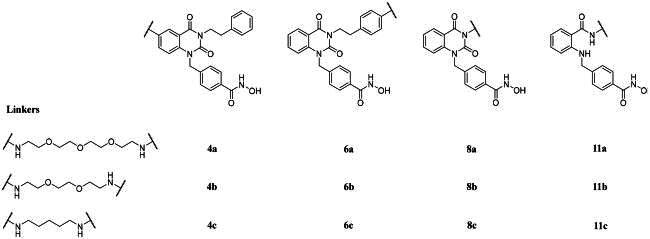							
	IC_50_ (nM)			GI_50_ con. ± SEM (μM)			
Cpd	HDAC6	HDAC1	HDAC1/6 Selectivity	PC-3	A549	cLogP	TPSA
**1**	4	11,160	2790	3.5	8.8	2.7	77.2
**4a**	92	N.D.	–	>30	>30	5.3	199.3
**4b**	816	>10,000	>12	>30	>30	5.5	191.1
**4c**	96	N.D.	–	21.4 ± 0.3	>30	6.2	174.5
**6a**	>10,000	N.D.	–	>30	>30	4.5	199.3
**6b**	33	9566	283	5.5 ± 0.7	9.1 ± 1.8	4.7	191.1
**6c**	1560	>10,000	>6	13.5 ± 3.8	13.7 ± 1.5	5.4	174.5
**8a**	12	3327	273	14.7 ± 1.3	>30	1.0	179.2
**8b**	2	3188	1386	>30	>30	1.2	170.9
**8c**	27	N.D.	–	>30	>30	2.6	154.4
**11a**	4	2597	577	5.9 ± 0.7	17.3 ± 0.2	1.2	184.8
**11b**	3	1271	326	11.3 ± 0.2	17.9 ± 0.4	1.3	176.5
**11c**	15	1458	95	8.2 ± 0.1	6.6 ± 0.1	2.0	159.9

PSA units are in Å^2^. cLogP and TPSA were calculated *via* MolSoft. N.D.: not determined

In spite of the unsatisfactory results of **4a–c**, the other dimer series **6a–c** that conjugate on the opposite side showed intriguing outcomes. Compared to **1**, **6b** and **6c** showed compromised HDAC6 inhibition activity. However, they were in the same ballpark as **1** when it comes to cytotoxicity. HDAC inhibition profile suggested **6b** and **6c** were considered selective over HDAC1 (Table 2), ruling out the possibility that the observed cytotoxicity was due to HDAC1 inhibition. These results indicated there might exist additional interactions other than solely enzymatic activity inhibition in cells. Although showing exciting biological activities, no significant degradation of HDAC6 was observed (Figure 3(a)). Moreover, **6a–c** showed concerning physical property, with clogP values on the higher end when considering Lipinski’s rule of five. In fact, poor solubilities of **6a** and **6c** were observed when conducting assays, which resulted in the compromised enzyme inhibition activity and unenhanced acetylated α-tubulin in western blot assay.

### Optimisation of HDAC6 inhibitor dimers

More promising compounds **6a–c** were chosen to proceed to further development. Docking studies (Figure 2) revealed that the entire phenethyl moiety was exposed without any interaction with the receptor. Therefore, the hydrophobic phenethyl moiety was cropped out to increase hydrophilicity and solubility. Taking out the phenethyl group yielded compounds **8a–c**. Deleting the phenethyl greatly improved the clogP values of **8a–c** by up to 3.5 numerical values (Table 2). Dimers **8a–c** all exhibited excellent HDAC6 enzymatic inhibition, mild to great HDAC1/6 selectivity (**8a** and **8b**), and significantly higher Ac-α-tubulin level (Table 2, Figure 3(b)). However, the cytotoxicity of **8a–c** towards PC-3 and A549 cell lines was greatly reduced. We suspected this may be due to compounds **8a–c** being too hydrophilic, as the clogP values were now on the lower end, which compromised their cell permeability. Nevertheless, shown in Table 2 is a trend where increasing linker length restores cytotoxicity. Researches have shown that bivalent functional molecules such as PROTACs adapted a folded state in physiological conditions, analogous to that of protein folding^26^^,^^27^. The folded state generally has smaller overall volume and aids the cell permeation. The restoration of cytotoxicity at longer linker length might be due to this phenomenon. Agreeing with that, degradation of HDAC6 was observed in **8a** (Figure 3(a)). Removal of the phenethyl moiety decreased the distance between the two quinazolin-2,4-dione cores by 10 to 12 atoms. We surmised that the distances between two monomers in **8a–c** were most suitable for protein–protein interaction (PPI) interface between two HDAC6s.

### Flexible structure improves biological properties

We sought to further optimise both the physical and biological properties of these dimers. Therefore, we bestowed upon dimers **8a–c** extra rotational degrees of freedom to promote folding for improved cell permeability. This was achieved by opening the quinazolin-2,4-dione ring, affording a series of 2-amino benzamide-based dimer **11a–c**. While showing similar clogP and TPSA values to that of dimers **8a–c**, ring-opened dimers **11a–c** showed excellent cytotoxicity and inhibition activity for HDAC6 accompanied by good HDAC1/6 selectivity **(**Table 2). Along with their promising bioactivities, all three dimers exhibited moderate yet significant HDAC6-degrading activity (Figure 3(a)).

### HDAC6i dimer exhibit binding mode similar to compound 1

**Figure 4.**
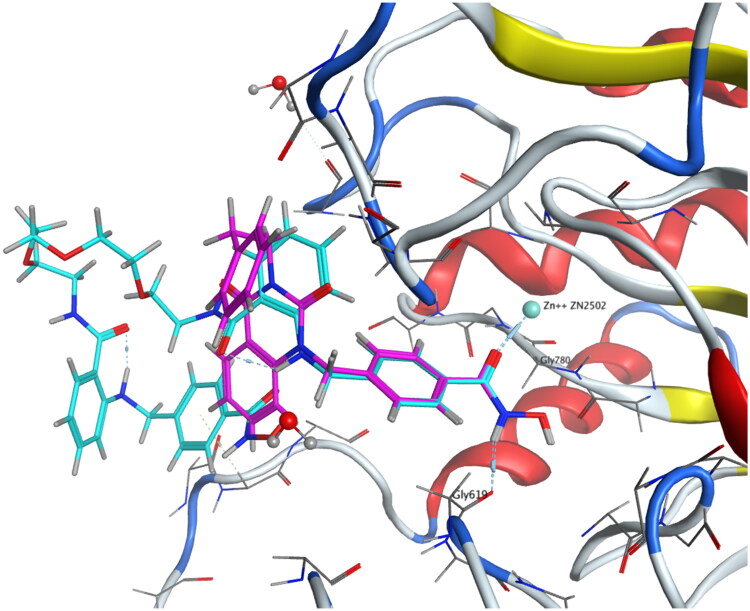
Molecular docking studies of 1 (coloured in pink) and 11a (coloured in cyan) to *h*HDAC6 CD2 under rigid receptor refinement *via* MOE. (PDB 5EDU).

To clarify the binding mode of the dimers, molecular docking study was performed. Our best dimer **11a** (HDAC6 IC_50_ 4.5 nM, HDAC1/6 selectivity 577, PC-3 GI_50_ 5.9 μM, A549 GI_50_ 17.3 μM) and compound **1** were docked against *h*HDAC6 CD2 (PDB 5EDU), shown in Figure 4. Docking results revealed the short and wide benzyl linker in both compounds adopted identical binding mode. This might correspond to the excellent HDAC6 inhibition activity observed. When looking at the CAP group, **11a** exhibited opposite orientation compared to **1**. This difference might explain the slightly decreased selectivity of **11a** over HDAC1.

### HDAC6i dimers increase Sub-G1 cell cycle population and programmed cell death in human prostate cancer cells

**Figure 5.**
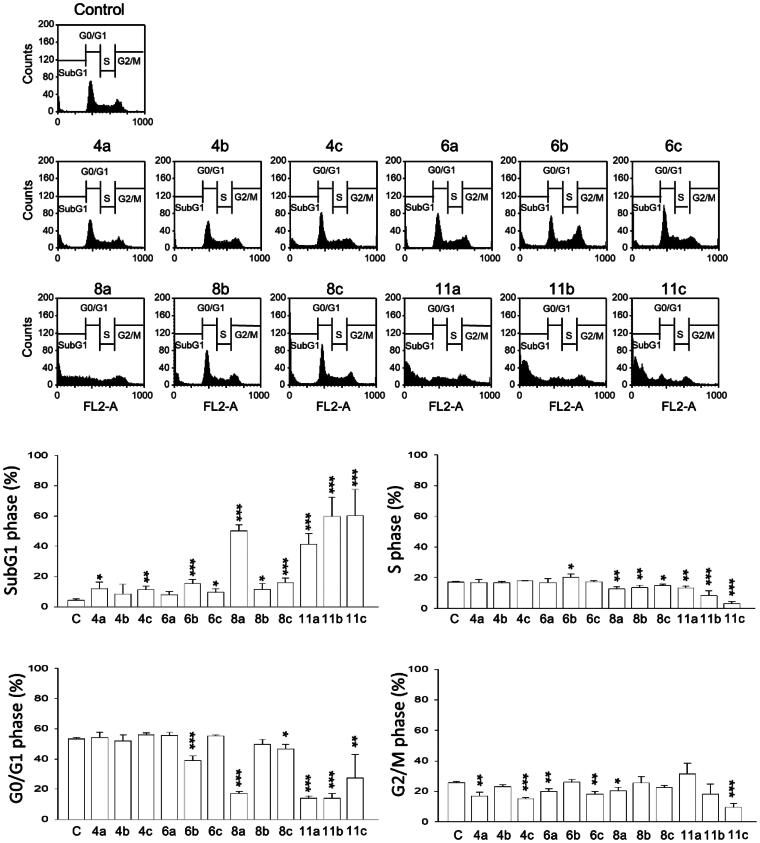
Flow cytometric cell cycle analysis of PC-3 cells treated with various dimer compounds at 30 μM for 24 h.

Given the successful induction of auto-degradation by these dimers, more detailed studies on their interactions with human prostate cancer cell lines (PC-3) were conducted. Two types of programmed cell death (PCD), autophagy and apoptosis, were found to parallel to HDAC6 auto-degradation. Shown in Figure 3(c) is the Western blotting analysis of autophagy-related proteins LC-3 II and p62. Dimers that induced HDAC6 auto-degradation (**8a, 11a–c**) all showed strong autophagy induction, evident by increased LC-3 II protein level and decreased p62 expression. Surprisingly, compound **6b** which did not degrade HDAC6 also exhibited signs of inducing autophagy. Nevertheless, **6b** did not induce apoptosis like **8a** and **11a–c**, suggesting that the autophagy induced by **6b** may be caused by alternative mechanisms. Apoptosis biomarkers, such as cleavage of PARP-1 (a substrate of Caspase-3) and Caspase-3 activation, were analysed with Western blotting and shown in Figure 3(d). A strong correlation between induced apoptosis and HDAC6 degradation was observed. Flow cytometric analysis indicated HDAC6 degrading dimers exerted their apoptotic property *via* an increase at sub-G1 population (Figure 5). Further examination showed increased BUBR1 phosphorylation at S670, indicating the interference of cell cycle progression by interrupting kinetochore attachment (Figure 3(e)). However, how HDAC6 degradation interacted with these pathways were yet to be elucidated.

## Conclusions

**Figure 6.**
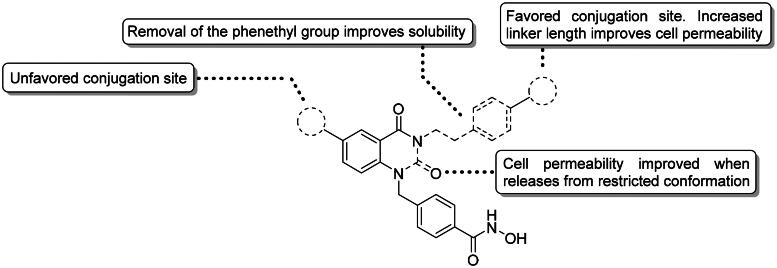
Graphical conclusion of quinazolin-2,4-dione-based HDAC6 inhibitor dimers.

Briefly, we have not only successfully proven HDAC6 do possess ubiquitin E3 ligase activity by developing series of HDAC6 chemical degrader, but also demonstrated that HDAC6 is domesticable in PROTAC designing. Analogous to homo-PROTACs, selective HDAC6 inhibitor dimers induced HDAC6 auto-degradation. Alongside with the novel discovery in the biological properties of HDAC6, we have described a palladium catalysed synthesis for hydroxamic acid dimers. A simple and efficient purification method for these dimers was also developed and hereby reported. Graphically depicted in Figure 6, the 6 positions of the quinazolin-2,4-dione generally do not tolerate any conjugations; while the *para*-position of the phenethyl ring serves as a good conjugation site. The highly hydrophobic phenethyl group is not necessary for HDAC6 inhibition, removal of this group greatly improves dimer solubility. The acceptable linker length between the two quinazolin-2,4-dione cores is approximately 5–12 atoms. Permeability is also an important factor in exerting cytotoxicity and degradation activity to cells. Increased dimer flexibility promotes self-folding, leading to reduced overall volume and better cell permeability. These dimers exhibit good to excellent HDAC1/6 selectivity and adopt similar binding mode to their parent compound. HDAC6 degrading dimers induce PCD and interfere with cell cycle progression. Despite more detailed mechanistic elucidations were required to verify our findings, this study suggested the potential of manipulating HDAC6 as novel mechanism for drug development.

## Experimental section

### General information for synthetic methods

All solvents used were purchased from Duksan, JT Baker, Merck, ECHO, or Mallinckrodt; they were ACS grade and used without further purification. Anhydrous solvents were prepared using an SP-1 stand-alone solvent purification system (LC Technology Solution). Unless otherwise is noted, all air- and moisture-sensitive reaction was carried out under an atmosphere of argon using flame-dried glassware. Chemicals were purchased from Acros, Aldrich, Alfa Aesar, AK scientific, Carbosynth, Fluka, KM3, Matrix, and TCI and used as supplied. Reaction progress was monitored by TLC on Merck Kieselgel 60 F_254_ plates, Merck Kieselgel 60 RP-18 F_25_, or Agela Technologies NH_2_ silica GF254 plates. Microwave reactions were carried out using the CEM Discover 1.0 and 2.0 platform. Organic solutions were concentrated under reduced pressure (about 60 Torr) by rotary evaporation. Flash column chromatography was carried out on Chromatorex MB 70-40/75 spherical silica gel (Fuji Silysia Chemical LTD.) or Chromatorex NH MB 100-40/75 silica gel (Fuji Silysia Chemical LTD.) using medium pressure liquid chromatography (MPLC) AS-204P (Agela Technologies).^1^H and ^13^C NMR spectra were obtained on a Bruker DPX-200, AV-400, or AVIII-600 operating at 200, 400, or 600 MHz. Chemical shifts were referenced to the central peak of the DMSO-*d*_6_ spectrum (2.49 ppm for ^1^H NMR and 39.5 ppm for ^13^C NMR). Multiplicity of peaks in ^1^H NMR is defined by the abbreviations s (singlet), d (doublet), t (triplet), q (quartet), and m (multiplet). Melting point was measured by Fargo MP-2D without calibration. Mass spectroscopy was performed on a Bruker Esquire 2000 using electrospray ionisation (ESI) and analysed by quadrupole detector. High-resolution mass spectroscopy was performed on a Thermo Scientific Orbitrap Elite using ESI and an ion trap detector. High-performance liquid chromatography was carried out on a Shimadzu HPLC with LC-20AT pump, SPD-M20A photodiode array detector, SIL-20A autosampler, and Phenomenex Kinetex^®^ 3.5 µm XB-C18 100 Å, LC Column 100 × 4.6 mm. Analyte was eluted by 0.1%FA in ACN/0.1%FA in H_2_O = 10/90–100/0.

#### General Procedure A for the synthesis of halide substituted compound 1 (quinazolin-2,4-dione based HDAC6 inhibitor)

Substituted or unsubstituted isatoic anhydride (1 equiv.) was stirred in EtOAc (1 M) at room temperature for 10 min. To the solution was added substituted or unsubstituted phenethylamine (1.1 equiv.) and kept stirring for 1 h. A solution of 1,1′-carbonyldiimidazole (1.5 equiv.) dissolved in EtOAc (1 M) was added dropwise into the reaction mixture then allowed to stir for 19 h. The solids were filtered, dried *in vacuo*, then mixed with K_2_CO_3_ (3 equiv.) and methyl 4-(bromomethyl)benzoate (1.05 equiv.) in acetone (0.5 M). After stirring at 60 °C for 3 h, the solvent was evaporated. The remaining solids were suspended in H_2_O then extracted twice with CH_2_Cl_2_ and twice with EtOAc, sequentially. Combined organic layer was dried over MgSO_4_, filtered, dried *in vacuo*. The resulting solid was triturated with MeOH under sonication to give the desired product.

*Methyl 4-((6-bromo-3,4-dihydro-2,4-dioxo-3-phenethylquinazolin-1(2H)-yl)methyl)benzoate (2a)*. Following general Procedure A, 5-bromoisatoic anhydride (10 g, 41.3 mmol) and phenethylamine (5.5 g, 45.4 mmol) were reacted to give the product as a white solid (13.675 g, 67%). mp = 112–114 °C; ^1^H NMR (600 MHz, DMSO-*d*_6_): *δ* 8.12 (d, *J* = 2.6 Hz, 1H), 7.90 (d, *J* = 8.6 Hz, 2H), 7.80 (dd, *J* = 8.8, 2.5 Hz, 1H), 7.36 (d, *J* = 8.5 Hz, 2H), 7.31–7.27 (m, 2H), 7.25–7.16 (m, 4H), 5.42 (s, 2H), 4.21 (t, 7.7 Hz, 2H), 3.83 (s, 3H), 2.94 (t, 7.7 Hz, 2H); ^13^C NMR (150 MHz, DMSO-*d*_6_): *δ* 166.0, 159.9, 150.4, 141.5, 138.8, 138.4, 137.7, 129.9, 129.5, 128.7, 128.5, 126.7, 126.4, 117.4, 117.0, 114.9, 52.2, 46.4, 42.7, 33.0; ESIMS(+) m/z 493.0 [M + H]^+^.

*Methyl 4-((3-(4-bromophenethyl)-3,4-dihydro-2,4-dioxoquinazolin-1(2H)-yl)methyl)benzoate (2b)*. Following general Procedure A, isatoic anhydride (10 g, 61.3 mmol) and 4-bromophenethylamine (13.5 g, 67.4 mmol) were reacted to give the product as a white solid (18.895 g, 62%). mp = 159–161 °C; ^1^H NMR (600 MHz, DMSO-*d*_6_): *δ* 8.08 (dd, *J* = 8.0, 2.0 Hz, 1H), 7.92 (d, *J* = 8.3 Hz, 2H), 7.65 (t, *J* = 7.7, 1H), 7.47 (d, *J* = 8.0 Hz, 2H), 7.34 (d, *J* = 8.7 Hz, 2H), 7.28 (t, *J* = 8.0 Hz, 1H), 7.23–7.17 (m, 3H), 5.42 (s, 2H), 4.24 (t, *J* = 7.6 Hz, 2H), 3.83 (s, 3H), 2.94 (t, *J* = 7.6 Hz, 2H); ESIMS(+) m/z 493.0 [M + H]^+^.

#### *General* Procedure B *for Buchwald–Hartwig C-N coupling*

Aryl halide (1 equiv.), Cs_2_CO_3_ (3 equiv.), μ-OMs dimer (2.5 mol%), and BrettPhos (5 mol%) were added to a flask under Ar. To the flask was injected a solution of diamine linker (0.6 equiv.) dissolved in dioxane (0.1 M). After stirring under reflux for 16 h, the reaction mixture was let cooled then filtered through celite eluting with CH_2_Cl_2_. The filtrate was dried *in vacuo* then purified with MPLC to afford the product.

*Methyl 4-{[6-(2-{2-[2-(2-{1-[(4-methoxycarbonylphenyl)methyl]-2,4-dioxo-3-phenethylquinazolin-6-ylamino}ethoxy)ethoxy]ethoxy}ethylamino)-2,4-dioxo-3-phenethylquinazolin-1-yl]methyl}benzoate (3a)*. Following General Procedure B, a mixture of **2a** (1 g, 2 mmol), Cs_2_CO_3_ (1.98 g, 6 mmol), 1,11-Diamino-3,6,9-trioxaundecane (234 mg, 1.2 mmol), μ-OMs dimer (55 mg, 0.05 mmol), and BrettPhos (54 mg, 0.1 mmol), and dioxane (20 ml) was stirred under reflux for 16 h. The crude mixture was purified by MPLC (EtOAc/*n-*Heptane = 80%) to provide the title compound as a yellow solid (716 mg, 69%). mp = 195–197 °C; ^1^H NMR (200 MHz, DMSO-*d*_6_): *δ* 7.88 (d, *J* = 8.0 Hz, 4H), 7.36–7.12 (m, 16H), 7.00–6.91 (m, 4H), 5.81 (t, *J* = 5.2 Hz, 2H), 5.34 (s, 4H), 4.21 (t, *J* = 7.8 Hz, 4H), 3.82 (s, 6H), 3.60–3.44 (m, 12H), 3.23–3.07 (m, 4H), 2.91 (t, *J* = 7.8 Hz, 4H); ^13^C NMR (150 MHz, DMSO-*d*_6_): *δ* 165.9, 161.1, 150.1, 144.9, 142.4, 138.6, 129.9, 129.5, 128.7, 128.5, 128.4, 126.7, 126.3, 121.0, 115.8, 115.7, 107.4, 69.8, 69.7, 68.9, 52.1, 46.0, 42.8, 42.4, 33.2; ESIMS(+) m/z 1017.0 [M + H]^+^.

*Methyl 4-[(6-{2-[2-(2-{1-[(4-methoxycarbonylphenyl)methyl]-2,4-dioxo-3-phenethylquinazolin-6-ylamino}ethoxy)ethoxy]ethylamino}-2,4-dioxo-3-phenethylquinazolin-1-yl)methyl]benzoate (3b)*. Following General Procedure B, a mixture of **2a** (1 g, 2 mmol), Cs_2_CO_3_ (1.98 g, 6 mmol), 2-(2-(2-aminoethoxy)ethoxy)ethanamine (180 mg, 1.2 mmol), μ-OMs dimer (55 mg, 0.05 mmol), and BrettPhos(54 mg, 0.1 mol), and dioxane (20 ml) was stirred under reflux for 16 h. The crude mixture was purified by MPLC (EtOAc/*n-*Heptane = 80%) to provide the title compound as a yellow solid (518 mg, 53%). mp = 172–174 °C; ^1^H NMR (600 MHz, DMSO-*d*_6_): *δ* 7.92–7.85 (m, 4H), 7.34–7.13 (m, 16H), 7.00–6.92 (m, 4H), 5.83 (s, 2H), 5.33 (s, 4H), 4.21 (s, 4H), 3.82 (s, 6H), 3.58–3.49 (m, 8H), 3.20–3.12 (m, 4H), 2.92 (s, 4H); ^13^C NMR (150 MHz, DMSO-*d*_6_): *δ* 169.7, 166.0, 161.1, 150.2, 144.9, 142.4, 138.6, 129.9, 129.5, 128.7, 128.5, 128.4, 126.7, 126.3, 121.0, 115.8, 115.7, 107.4, 69.7, 69.0, 52.1, 46.0, 42.9, 42.4, 33.3; ESIMS(+) m/z 973.0 [M + H]^+^.

*Methyl 4-{[6-(5-{1-[(4-methoxycarbonylphenyl)methyl]-2,4-dioxo-3-phenethylquinazolin-6-ylamino}pentylamino)-2,4-dioxo-3-phenethylquinazolin-1-yl]methyl}benzoate (3c)*. Following General Procedure B, a mixture of **2a** (1 g, 2 mmol), Cs_2_CO_3_ (1.98 g, 6 mmol), cadaverine (124 mg, 1.2 mmol), μ-OMs dimer (55 mg, 0.05 mmol), and BrettPhos(54 mg, 0.1 mol), and dioxane (20 ml) was stirred under reflux for 24 h. The crude mixture was purified by MPLC (EtOAc/*n-*Heptane = 60%) to provide the title compound as a pale solid (695 mg, 67%). mp = 181–183 °C; ^1^H NMR (600 MHz, MeOD): *δ* 7.90 (d, *J* = 6.5 Hz, 4H), 7.35–7.11 (m, 16H), 7.01–6.89 (m, 4H), 5.83 (s, 2H), 5.35 (s, 4H), 4.22 (s, 4H), 3.83 (s, 6H), 3.03–2.96 (m, 4H), 2.96–2.89 (m, 4H), 1.61–1.52 (m, 4H), 1.49–1.39 (m, 2H); ^13^C NMR (150 MHz, DMSO-*d*_6_): *δ* 166.0, 161.1, 150.2, 145.1, 142.4, 138.6, 129.7, 129.5, 128.7, 128.6, 128.4, 126.7, 126.3, 120.9, 115.9, 115.7, 107.1, 52.1, 46.0, 43.0, 42.4, 33.3, 28.3, 24.3; ESIMS(+) m/z 927.0 [M + H]^+^.

#### Trap and release (TNR) purification

Prior to a normal phase silica gel cartridge, a 4 g NH_2_ silica cartridge was connected in series. Crude product was eluted with MeOH/CH_2_Cl_2_ = 10% for 10 min to trap the desired product in the NH2 silica gel then eluted with MeOH/CH_2_Cl_2_ = 0–5% containing 0.1% TFA to wash out the product. Fractions collected were pooled in a flask then concentrated *in vacuo*. Saturated NaHCO_3(aq.)_ was added to neutralise remaining TFA. Precipitate formed was filtered and dried *in vacuo* to give the desired product.

#### General Procedure C for dihydroxamic acid synthesis

To a beaker was added NH_2_OH·HCl (40 equiv.) and solvent cocktail (2 M, 45% CH_2_Cl_2_, 45% THF, 10% MeOH). NaOMe (40 equiv.) was added to the beaker in portions under ice bath and stirred from 0 °C to room temperature for 30 min. The resulting slurry was filtered then added to the diester (1 equiv.) and stirred at room temperature for 2 h. Upon completion, TFA (2 ml) was added to quench the reaction then dried *in vacuo*. TNR method was applied to isolate the desired product.

*4-{[6-(2-{2-[2-(2-{1-[(4-Hydroxamophenyl)methyl]-2,4-dioxo-3-phenethylquinazolin-6-ylamino}ethoxy)ethoxy]ethoxy}ethylamino)-2,4-dioxo-3-phenethylquinazolin-1-yl]methyl}benzohydroxamic acid (4a)*. By following general Procedure C, **3a** (200 mg, 0.2 mmol) was converted into **4a** as a pink solid (68 mg, 34%). mp = 126–128 °C; ^1^H NMR (600 MHz, DMSO-*d*_6_): *δ* 11.17 (s, 2H), 9.00 (s, 2H), 7.68 (d, *J* = 8.2 Hz, 4H), 7.32–7.15 (m, 16H), 7.02–6.94 (m, 4H), 5.30 (s, 4H), 4.21 (t, *J* = 7.7 Hz, 4H), 3.56–3.50 (m, 12H), 3.19–3.14 (m, 4H), 2.93 (t, *J* = 7.2 Hz, 4H); ^13^C NMR (150 MHz, DMSO-*d*_6_): *δ* 163.9, 161.1, 150.2, 144.4, 139.9, 138.6, 131.7, 130.2, 128.7, 128.4, 127.2, 126.4, 126.3, 121.3, 115.8, 107.9, 69.8, 69.7, 68.8, 45.9, 43.1, 42.4, 33.2; HRMS (ESI): calcd for [C_56_H_59_O_11_N_8_] (M + H)^+^, 1019.4298; found, 1019.4298. HPLC 98.2% (t_R_ = 12.71).

*4-[(6-{2-[2-(2-{1-[(4-Hydroxamophenyl)methyl]-2,4-dioxo-3-phenethylquinazolin-6-ylamino}ethoxy)ethoxy]ethylamino}-2,4-dioxo-3-phenethylquinazolin-1-yl)methyl]benzohydroxamic acid (4b)*. By following general Procedure C, **3b** (200 mg, 0.21 mmol) was converted into **4b** as a pink solid (67 mg, 33%). mp = 206–208 °C; ^1^H NMR (600 MHz, DMSO-*d*_6_): *δ* 11.17 (s, 2H), 9.00 (s, 2H), 7.88–6.72 (m, 24H), 5.90–5.66 (m, 2H), 5.29 (s, 4H), 4.21 (s, 4H), 3.66–3.45 (m, 8H), 3.20–3.07 (m, 4H), 2.93 (s, 4H); ^13^C NMR (150 MHz, DMSO-*d*_6_): *δ* 161.1, 150.2, 144.8, 141.7, 140.0, 138.6, 129.9, 128.7, 128.4, 127.2, 126.8, 126.4, 126.3, 121.0, 120.6, 115.8, 107.4, 69.7, 68.9, 45.9, 42.8, 42.4, 33.2; HRMS (ESI): calcd for [C_54_H_55_O_10_N_8_] (M + H)^+^, 975.4036; found, 975.4033. HPLC 96.2% (t_R_ = 10.04).

*4-{[6-(5-{1-[(4-Hydroxamophenyl)methyl]-2,4-dioxo-3-phenethylquinazolin-6-ylamino}pentylamino)-2,4-dioxo-3-phenethylquinazolin-1-yl]methyl}benzohydroxamic acid (4c)*. By following general Procedure C, **3c** (200 mg, 0.22 mmol) was converted into **4c** as a pink solid (20 mg, 10%). mp = 177–179 °C; ^1^H NMR (600 MHz, DMSO-*d*_6_): *δ* 11.19 (s, 2H), 9.00 (s, 2H), 7.88 (d, *J* = 8.1 Hz, 2H), 7.68 (d, *J* = 8.1 Hz, 2H), 7.32–7.10 (m, 16H), 7.03–6.88 (m, 4H), 5.39–5.22 (m, 4H), 4.28–4.15 (m, 4H), 3.04–2.97 (m, 4H), 2.97–2.88 (m, 4H), 1.61–1.52 (m, 4H), 1.48–1.39 (m, 2H); ^13^C NMR (150 MHz, DMSO-*d*_6_): *δ* 167.2, 161.1, 150.2, 138.6, 129.6, 128.7, 128.4, 127.2, 126.6, 126.4, 126.3, 115.8, 46.0, 43.0, 42.4, 33.3, 28.2, 24.3; ESIMS(+) m/z 914.39 [M - N]^+^. HPLC 98.3% (t_R_ = 14.81).

*Methyl 4-({3-[2-(4-{2-[2-(2-{2-[4-(2-{1-[(4-methoxycarbonylphenyl)methyl]-2,4-dioxoquinazolin-3-yl}ethyl)phenylamino]ethoxy}ethoxy)ethoxy]ethylamino}phenyl)ethyl]-2,4-dioxoquinazolin-1-yl}methyl)benzoate (5a)*. Following General Procedure B, a mixture of **2b** (1 g, 2 mmol), Cs_2_CO_3_ (1.98 g, 6 mmol), 1,11-Diamino-3,6,9-trioxaundecane (234 mg, 1.2 mmol), μ-OMs dimer (55 mg, 0.05 mmol), and BrettPhos(54 mg, 0.1 mol), and dioxane (20 ml) was stirred under reflux for 16 h. The crude mixture was purified by MPLC (EtOAc/*n-*Heptane = 80%) to provide the title compound as a pale-yellow solid (556 mg, 69%). mp = 119–121 °C; ^1^H NMR (600 MHz, DMSO-*d*_6_): *δ* 8.06 (d, *J* = 8.0 Hz, 2H), 7.90 (d, *J* = 7.6 Hz, 4H), 7.62 (t, *J* = 7.6 Hz, 2H), 7.34 (d, *J* = 8.0 Hz, 4H), 7.25 (t, *J* = 7.6 Hz, 2H), 7.19 (d, *J* = 8.2 Hz, 2H), 6.93 (d, *J* = 8.0 Hz, 4H), 6.51 (d, *J* = 8.0 Hz, 4H), 5.41 (s, 4H), 5.36 (s, 2H), 4.14 (t, *J* = 8.0 Hz, 4H), 3.82 (s, 6H), 3.56–3.50 (m, 12H), 3.17–3.11 (m, 4H), 2.77 (t, *J* = 8.0 Hz, 4H); ^13^C NMR (150 MHz, DMSO-*d*_6_): *δ* 165.9, 160.9, 150.6, 147.2, 141.9, 139.4, 125.2, 129.5, 129.2, 128.6, 128.0, 126.7, 125.3, 123.0, 115.2, 114.7, 112.2, 69.8, 69.7, 69.1, 52.1, 46.2, 42.9, 42.8, 32.3; ESIMS(+) m/z 1017.0 [M + H]^+^.

*Methyl 4-{[3-(2-{4-[2-(2-{2-[4-(2-{1-[(4-methoxycarbonylphenyl)methyl]-2,4-dioxoquinazolin-3-yl}ethyl)phenylamino]ethoxy}ethoxy)ethylamino]phenyl}ethyl)-2,4-dioxoquinazolin-1-yl]methyl}benzoate (5b)*. Following General Procedure B, a mixture of **2b** (1 g, 2 mmol), Cs_2_CO_3_ (1.98 g, 6 mmol), 2-(2-(2-aminoethoxy)ethoxy)ethanamine (180 mg, 1.2 mmol), μ-OMs dimer (55 mg, 0.05 mmol), and BrettPhos(54 mg, 0.1 mol), and dioxane (20 ml) was stirred under reflux for 16 h. The crude mixture was purified by MPLC (EtOAc/*n-*Heptane = 80%) to provide the title compound as a pale-yellow solid (556 mg, 56%). mp = 102–104 °C; ^1^H NMR (600 MHz, DMSO-*d*_6_): *δ* 8.06 (dd, *J* = 7.7, 1.5 Hz, 2H), 7.90 (d, *J* = 8.9 Hz, 4H), 7.65–7.60 (m, 2H), 7.34 (d, *J* = 8.4 Hz, 4H), 7.25 (t, *J* = 7.7 Hz, 2H), 7.19 (d, *J* = 8.4 Hz, 2H), 6.93 (d, *J* = 8.6 Hz, 4H), 6.51 (d, *J* = 8.5 Hz, 4H), 5.41 (s, 4H), 5.37 (t, *J* = 5.9 Hz, 2H), 4.14 (t, *J* = 8.0 Hz), 3.82 (s, 6H), 3.57–3.52 (m, 8H), 3.14 (q, *J* = 5.7 Hz, 4H), 2.77 (t, *J* = 7.7 Hz, 4H); ^13^C NMR (150 MHz, DMSO-*d*_6_): *δ* 165.9, 160.9, 150.6, 147.2, 141.9, 139.4, 135.2, 129.5, 129.2, 128.6, 128.0, 126.7, 125.3, 123.0, 115.2, 114.7, 112.2, 69.7, 69.1, 52.1, 46.2, 42.9, 42.8, 32.3; ESIMS(+) m/z 973.0 [M + H]^+^.

*Methyl 4-({3-[2-(4-{5-[4-(2-{1-[(4-methoxycarbonylphenyl)methyl]-2,4-dioxoquinazolin-3-yl}ethyl)phenylamino]pentylamino}phenyl)ethyl]-2,4-dioxoquinazolin-1-yl}methyl)benzoate (5c)*. Following general Procedure B, a mixture of **2b** (1 g, 2 mmol), Cs_2_CO_3_ (1.98 g, 6 mmol), cadaverine (124 mg, 1.2 mmol), μ-OMs dimer (55 mg, 0.05 mmol), and BrettPhos(54 mg, 0.1 mol), and dioxane (20 ml) was stirred under reflux for 16 h. The crude mixture was purified by MPLC (EtOAc/*n-*Heptane = 60%) to provide the title compound as a pale-yellow solid (535 mg, 57%). mp = 149–151 °C; ^1^H NMR (600 MHz, DMSO-*d*_6_): *δ* 8.08 (dd, *J* = 8.2, 1.4 Hz, 2H), 7.91 (d, *J* = 8.3 Hz, 4H), 7.65–7.61 (m, 2H), 7.34 (d, *J* = 8.3 Hz, 4H), 7.26 (t, *J* = 7.6 Hz, 2H), 7.19 (d, *J* = 8.6 Hz, 2H), 6.92 (d, *J* = 8.4 Hz, 4H), 6.47 (d, *J* = 8.4 Hz, 4H), 5.46–5.37 (m, 6H), 4.15 (t, *J* = 7.7 Hz, 4H), 3.82 (s, 6H), 2.96 (q, *J* = 6.4 Hz, 4H), 2.78 (t, *J* = 7.5 Hz, 4H), 1.60–1.52 (m, 4H), 1.48–1.39 (m, 2H); ^13^C NMR (150 MHz, DMSO-*d*_6_): *δ* 165.9, 160.9, 150.6, 147.6, 141.9, 139.4, 135.2, 129.6, 129.2, 128.6, 128.0, 126.7, 124.8, 123.0, 115.2, 114.7, 112.0, 52.1, 46.2, 43.0, 42.9, 32.3, 28.6, 24.4; ESIMS(+) m/z 927.0 [M + H]^+^.

*4-({3-[2-(4-{2-[2–(2-{2-[4-(2-{1-[(4-Hydroxamophenyl)methyl]-2,4-dioxoquinazolin-3-yl}ethyl)phenylamino]ethoxy}ethoxy)ethoxy]ethylamino}phenyl)ethyl]-2,4-dioxoquinazolin-1-yl}methyl)benzohydroxamic acid (6a)*. By following general Procedure C, **5a** (200 mg, 0.2 mmol) was converted into **6a** as a pink solid (54 mg, 27%). mp > 250 °C; ^1^H NMR (600 MHz, DMSO-*d*_6_): *δ* 8.05 (dd, *J* = 8.2, 1.5 Hz, 2H), 7.84 (d, *J* = 8.1 Hz, 4H), 7.65–7.60 (m, 2H), 7.27–7.21 (m, 4H), 7.17 (d, *J* = 8.1 Hz, 4H), 6.95 (d, *J* = 8.4 Hz, 4H), 6.53 (d, *J* = 8.4 Hz, 4H), 5.36 (s, 4H), 4.13 (t, *J* = 8.0 Hz, 4H), 3.62–3.49 (m, 16H), 2.76 (t, *J* = 7.7 Hz, 4H); ^13^C NMR (150 MHz, DMSO-*d*_6_): *δ* 170.6, 161.0, 150.8, 147.4, 139.7, 138.8, 137.2, 135.3, 129.7, 129.3, 128.1, 125.5, 123.0, 115.2, 115.1, 112.3, 69.9, 69.8, 69.2, 48.7, 46.3, 43.1, 42.9; HRMS (ESI): calcd for [C_56_H_59_O_11_N_8_] (M + H)^+^, 1019.4298; found, 1019.4292. HPLC 96.6% (t_R_ = 12.76).

*4-{[3-(2-{4-[2–(2-{2-[4-(2-{1-[(4-Hydroxamophenyl)methyl]-2,4-dioxoquinazolin-3-yl}ethyl)phenylamino]ethoxy}ethoxy)ethylamino]phenyl}ethyl)-2,4-dioxoquinazolin-1-yl]methyl}benzohydroxamic acid (6b)*. By following general Procedure C, **5b** (200 mg, 0.21 mmol) was converted into **6b** as a pink solid (20 mg, 10%). mp = 180–182 °C; ^1^H NMR (600 MHz, DMSO-*d*_6_): *δ* 8.06 (d, *J* = 7.5 Hz, 2H), 7.88 (d, *J* = 8.6 Hz, 2H), 7.69 (d, *J* = 8.0 Hz, 2H), 7.63 (t, *J* = 7.0 Hz, 2H), 7.34–7.17 (m, 8H), 6.94 (d, *J* = 7.5 Hz, 4H), 6.52 (d, *J* = 7.0 Hz, 4H), 7.45–7.32 (m, 4H), 4.19–4.08 (m, 4H), 3.59–3.50 (m, 8H), 3.18–3.11 (m, 4H), 2.82–2.72 (m, 4H); ^13^C NMR (150 MHz, DMSO-*d*_6_): *δ* 160.9, 150.6, 147.2, 139.5, 135.2, 129.7, 129.2, 128.0, 127.3, 126.4, 125.4, 123.0, 115.2, 114.8, 112.2, 69.7, 69.1, 46.2, 42.9, 42.8, 32.3; ESIMS(+) m/z 960.39 [M + N]^+^. HPLC 95.3% (t_R_ = 9.50).

*4-({3-[2-(4-{5-[4-(2-{1-[(4-Hydroxamophenyl)methyl]-2,4-dioxoquinazolin-3-yl}ethyl)phenylamino]pentylamino}phenyl)ethyl]-2,4-dioxoquinazolin-1-yl}methyl)benzohydroxamic acid (6c).* By following general Procedure C, **5c** (200 mg, 0.22 mmol) was converted into **6c** as a pink solid (54 mg, 27%). mp > 250 °C; ^1^H NMR (600 MHz, DMSO-*d*_6_): *δ* 8.06 (dd, *J* = 8.3, 1.6 Hz, 2H), 7.80 (d, *J* = 8.2 Hz, 4H), 7.66–7.61 (m, 2H), 7.27–7.22 (m, 4H), 7.14 (d, *J* = 8.2 Hz, 4H), 6.94 (d, *J* = 8.6 Hz, 4H), 6.49 (d, *J* = 8.2 Hz, 4H), 5.44 (t, *J* = 5.8 Hz, 2H), 5.35 (s, 4H), 4.14 (t, *J* = 8.2 Hz, 4H), 2.96 (q, *J* = 7.0 Hz, 4H), 2.77 (t, *J* = 7.8 Hz, 4H), 1.59–1.52 (m, 4H), 1.48–1.40 (m, 2H); ^13^C NMR (150 MHz, DMSO-*d*_6_): *δ* 169.5, 160.9, 150.6, 147.6, 139.7, 139.6, 136.4, 135.1, 129.4, 129.1, 127.9, 125.2, 124.8, 122.8, 115.1, 115.0, 112.0, 46.2, 43.0, 42.9, 32.4, 28.6, 24.4; ESIMS(+) m/z 960.39 [M + NHOH]^+^. HPLC 95.0% (t_R_ = 10.11).

#### General Procedure D for ring-closed quinazolin2,4-dione dimer synthesis

Isatoic anhydride (1 equiv.) was stirred in EtOAc (1 M) at room temperature for 10 min. To the solution was added diamine linker (0.55 equiv.) and kept stirring for 1 h. A solution of 1,1′-Carbonyldiimidazole (2 equiv.) dissolved in EtOAc (1 M) was added dropwise into the reaction mixture then allowed to stir for 19 h. The solids were filtered, dried *in vacuo*, then mixed with K_2_CO_3_ (6 equiv.) and methyl 4-(bromomethyl)benzoate (2.1 equiv.) in DMF (0.5 M). After stirring at room temperature for 3 h, the reaction mixture was poured into excess ice water. The precipitate formed was filtered then dried *in vacuo*. The resulting solid was triturated with MeOH under sonication to give the desired product.

*Methyl 4-{[3-(11-{1-[(4-methoxycarbonylphenyl)methyl]-2,4-dioxoquinazolin-3-yl}-3,6,9-trioxaundecyl)-2,4-dioxoquinazolin-1-yl]methyl}benzoate (7a)*. Following general Procedure D, isatoic anhydride (1 g, 6.13 mmol) and 1,11-Diamino-3,6,9-trioxaundecane (648.2 mg, 3.37 mmol) were reacted to give the product as a white solid (1.141 g, 48%). mp = 164–166 °C; ^1^H NMR (600 MHz, DMSO-*d*_6_): *δ* 8.06 (d, *J* = 7.6 Hz, 2H), 7.89 (d, *J* = 8.2 Hz, 4H), 7.63 (t, *J* = 7.6 Hz, 2H), 7.42 (d, *J* = 8.1 Hz, 4H), 7.25 (t, *J* = 7.4 Hz, 2H), 7.22 (d, *J* = 8.5 Hz, 2H), 5.44 (s, 4H), 4.17 (t, *J* = 6.2 Hz, 4H), 3.81 (s, 6H), 3.62 (t, *J* = 6.3 Hz, 4H), 3.50–3.39 (m, 8H); ^13^C NMR (150 MHz, DMSO-*d*_6_): *δ* 165.9, 161.0, 150.8, 141.9, 139.5, 135.3, 129.5, 128.6, 128.1, 126.7, 123.0, 115.1, 114.8, 69.7, 69.5, 66.7, 52.1, 46.2, 40.3; ESIMS(+) m/z 779.0 [M + H]^+^.

*Methyl 4-[(3-{2-[2–(2-{1-[(4-methoxycarbonylphenyl)methyl]-2,4-dioxoquinazolin-3-yl}ethoxy)ethoxy]ethyl}-2,4-dioxoquinazolin-1-yl)methyl]benzoate (7b)*. Following general Procedure D, isatoic anhydride (1 g, 6.13 mmol) and 2-(2-(2-aminoethoxy)ethoxy)ethanamine (500 mg, 3.37 mmol) were reacted to give the product as a white solid (1.031 g, 46%). mp = 196–198 °C; ^1^H NMR (600 MHz, DMSO-*d*_6_): *δ* 8.06 (dd, *J* = 7.7, 1.5 Hz, 2H), 7.90 (d, *J* = 8.3 Hz, 4H), 7.65–7.60 (m, 2H), 7.42 (d, *J* = 8.2 Hz, 4H), 7.24 (t, *J* = 7.5 Hz, 2H), 7.21 (d, *J* = 8.5 Hz, 2H), 5.43 (s, 4H), 4.15 (t, *J* = 6.2 Hz, 4H), 3.83–3.79 (m, 4H), 3.62 (t, *J* = 6.3 Hz, 4H); ^13^C NMR (150 MHz, DMSO-*d*_6_): *δ* 165.9, 161.0, 150.7, 141.9, 139.5, 135.3, 135.0, 129.5, 128.6, 128.1, 126.7, 123.0, 122.5, 115.1, 114.7, 69.5, 66.7, 52.1, 46.2, 40.2; ESIMS(+) m/z 735.0 [M + H]^+^.

*Methyl 4-{[3-(5-{1-[(4-methoxycarbonylphenyl)methyl]-2,4-dioxoquinazolin-3-yl}pentyl)-2,4-dioxoquinazolin-1-yl]methyl}benzoate (7c)*. Following general Procedure D, isatoic anhydride (1 g, 6.13 mmol) and cadaverine (344.78 mg, 3.37 mmol) were reacted to give the product as a white solid (0.761 g, 36%). mp = 124–126 °C; ^1^H NMR (600 MHz, DMSO-*d*_6_): *δ* 8.01 (d, *J* = 8.3 Hz, 2H), 7.88 (d, *J* = 8.3 Hz, 4H), 7.63 (t, *J* = 7.6 Hz, 2H), 7.41 (d, *J* = 8.3 Hz, 4H), 7.24 (t, *J* = 7.6 Hz, 2H), 7.20 (d, *J* = 9.0 Hz, 2H), 5.40 (s, 4H), 4.01 (t, *J* = 6.9 Hz, 4H), 3.81 (s, 6H), 1.75–1.65 (m, 4H), 1.42–1.33 (m, 2H); ^13^C NMR (150 MHz, DMSO-*d*_6_): *δ* 165.9, 161.1, 150.8, 150.1, 142.0, 139.5, 135.2, 129.5, 128.6, 128.1, 126.7, 123.0, 122.4, 115.2, 114.7, 52.1, 46.2, 41.1, 27.1, 23.8; ESIMS(+) m/z 689.0 [M + H]^+^.

*4-{[3-(11-{1-[(4-Hydroxamophenyl)methyl]-2,4-dioxoquinazolin-3-yl}-3,6,9-trioxaundecyl)-2,4-dioxoquinazolin-1-yl]methyl}benzohydroxamic acid (8a)*. By following general Procedure C, **7a** (300 mg, 0.39 mmol) was converted into **8a** as a pink solid (129 mg, 43%). mp = 115–117 °C; ^1^H NMR (600 MHz, DMSO-*d*_6_): *δ* 8.06 (d, *J* = 7.6 Hz, 2H), 7.68 (d, *J* = 8.1 Hz, 4H), 7.63 (t, *J* = 7.6 Hz, 2H), 7.34 (d, *J* = 8.1 Hz, 4H), 7.28–7.20 (m, 4H), 5.40 (s, 4H), 4.18 (t, *J* = 6.2 Hz, 4H), 3.62 (t, *J* = 6.4 Hz, 4H), 3.51–3.41 (m, 8H); ^13^C NMR (150 MHz, DMSO-*d*_6_): *δ* 163.8, 161.1, 150.8, 139.5, 139.1, 135.3, 132.3, 128.1, 127.2, 126.4, 123.0, 115.1, 114.9, 69.7, 69.5, 66.7, 46.1, 40.3; HRMS (ESI): calcd for [C_40_H_41_O_11_N_6_] (M + H)^+^, 781.2828; found, 781.2837. HPLC 97.0% (t_R_ = 8.58).

*4-[(3-{2-[2-(2-{1-[(4-Hydroxamophenyl)methyl]-2,4-dioxoquinazolin-3-yl}ethoxy)ethoxy]ethyl}-2,4-dioxoquinazolin-1-yl)methyl]benzohydroxamic acid (8b)*. By following general Procedure C, **7b** (200 mg, 0.27 mmol) was converted into **8b** as a pink solid (54 mg, 27%). mp = 164–166 °C; ^1^H NMR (600 MHz, DMSO-*d*_6_): *δ* 8.11–8.01 (m, 2H), 7.76–7.57 (m, 6H), 7.43–7.15 (m, 8H), 5.40 (s, 4H), 4.16 (s, 4H), 3.70–3.49 (m, 8H); ^13^C NMR (150 MHz, DMSO-*d*_6_): *δ* 163.8, 161.0, 150.7, 139.5, 139.4, 135.2, 132.0, 128.1, 127.2, 126.4, 123.0, 115.1, 114.8, 69.5, 66.7, 46.1, 40.3; HRMS (ESI): calcd for [C_38_H_37_O_10_N_6_] (M + H)^+^, 737.2566; found, 737.2576. HPLC 95.4% (t_R_ = 8.54).

*4-{[3-(5-{1-[(4-Hydroxamophenyl)methyl]-2,4-dioxoquinazolin-3-yl}pentyl)-2,4-dioxoquinazolin-1-yl]methyl}benzohydroxamic acid (8c)*. By following general Procedure C, **7c** (200 mg, 0.29 mmol) was converted into **8c** as a pink solid (50 mg, 25%). mp = 164–166 °C; ^1^H NMR (600 MHz, DMSO-*d*_6_): *δ* 8.10–7.91 (m, 2H), 7.80–7.53 (m, 6H), 7.46–7.04 (m, 8H), 5.38 (s, 4H), 4.15–3.87 (m, 4H), 1.83–1.59 (m, 4H), 1.49–1.32 (m, 2H); ^13^C NMR (150 MHz, DMSO-*d*_6_): *δ* 163.8, 161.1, 150.8, 139.5, 139.3, 135.1, 132.1, 138.0, 127.2, 126.3, 122.9, 115.2, 114.8, 46.1, 41.1, 27.1, 23.9; HRMS (ESI): calcd for [C_37_H_35_O_8_N_6_] (M + H)^+^, 691.2511; found, 691.2514. HPLC 96.4% (t_R_ = 10.70).

*Methyl 4-[(2,4-dioxo-3,1-benzoxazinan-1-yl)methyl]benzoate (9)*. To a solution of isatoic anhydride (20 g, 122.6 mmol), methyl 4-(bromomethyl)benzoate (29.49 g, 128.73 mmol) in DMF (400 ml) was added K_2_CO_3_ (18.64 g, 134.86 mmol) and stirred at room temperature for 2 h. The reaction mixture was poured into excess H_2_O. The precipitate formed was filtered then dried *in vacuo*. The resulting solid was triturated with MeOH under sonication to give the product as a white solid (19.6 g, 51%). mp = 187–189 °C; ^1^H NMR (600 MHz, DMSO-*d*_6_): *δ* 8.05 (d, *J* = 7.1 Hz, 1H), 7.92 (d, *J* = 7.1 Hz, 2H), 7.72 (t, *J* = 8.0 Hz, 1H), 7.56 (d, *J* = 7.1 Hz, 2H), 7.31 (t, *J* = 7.1 Hz, 1H), 7.19 (d, *J* = 8.9 Hz, 1H), 5.37 (s, 2H), 3.83 (s, 3H); ^13^C NMR (150 MHz, DMSO-*d*_6_): *δ* 166.0, 158.8, 148.3, 145.5, 141.2, 141.0, 137.0, 129.6, 129.5, 128.8, 127.2, 126.9, 123.8, 115.0, 112.2, 52.1, 47.5; ESIMS(+) m/z 312.0 [M + H]^+^.

#### General Procedure E for 2-amino benzamide dimer synthesis

To a round bottom flask was added **9** (1 equiv.) and EtOAc (1 M). After stirring at room temperature for 10 min, diamine linker (0.5 equiv.) was introduced then stirred at room temperature for 4 h. For less reactive reactants, **9** (1 equiv.) and diamine linker (0.5 equiv.) was dissolved in DMAC (1 M) then refluxed under 250 W MW irradiation for 12 min. Upon completion for either way, the reaction mixture was partitioned between CH_2_Cl_2_ and H_2_O. The aqueous layer was extracted extra two times with CH_2_Cl_2_. Combined organic layer was dried over MgSO_4_, filtered, and dried *in vacuo* to obtain the desired product.

*Methyl 4-({2-[N-11-(2-{[(4-methoxycarbonylphenyl)methyl]amino}benzylamino)-3,6,9-trioxaundecylcarbamoyl]phenylamino}methyl)benzoate (10a)*. Following general Procedure E, **9** (1 g, 3.21 mmol) and 1,11-Diamino-3,6,9-trioxaundecane (308.83 mg, 1.61 mmol) were reacted to give the product as a yellow oil (1.914 g, 82%).^1^H NMR (600 MHz, DMSO-*d*_6_): *δ* 8.36 (s, 4H), 7.92 (d, *J* = 8.0 Hz, 4H), 7.60–7.42 (m, 6H), 7.20–7.15 (m, 2H), 7.60–7.50 (m, 4H), 4.47 (s, 4H), 3.82 (s, 6H), 3.56–3.37 (m, 16H); ^13^C NMR (150 MHz, DMSO-*d*_6_): *δ* 169.4, 166.2, 148.9, 145.7, 132.3, 129.5, 128.5, 128.3, 127.2, 115.5, 114.8, 111.6, 69.9, 69.7, 69.0, 52.0, 45.9, 39.0; ESIMS(+) m/z 727.0 [M + H]^+^.

*Methyl 4-{[2-(N-2-{2-[2-(2-{[(4-methoxycarbonylphenyl)methyl]amino}benzylamino)ethoxy]ethoxy}ethylcarbamoyl)phenylamino]methyl}benzoate (10b)*. Following general Procedure E, **9** (1 g, 3.21 mmol) and 2-(2-(2-aminoethoxy)ethoxy)ethanamine (237.88 mg, 1.61 mmol) were reacted to give the product as a yellow oil (2.045 g, 93%). ^1^H NMR (600 MHz, DMSO-*d*_6_): *δ* 8.41–8.32 (m, 4H), 7.92 (d, *J* = 6.6 Hz, 4H), 7.62–7.42 (m, 6H), 7.22–7.13 (m, 2H), 6.63–6.50 (m, 4H), 4.47 (s, 4H), 3.82 (s, 6H), 3.64–3.36 (m, 12H); ^13^C NMR (150 MHz, DMSO-*d*_6_): *δ* 169.3, 166.1, 148.8, 145.7, 132.2, 129.4, 128.4, 128.2, 127.2, 115.4, 114.7, 115.2, 69.6, 68.9, 52.0, 45.8; ESIMS(+) m/z 683.0 [M + H]^+^.

*Methyl 4-({2-[N-5-(2-{[(4-methoxycarbonylphenyl)methyl]amino}benzylamino)pentylcarbamoyl]phenylamino}methyl)benzoate (10c)*. Following general Procedure E, **9** (1 g, 3.21 mmol) and cadaverine (164 mg, 1.61 mmol) were reacted to give the product as a white solid (2.259 g, 64%). mp = 154–156 °C; ^1^H NMR (600 MHz, DMSO-*d*_6_): *δ* 8.37–8.27 (m, 4H), 7.91 (d, *J* = 7.1 Hz, 4H), 7.58–7.43 (m, 6H), 7.20–7.14 (m, 2H), 6.58–6.50 (m, 4H), 4.47 (s, 4H), 3.83 (s, 6H), 3.28–3.20 (m, 4H), 1.62–1.52 (m, 4H), 1.4.2–1.32 (m, 2H); ^13^C NMR (150 MHz, DMSO-*d*_6_): *δ* 169.0, 166.1, 148.7, 145.7, 132.0, 129.4, 128.3, 128.2, 127.2, 115.8, 114.6, 111.4, 52.0, 45.8, 38.8, 28.8, 24.0; ESIMS(+) m/z 637.0 [M + H]^+^.

*N-[11-(2-{[(4-hydroxamophenyl)methyl]amino}benzylamino)-3,6,9-trioxaundecyl]-2-{[(4-hydroxamophenyl)methyl]amino}benzamide (11a)*. By following general Procedure C, **10a** (200 mg, 0.28 mmol) was converted into **11a** as a pink solid (78 mg, 39%). mp = 99–101 °C; ^1^H NMR (600 MHz, DMSO-*d*_6_): *δ* 11.17 (s, 2H), 9.02 (s, 2H), 8.36 (t, *J* = 5.7 Hz, 2H), 8.30 (t, *J* = 5.7 Hz, 2H), 7.70 (d, *J* = 7.9 Hz, 4H), 7.55 (d, *J* = 7.1 Hz, 2H), 7.39 (d, *J* = 7.9 Hz, 4H), 7.18 (t, *J* = 7.9 Hz, 2H), 6.61–6.50 (m, 4H), 4.42 (d, *J* = 5.7 Hz, 4H), 3.58–3.35 (m, 16H); ^13^C NMR (150 MHz, DMSO-*d*_6_): *δ* 169.2, 164.1, 148.8, 143.1, 132.2, 131.6, 131.4, 129.5, 128.4, 127.1, 126.9, 115.3, 114.6, 111.5, 69.7, 69.6, 68.8, 45.7, 38.8; HRMS (ESI): calcd for [C_38_H_45_O_9_N_6_] (M + H)^+^, 729.3243; found, 729.3238. HPLC 99.2% (t_R_ = 10.20).

*N-(2-{2-[2-(2-{[(4-hydroxamophenyl)methyl]amino}benzylamino)ethoxy]ethoxy}ethyl)-2-{[(4-hydroxamophenyl)methyl]amino}benzamide (11b)*. By following general Procedure C, **10b** (200 mg, 0.29 mmol) was converted into **11b** as a pink solid (58 mg, 29%). mp = 144–146 °C; ^1^H NMR (600 MHz, DMSO-*d*_6_): *δ* 11.20 (s, 2H), 9.06 (s, 2H), 8.43–8.32 (m, 2H), 8.32–8.24 (m, 2H), 7.70 (d, *J* = 7.8 Hz, 4H), 7.55 (d, *J* = 7.3 Hz, 2H), 7.39 (d, *J* = 7.8 Hz, 4H), 7.18 (t, *J* = 7.3 Hz, 2H), 6.63–6.49 (m, 4H), 4.42 (d, *J* = 4.9 Hz, 4H), 3.62-3.48 (m, 12H); ^13^C NMR (150 MHz, DMSO-*d*_6_): *δ* 169.2, 164.1, 148.8, 143.1, 132.2, 131.4, 128.3, 127.1, 126.9, 115.3, 114.5, 111.5, 69.6, 68.9, 45.7, 38.8; HRMS (ESI): calcd for [C_36_H_41_O_8_N_6_] (M + H)^+^, 685.2980; found, 685.2982. HPLC 95.0% (t_R_ = 10.25).

*N-[5-(2-{[(4-hydroxamophenyl)methyl]amino}benzylamino)pentyl]-2-{[(4-hydroxamophenyl)methyl]amino}benzamide (11c)*. By following general Procedure C, **10c** (200 mg, 0.31 mmol) was converted into **11c** as a pink solid (41 mg, 20%). mp = 150–152 °C; ^1^H NMR (600 MHz, DMSO-*d*_6_): *δ* 11.13 (s, 2H), 8.99 (s, 2H), 8.34 (t, *J* = 4.7 Hz, 2H), 8.27 (t, *J* = 6.6 Hz, 2H), 7.70 (d, *J* = 7.6 Hz, 4H), 7.54 (d, *J* = 6.6 Hz, 2H), 7.39 (d, *J* = 7.6 Hz, 4H), 7.17 (t, *J* = 7.6 Hz, 2H), 6.60-6.49 (m, 4H), 4.42 (d, *J* = 5.7 Hz, 4H), 3.23 (q, *J* = 4.7 Hz, 4H), 1.61–1.48 (m, 4H), 1.43–1.30 (m, 2H); ^13^C NMR (150 MHz, DMSO-*d*_6_): *δ* 169.0, 164.1, 148.7, 143.1, 132.0, 131.4, 128.3, 127.0, 126.9, 115.7, 114.5, 111.4, 45.7, 38.8, 28.8, 24.0; HRMS (ESI): calcd for [C_35_H_39_O_6_N_6_] (M + H)^+^, 639.2926; found, 639.2931. HPLC 99.0% (t_R_ = 10.85).

#### HDAC enzyme activity assay

HDAC inhibition assays were performed by Reaction Biology Corporation (Malvern, PA). Full-length recombinant *h*HDAC 6 was expressed in a baculovirus expression system in SF9 cells. Test compounds were dissolved in DMSO at 30 μM and then tested in ten-dose IC_50_ mode with three-fold serial dilutions starting at 10 or 30 μM. The enzyme was diluted in reaction buffer (50 nM Tris-HCl, pH 8.0, 137 mM NaCl, 2.7 mM KCl, 1 mM MgCl_2_, 1 mg/mL BSA, 1% DMSO) and then the test compound and specific substrate were added in sequence. For HDAC 6, a fluorogenic peptide from p53 residues 379–382 (RHKKAc, 50 μM) was used as the substrate. The reaction was stopped after 2 h at 30°C. Trichostatin A was used as an internal control.

#### Calculation of clogP and TPSA values

cLogP and TPSA values were calculated using Molsoft LLC (https://www.molsoft.com).

#### Molecular Docking^28^

The crystal structures of *h*HDAC6 CD2 (PDB ID: 5EDU) were obtained from Protein Data Bank (https://www.rcsb.org/structure/5EDU). Molecular docking was performed using Molecular Operating Environment (MOE) with compound **1** and **11a**^29^. Trichostatin A in *h*HDAC6 CD2 was served as a reference structure. The best binding conformations of compound **1** were selected for further analysis. The figures were generated using MOE (Chemical Computing Group, ULC.)

#### Sulforhodamine B (SRB) assay

The A549 and PC-3 human lung and prostate cancer cell lines were acquired from the American Type Culture Collection (Rockville, MD). The cells were grown in RPMI 1640 medium supplemented with 10% FBS (v/v), penicillin (100 units/ml), and streptomycin (100 µg/ml). The cultures were kept in a 37 °C incubator with 5% CO_2_. When the adherent cultures reached 80% confluence, they were passaged using 0.05% trypsin-EDTA. PC-3 and A549 cells were seeded into 96-well plates in RPMI medium with 5% (v/v) foetal bovine serum for 24 h. Cells in partial wells were fixed with 10% trichloroacetic acid (TCA) to represent the initial time of compound treatment. The cells were treated with or without the indicated compound for 48 h and then were fixed with 10% TCA. After the washout with ddH_2_O and air-dried, SRB at 0.4% (w/v) in 1% acetic acid was added for staining. Unbound SRB was washed with 1% acetic acid and SRB-bounded cells were solubilised with 10 mM Tris base. The absorbance was read at a wavelength of 515 nm for the determination of cell growth.

#### Flow cytometric analysis of DNA content by propidium iodide (PI) staining

After compound treatment, the cells were trypsinised and harvested, and were fixed with 70% (v/v) alcohol at −20 °C for 30 min. The cells were washed with phosphate-buffered saline (PBS) and centrifuged, and then resuspended with the solution containing PI (80 μg/ml), Triton X-100 (0.1%, v/v), and RNase (100 μg/ml). DNA content analysis and cell cycle distribution were examined with FACScan FL2 channel and CellQuest software (Becton Dickinson, Mountain View, CA).

#### Western blotting

After compound treatment, the cells were harvested and centrifuged. The cells were lysed in 80 µl ice-cold lysis buffer for 30 min and the lysates were centrifuged (12000 rpm, 30 min). The supernatants quantified by Bio-Rad protein assay kit (Bio-Rad Laboratories, Hercules, CA) were mixed with sample buffer (0.3 M Tris pH6.8, 10% SDS, 50% glycerol, 10% β-mercaptoethanol, and 0.02% bromophenol blue) and heated at 95 °C for 10 min. The protein (30 µg) was separated by electrophoresis in 8% or 12% SDS-PAGE, transferred to PVDF membranes, and detected with specific antibodies (1:1000 dilution for the first and 1:7000 for the second antibodies). The immunoreactive proteins were detected with an enhanced chemiluminescence detection kit (Amersham, Buckinghamshire, UK) and the images were captured by ChemiDoc^™^ MP System (Bio-Rad Laboratories, Hercules, CA).

## Supplementary Material

JEIMC Manuscript Supporting Information Revised.docx

## Data Availability

The datasets presented in the current study are available from the corresponding author, CWY, upon reasonable request.
