# Supplementary material for: Design and synthesis of novel HDAC6 inhibitor dimer as HDAC6 degrader for cancer treatment by palladium catalysed dimerisation
Source: J Enzyme Inhib Med Chem. 2025 Feb 27;40(1):2468355. doi: 10.1080/14756366.2025.2468355 (PMC11869342; doi:10.1080/14756366.2025.2468355)
Supplement: JEIMC Manuscript Supporting Information Revised.docx [file IENZ_A_2468355_SM8415.docx]

**Supporting Information**

**Design and Synthesis of Novel HDAC6 Inhibitor Dimer as HDAC6 Degrader for Cancer Treatment by Palladium Catalysed Dimerisation**

Ching Lin^a^, Jui-Ling Hsu^a,b,c^, Yu-Tung Hsu^a^, Kuo-Chen Fan^a^, Sian-Siou Wu^a^, Miao-Hsia Lin^d^, Jih-Hwa Guh^a^, Chao-Wu Yu^a,*^

*^a^School of Pharmacy, National Taiwan University, No. 33, Linsen S. Rd., Zhongzheng Dist., Taipei 100, Taiwan*

*^b^Department of Nursing, Chang Gung University of Science and Technology, No. 261, Wenhua 1st Rd., Guishan Dist., Taoyuan City 33303, Taiwan*

*^c^Division of Hematology-Oncology, Department of Internal Medicine, New Taipei Municipal TuCheng Hospital, No. 6, Sec. 2, Jincheng Rd., Tucheng Dist., New Taipei City 236, Taiwan*

*^d^National Taiwan University, Department and Graduate Institute of Medical Microbiology, College of Medicine, Taipei 100, Taiwan*

^*^Corresponding author. Chao-Wu Yu – National Taiwan University, School of Pharmacy, College of Medicine, Taipei 100, Taiwan; orcid.org/0000-0002-5176-4225; Phone: 886-2-3366-8697; Email: stifenyu@ntu.edu.tw

**Contents**

**Figure S1…………………………………………………………………………………….S3**

**^1^H and ^13^C NMR Spectra………………………...………………………….……...….S4-S30**

**HPLC………………………………………………………………………………….S31-S36**


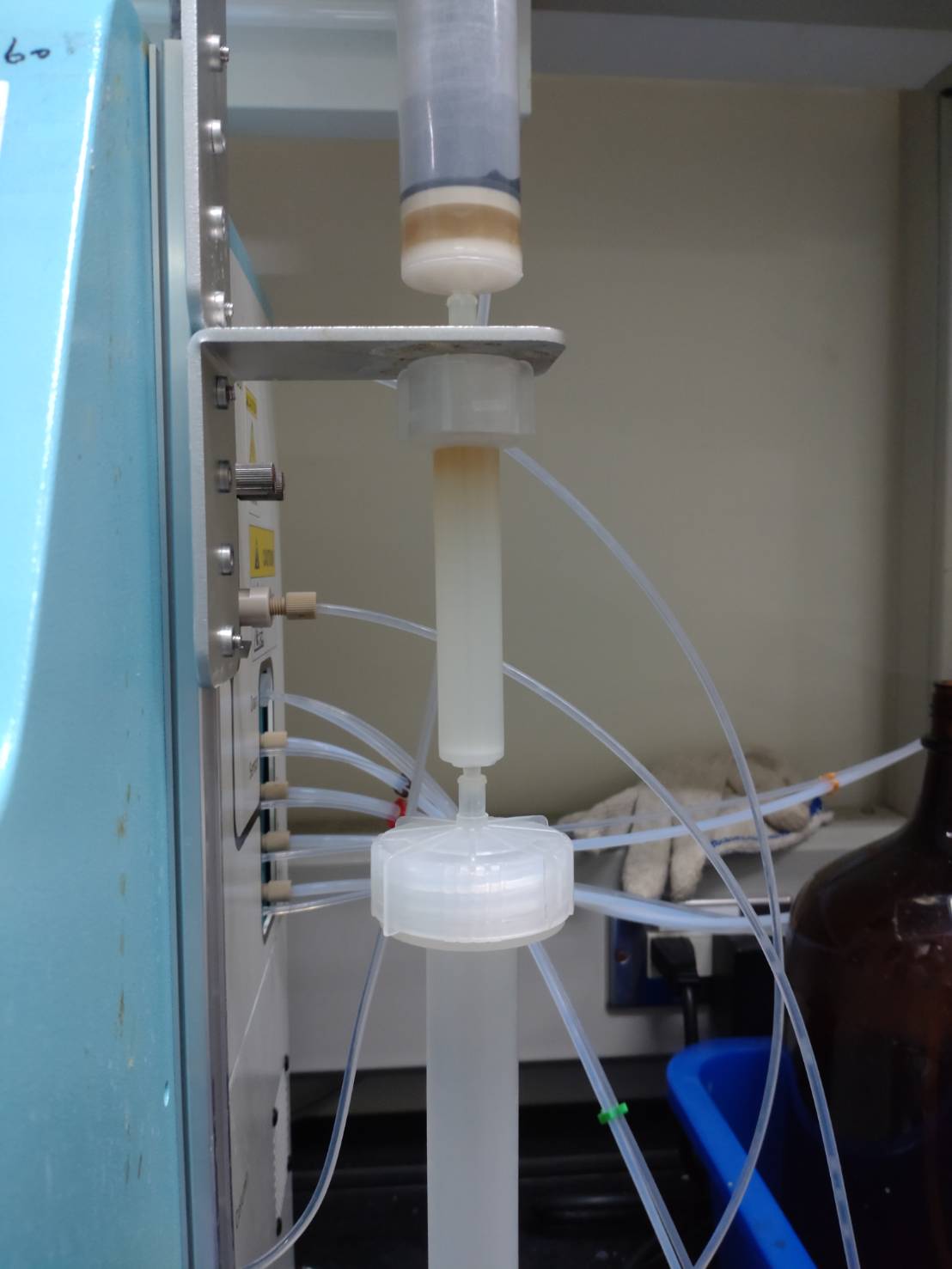

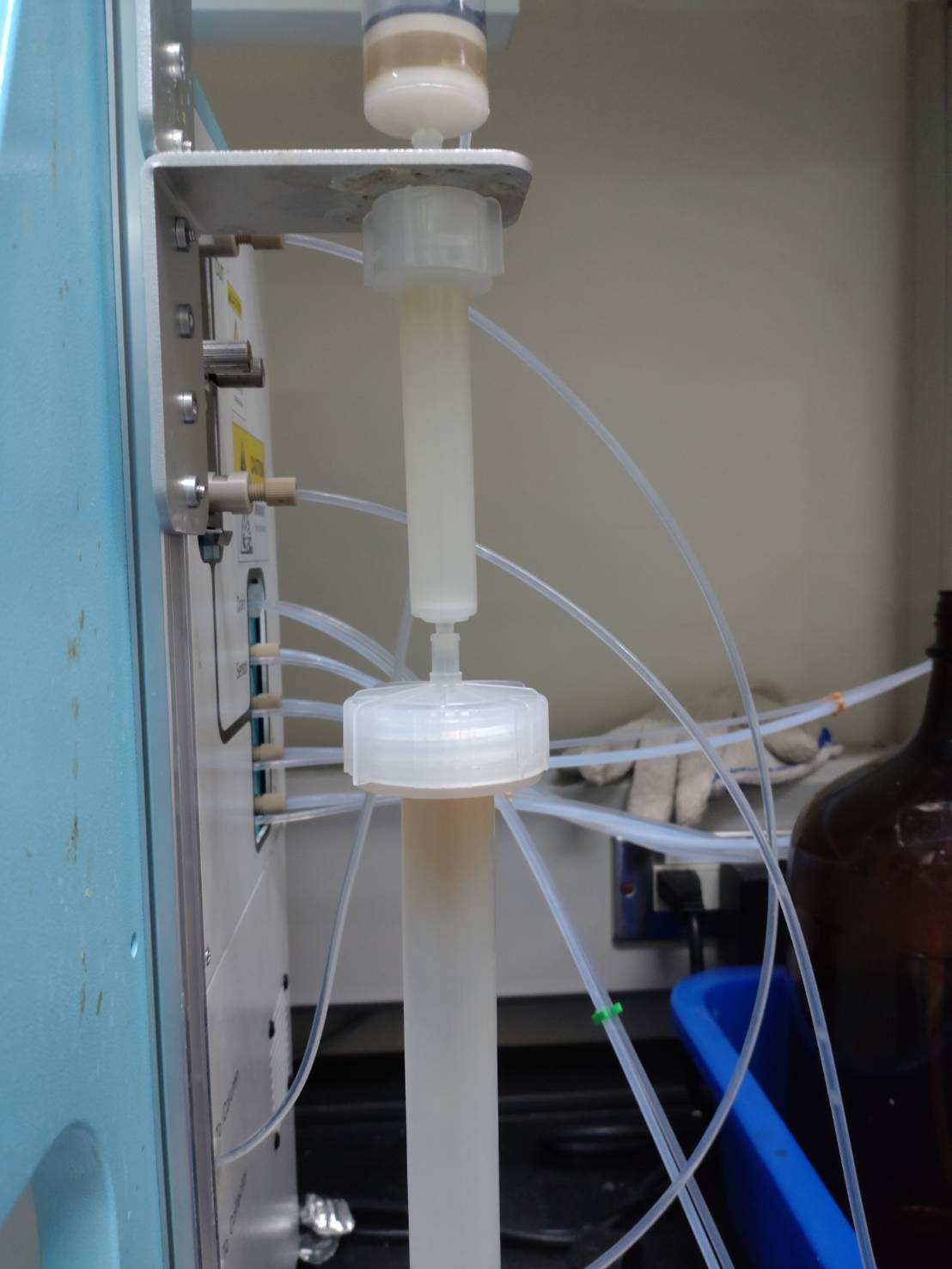


**b**

**a**

Figure S1. Set up for the TNR method. From top to bottom are the sample loading cartridge, NH2 silica gel cartridge, and the normal phase silica gel cartridge, respectively. (a) Washing with MeOH/CH_2_Cl_2_=10%, desired di-hydroxamic acid was trapped in NH2 silica gel cartridge. (b) Eluting with MeOH/CH_2_Cl_2_=0-5% (0.1% TFA), desired di-hydroxamic acid separated in normal phase silica gel cartridge.

^1^H NMR of **2a** (600 MHz, DMSO-*d*_6_)

^^
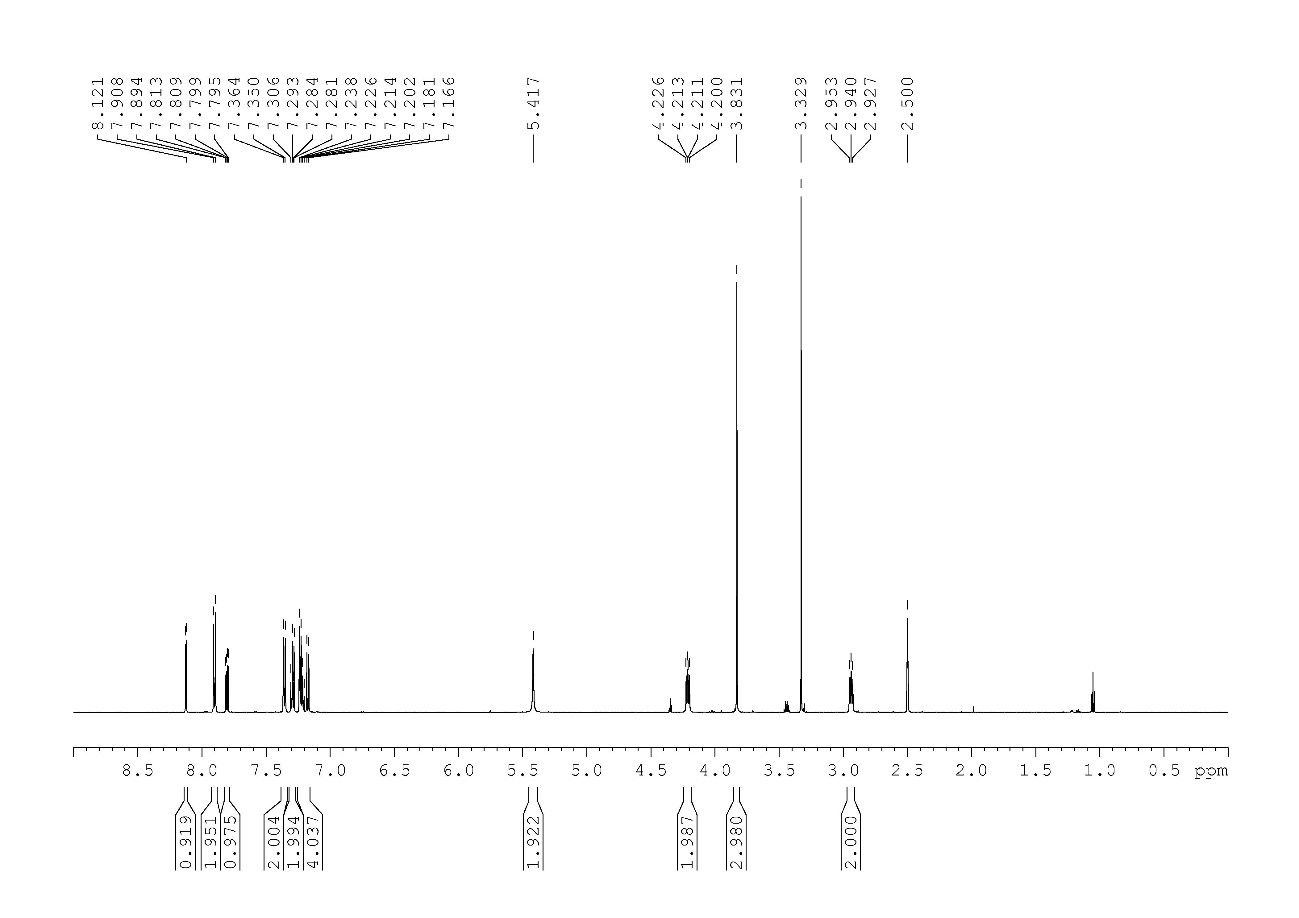


^13^C NMR of **2a** (150 MHz, DMSO-*d*_6_)

^^
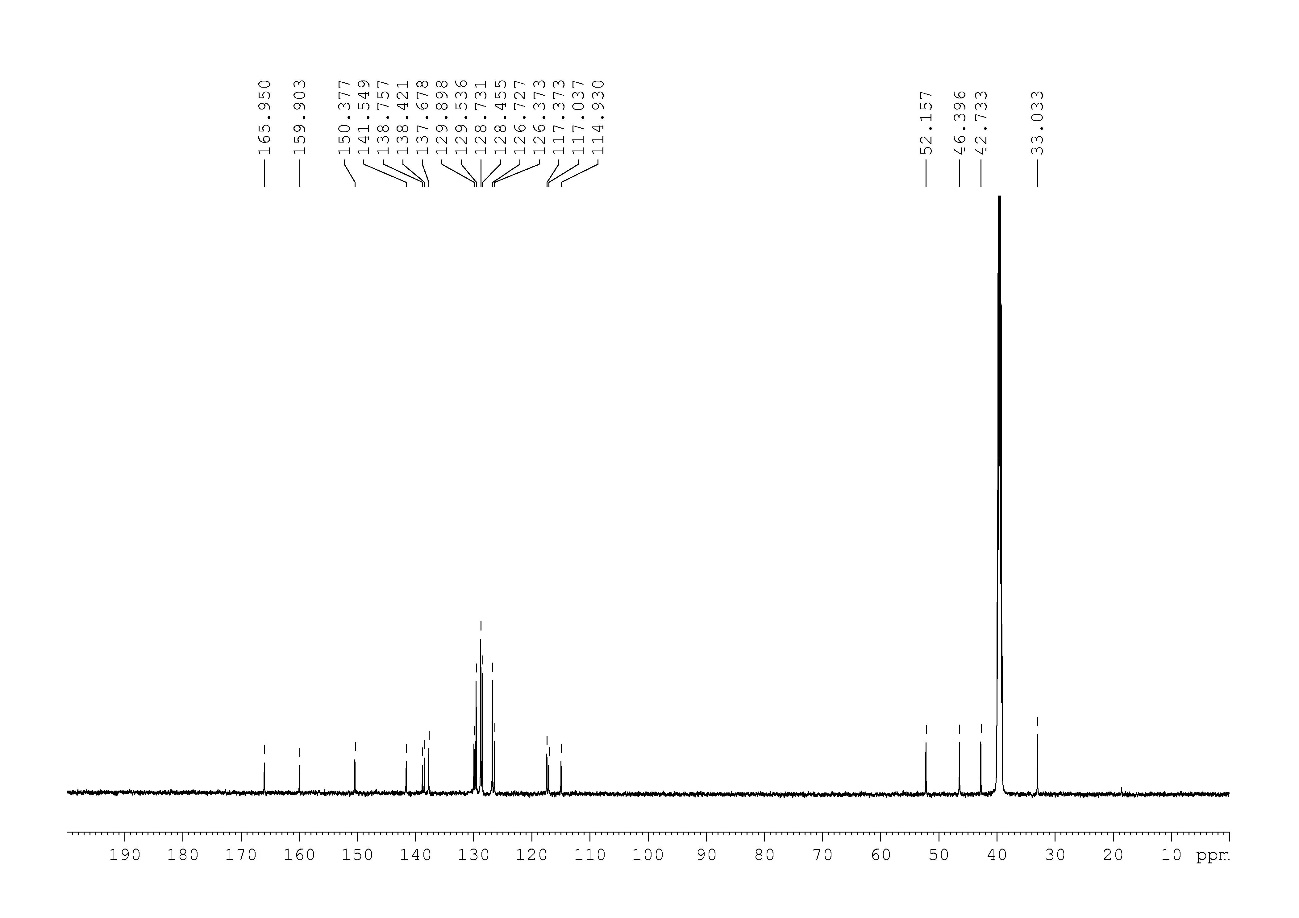


^1^H NMR of **2b** (600 MHz, DMSO-*d*_6_)

^^
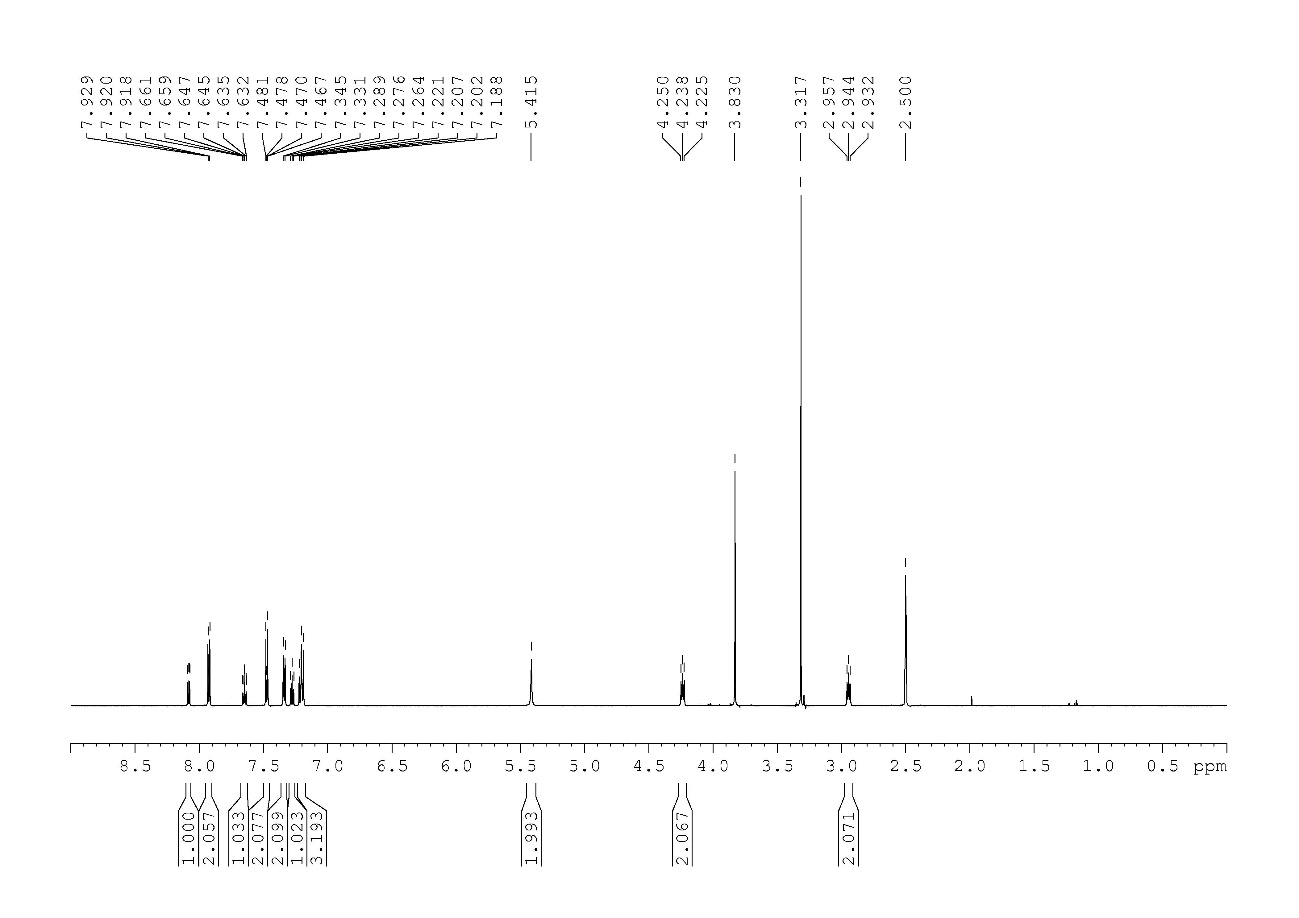


^13^C NMR of **2b** (150 MHz, DMSO-*d*_6_)

^^
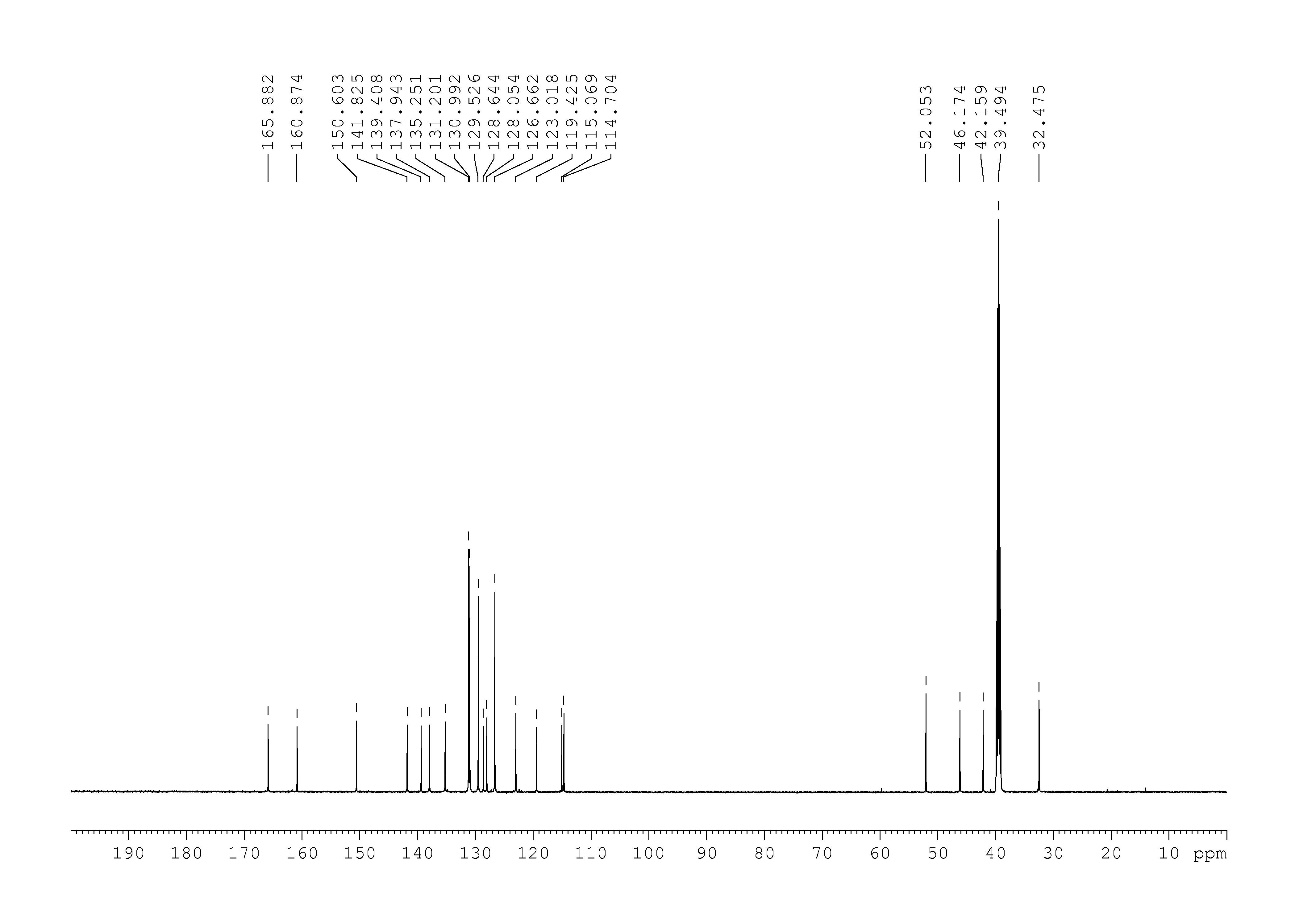


^1^H NMR of **3a** (200 MHz, DMSO-*d*_6_)

^^
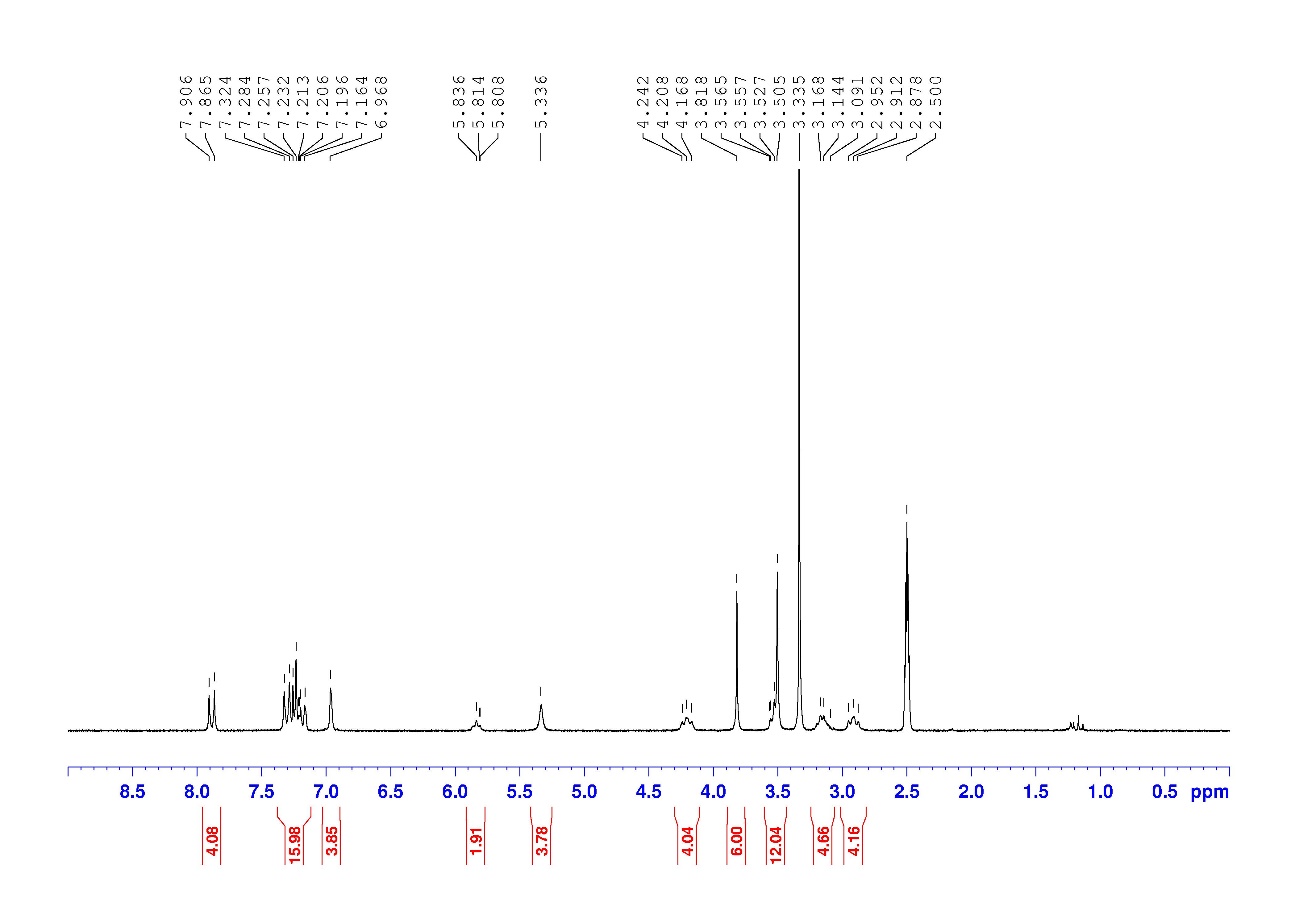


^13^C NMR of **3a** (150 MHz, DMSO-*d*_6_)

^^
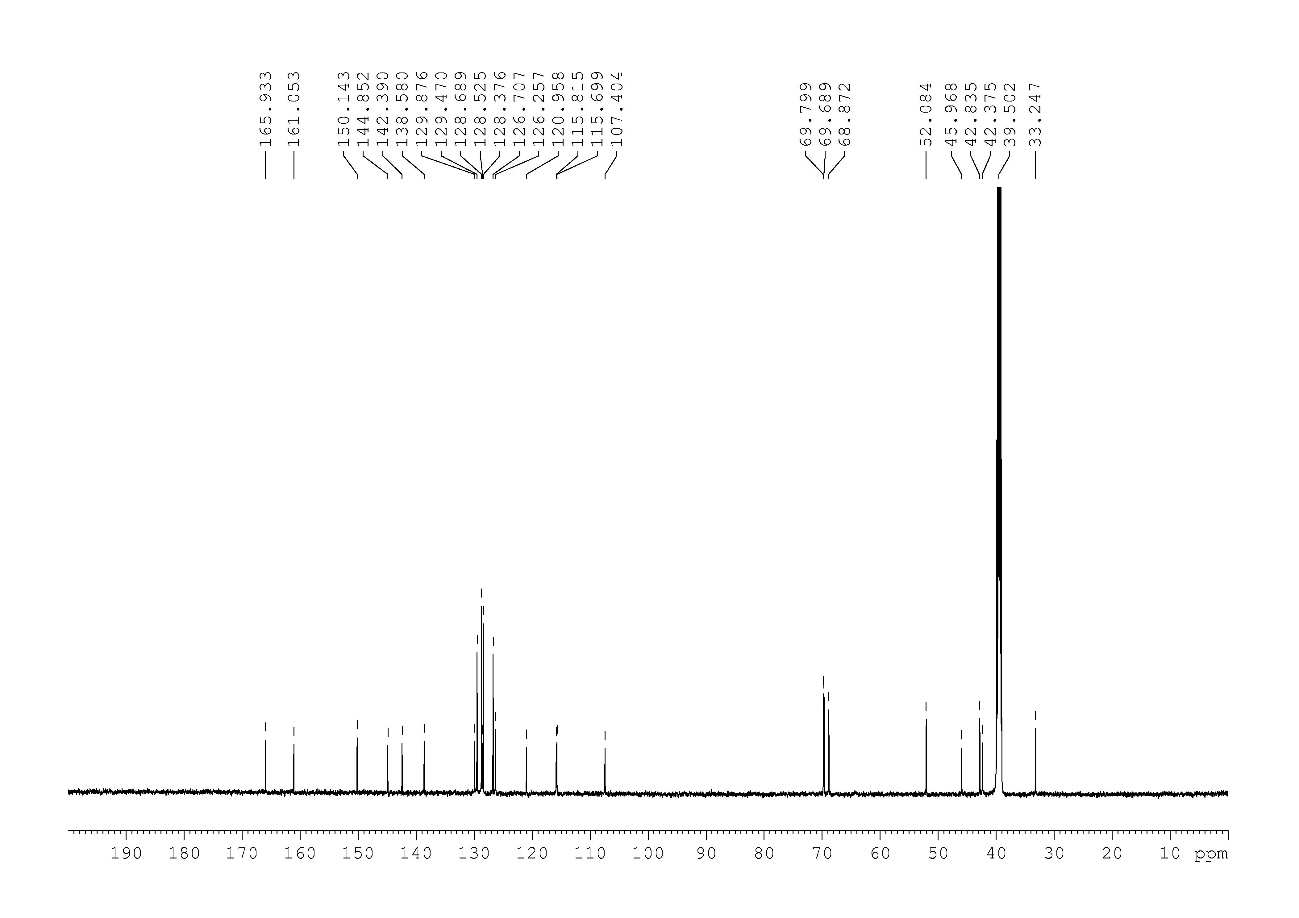


^1^H NMR of **3b** (600 MHz, DMSO-*d*_6_)

^^
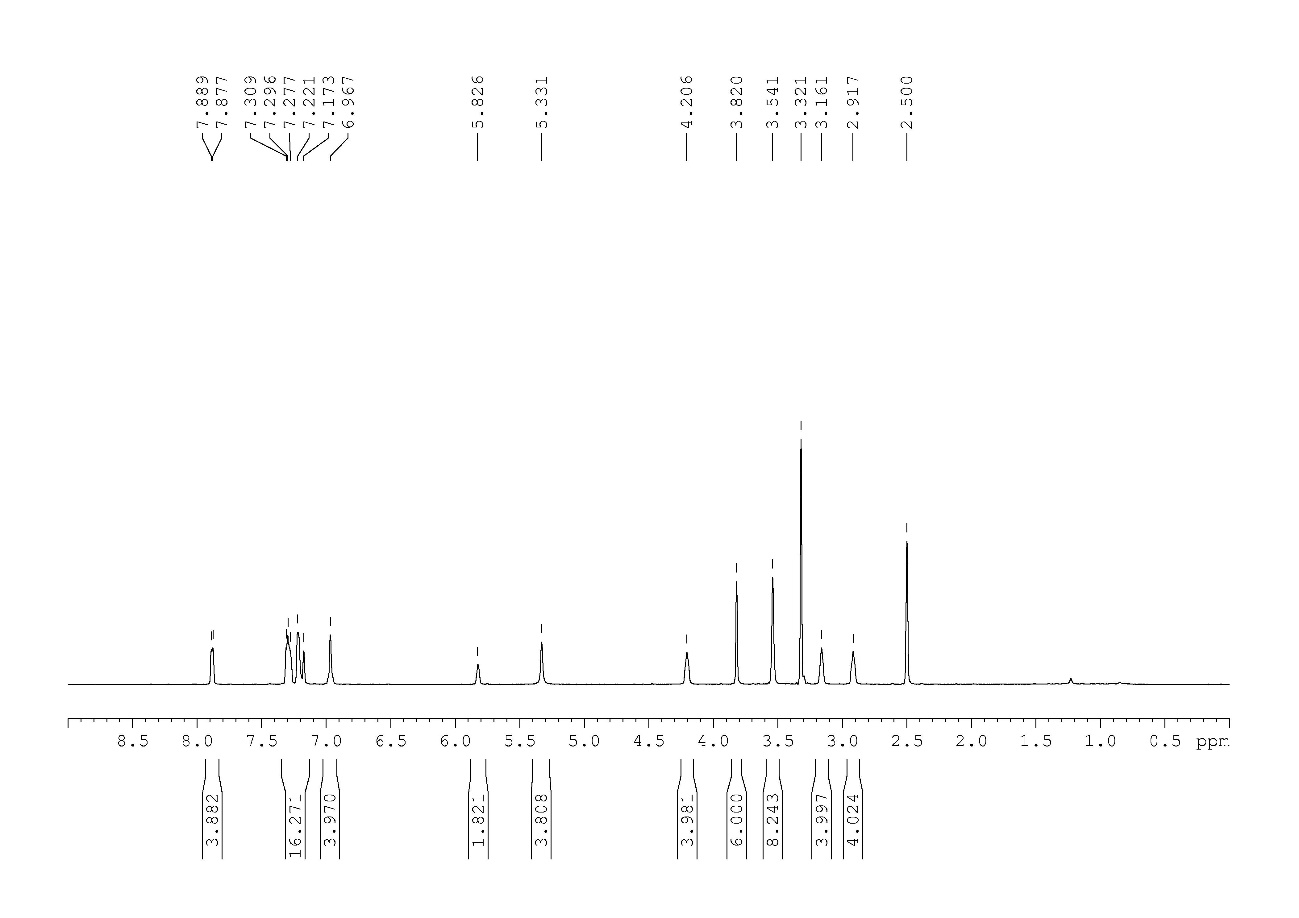


^13^C NMR of **3b** (150 MHz, DMSO-*d*_6_)

^^
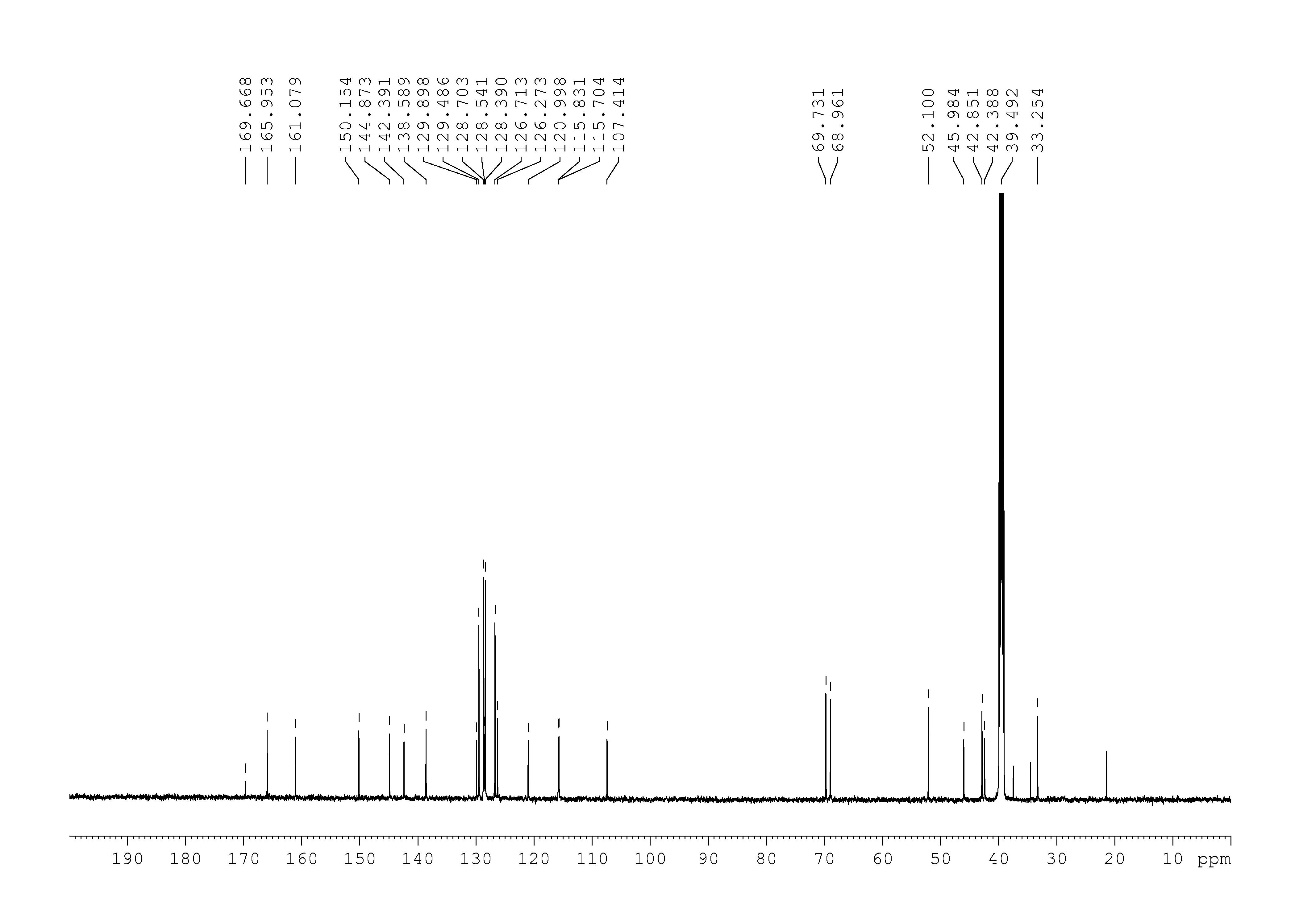


^1^H NMR of **3c** (600 MHz, DMSO-*d*_6_)

^^
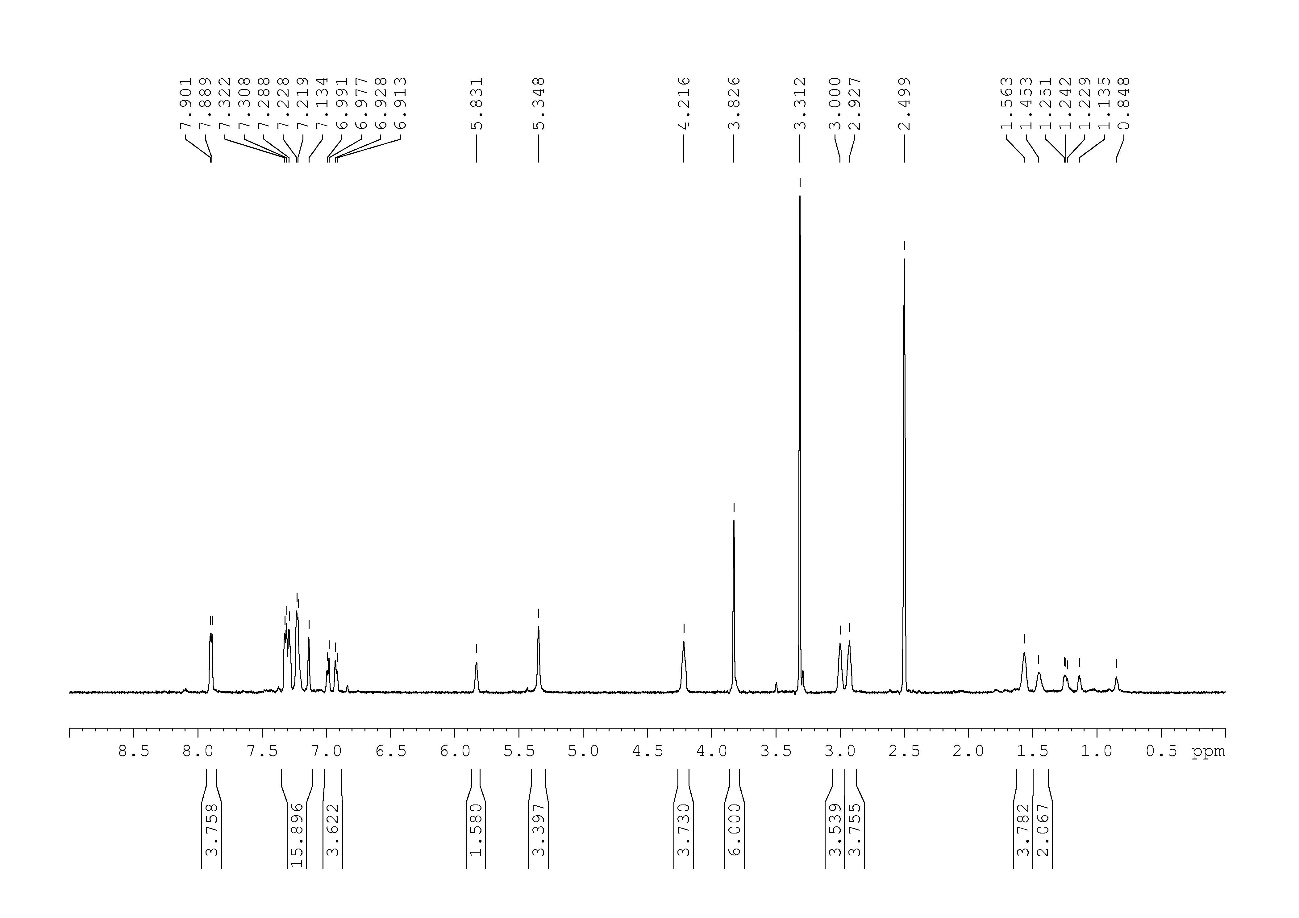


^13^C NMR of **3c** (150 MHz, DMSO-*d*_6_)

^^
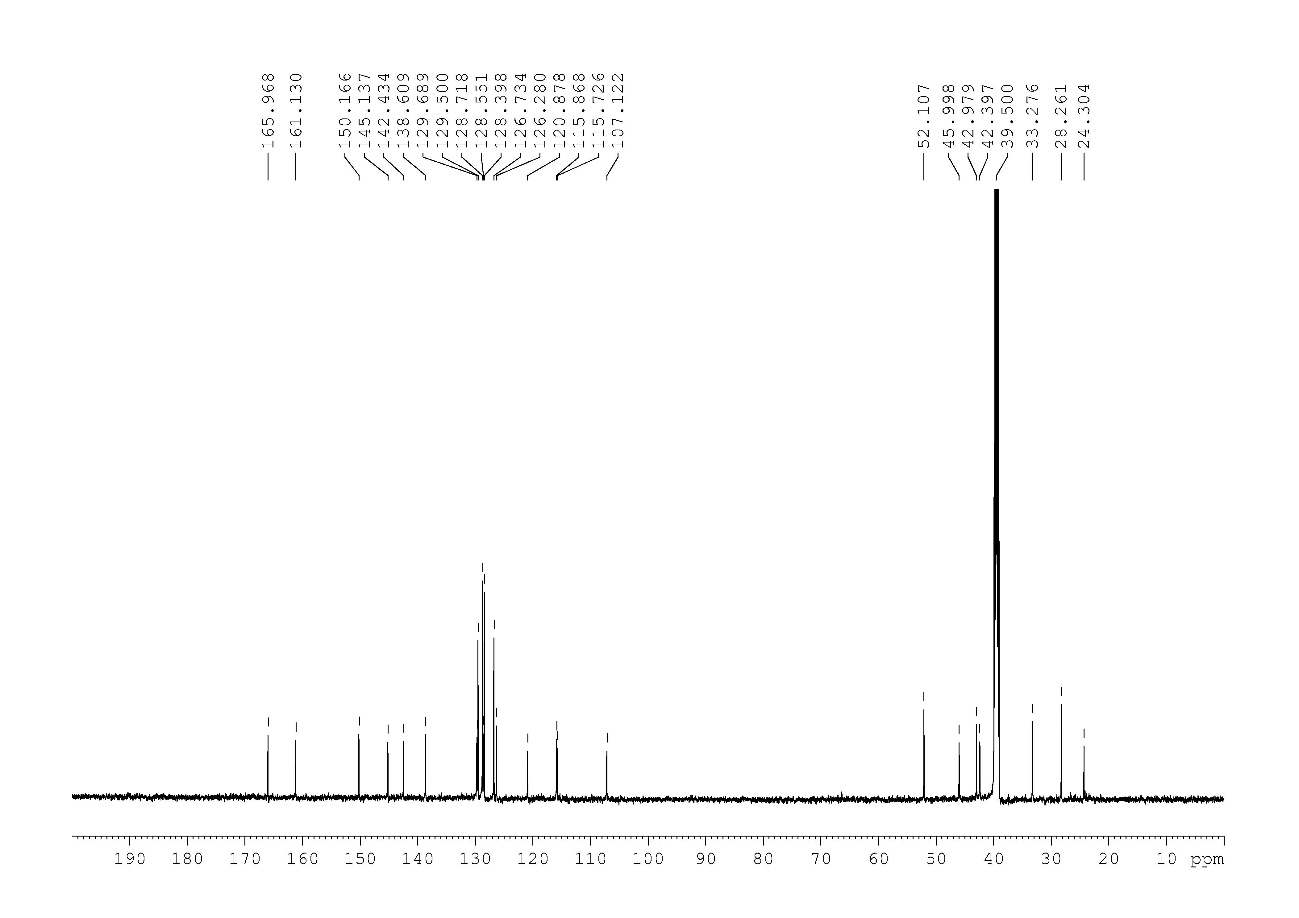


^1^H NMR of **4a** (600 MHz, DMSO-*d*_6_)

^^
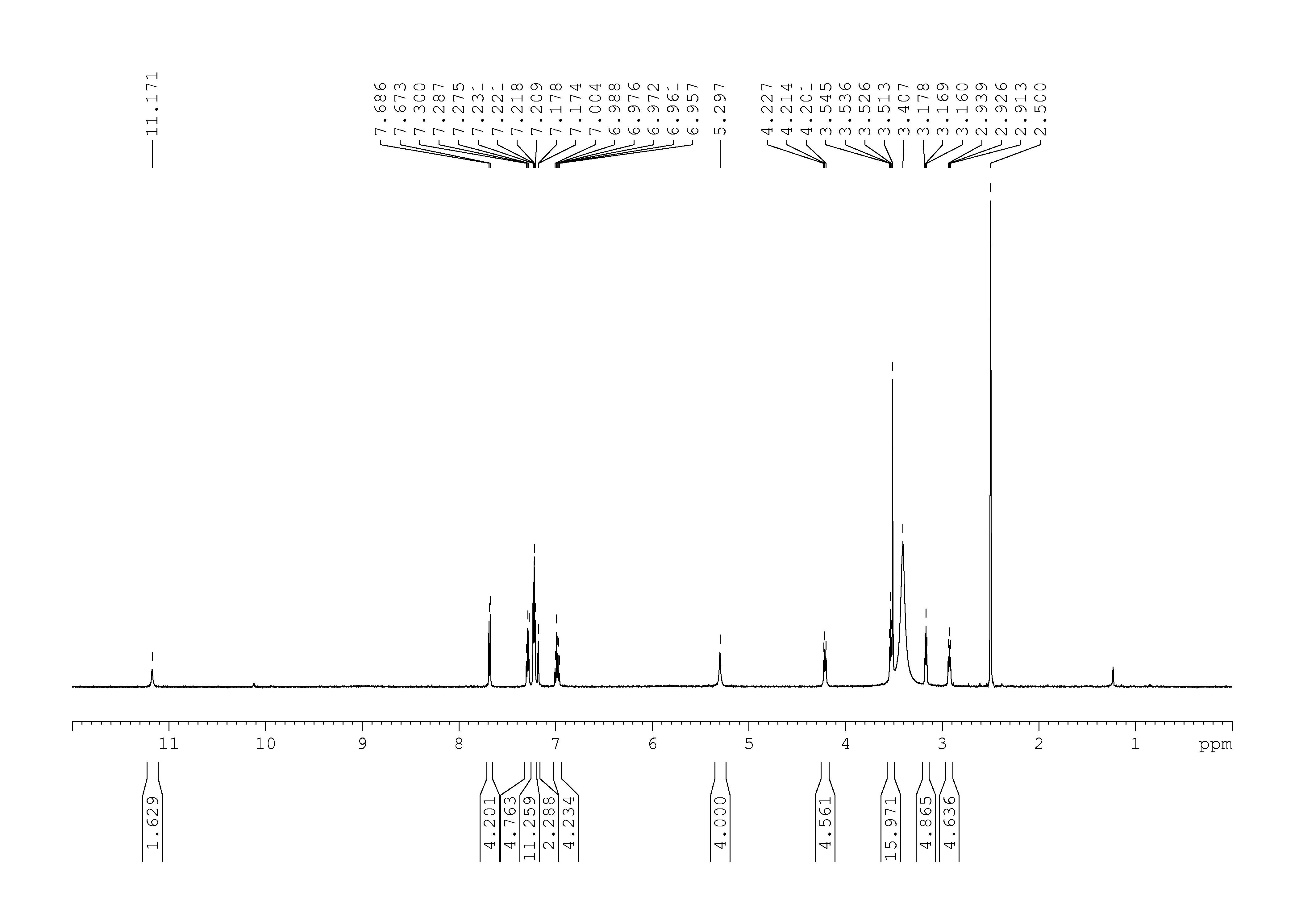


^13^C NMR of **4a** (150 MHz, DMSO-*d*_6_)

^^
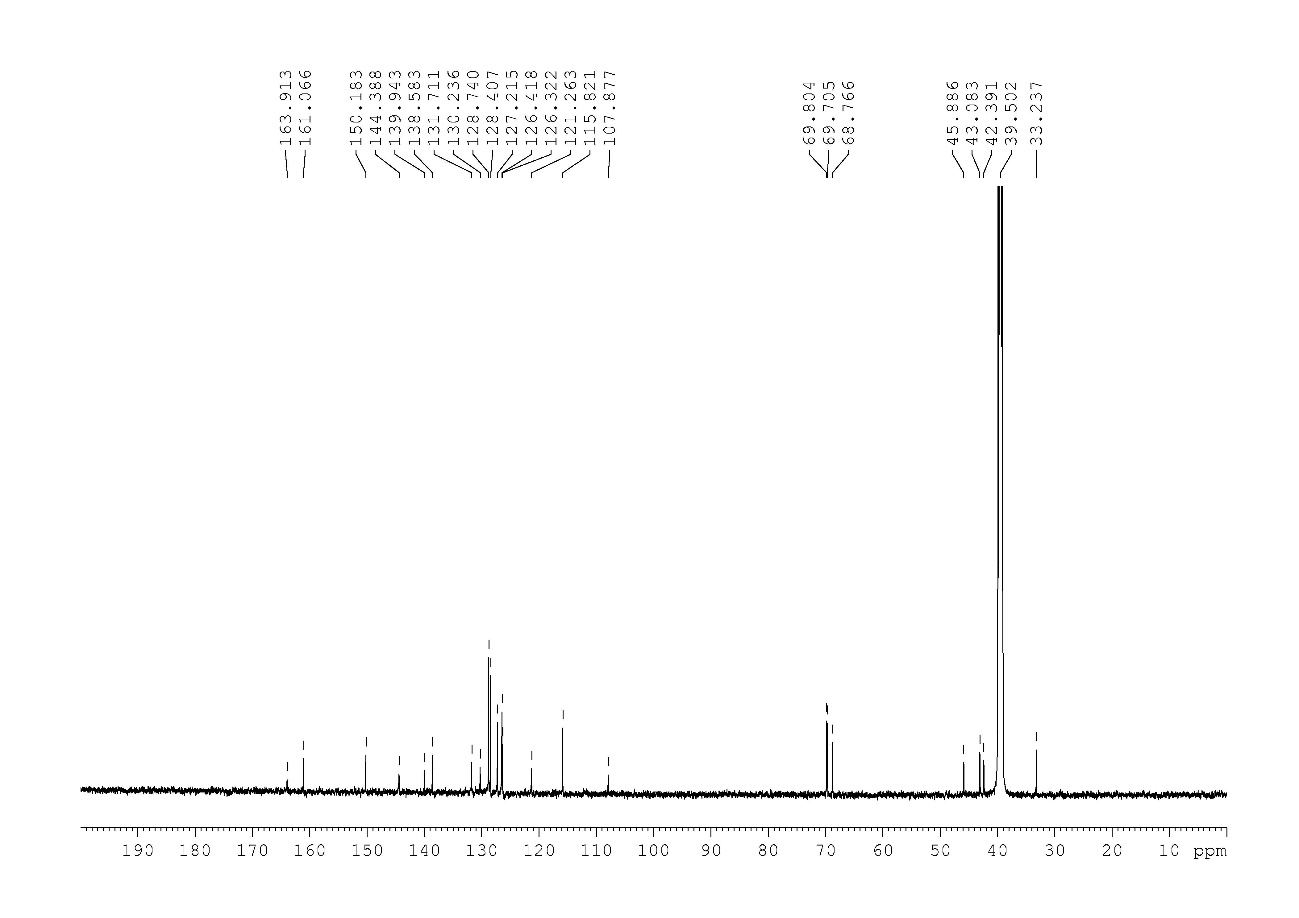


^1^H NMR of **4b** (600 MHz, DMSO-*d*_6_)

^^
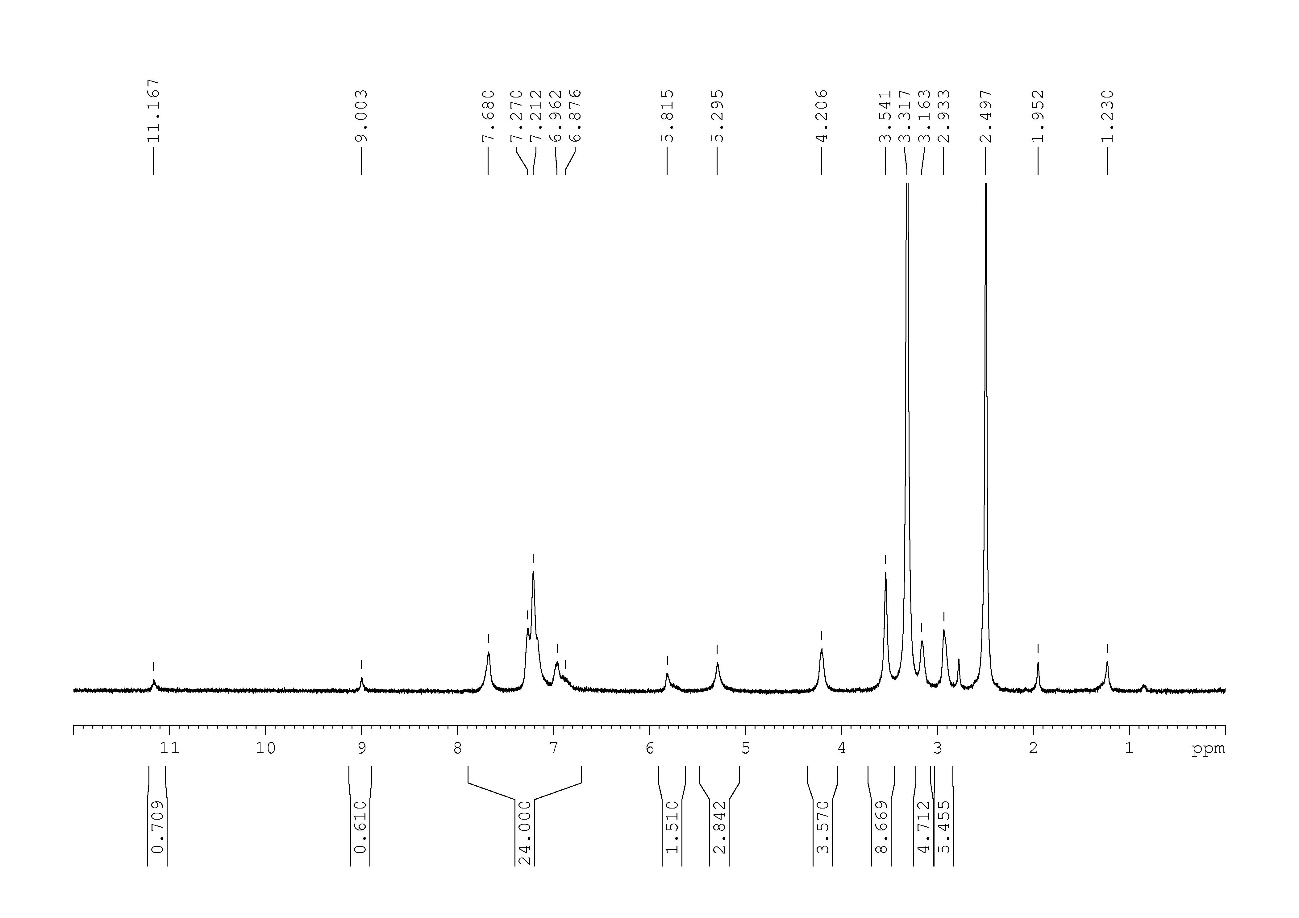


^13^C NMR of **4b** (150 MHz, DMSO-*d*_6_)

^^
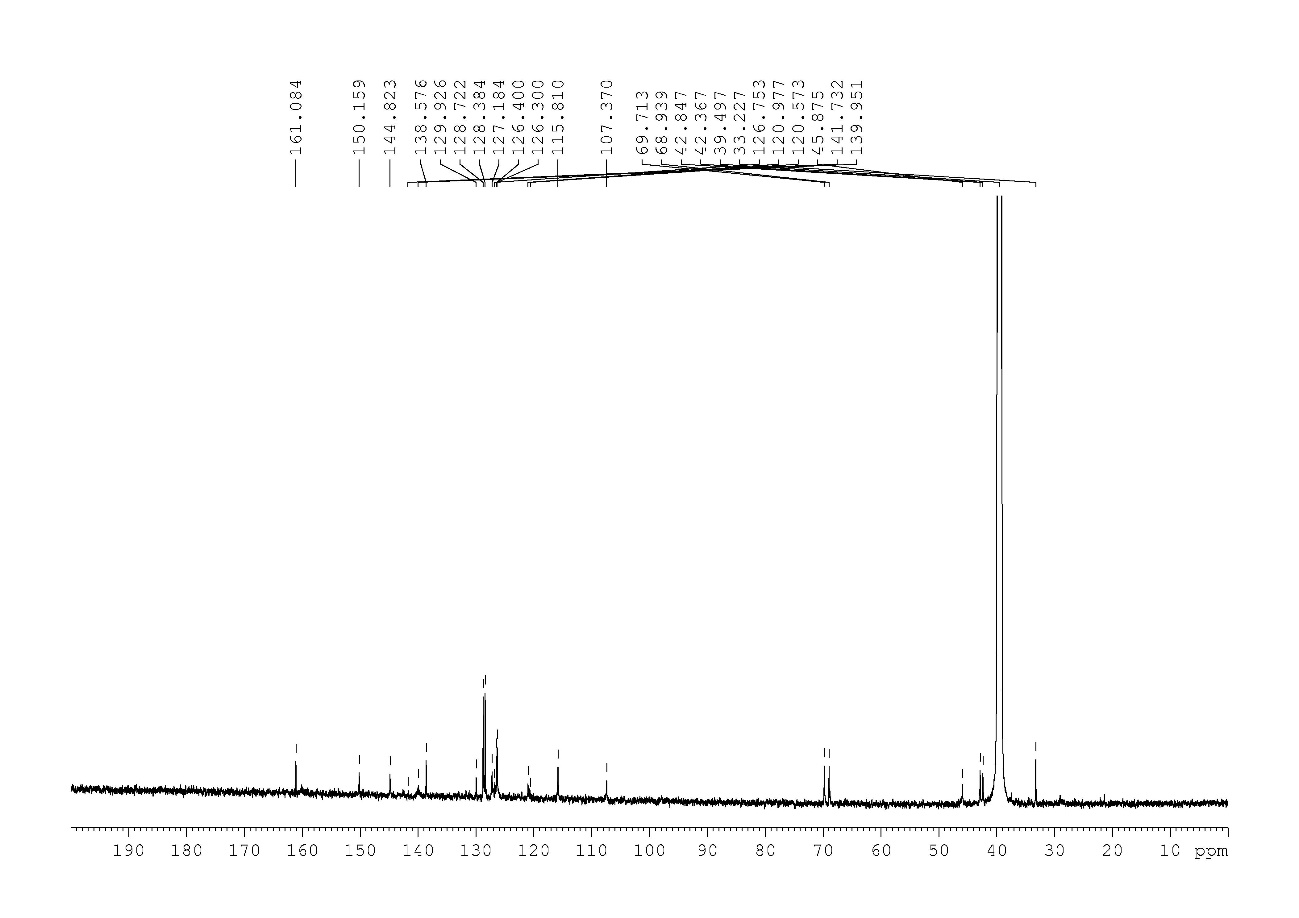


^1^H NMR of **4c** (600 MHz, DMSO-*d*_6_)

^^
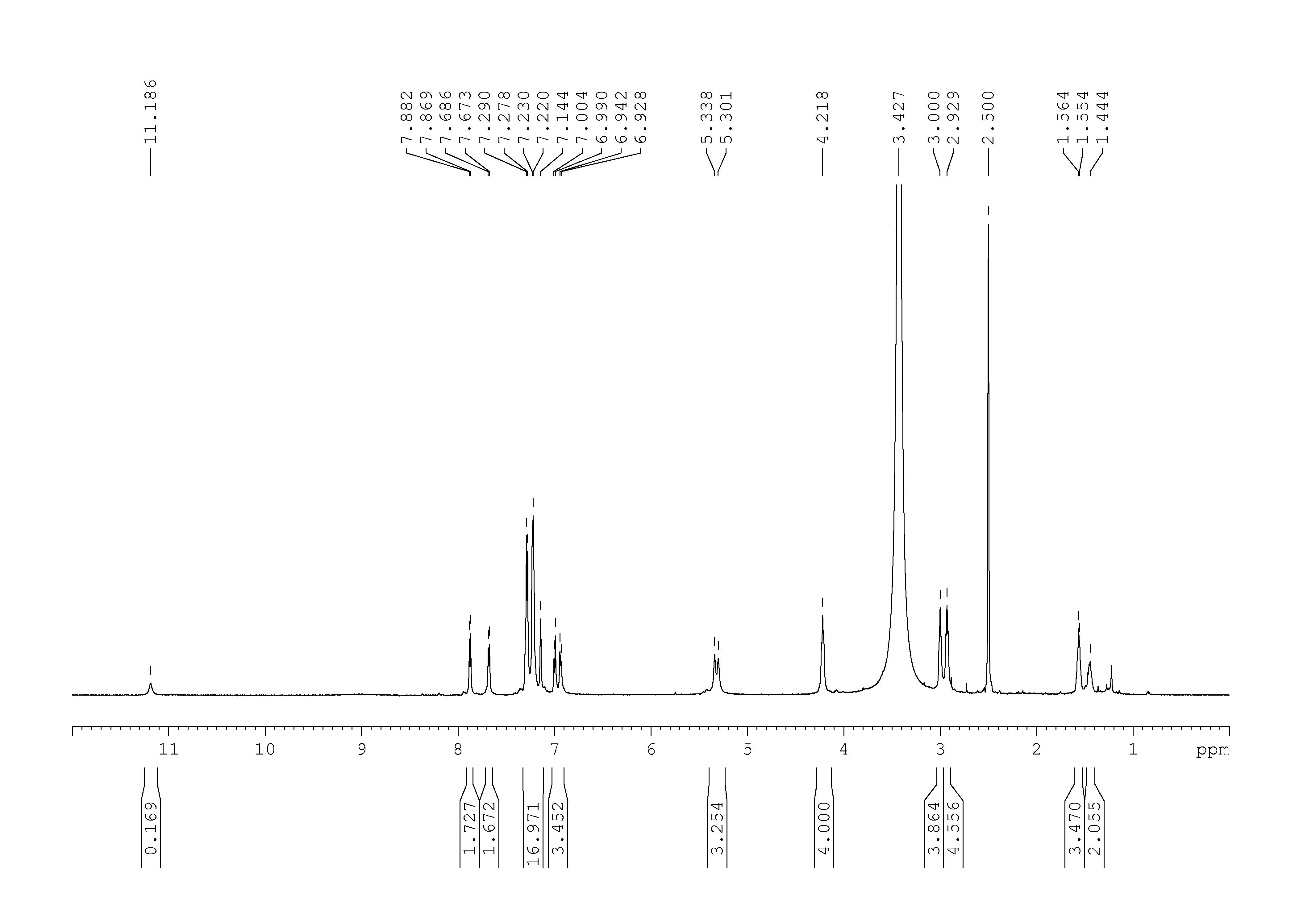


^13^C NMR of **4c** (150 MHz, DMSO-*d*_6_)

^^
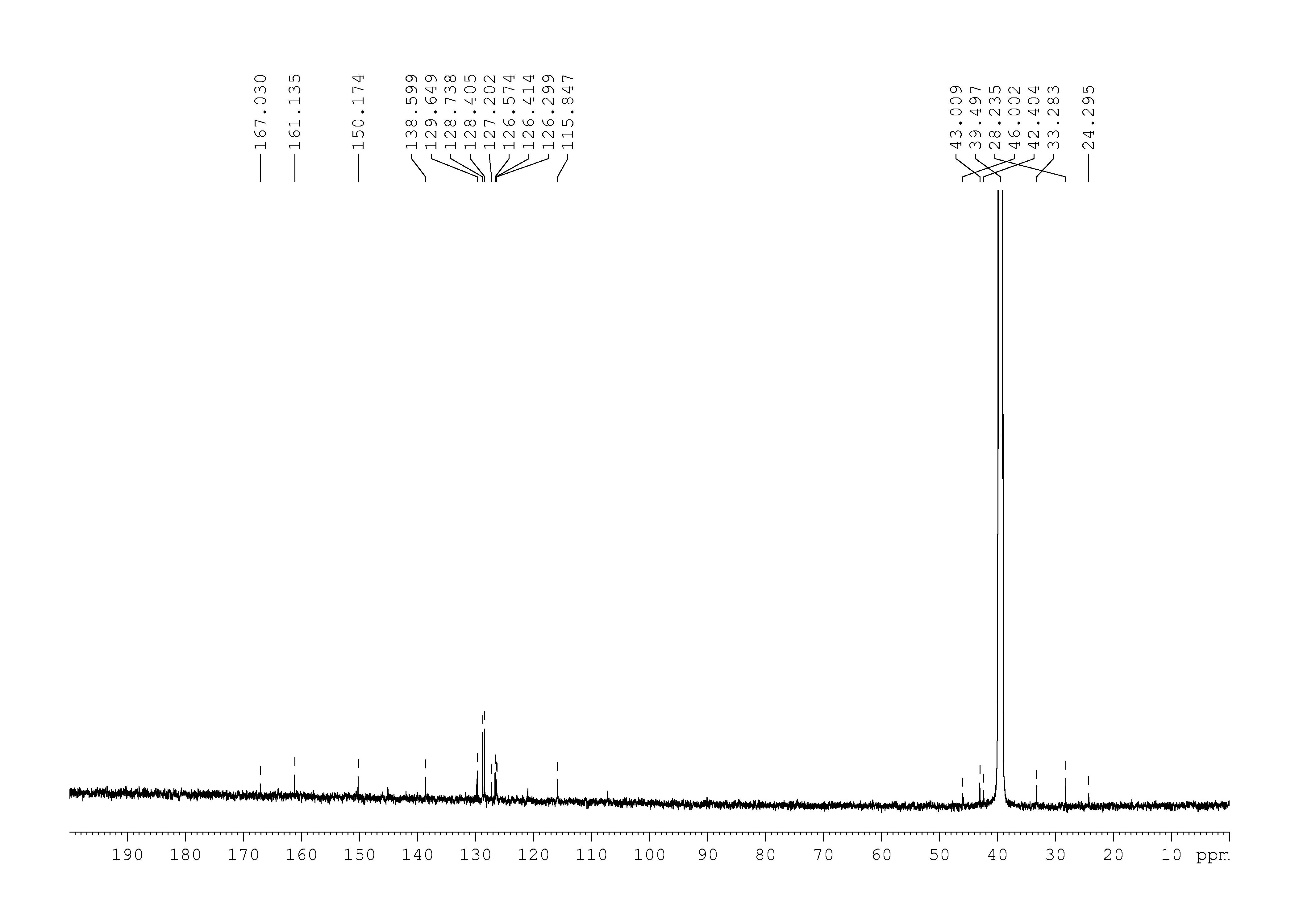


^1^H NMR of **5a** (600 MHz, DMSO-*d*_6_)

^^
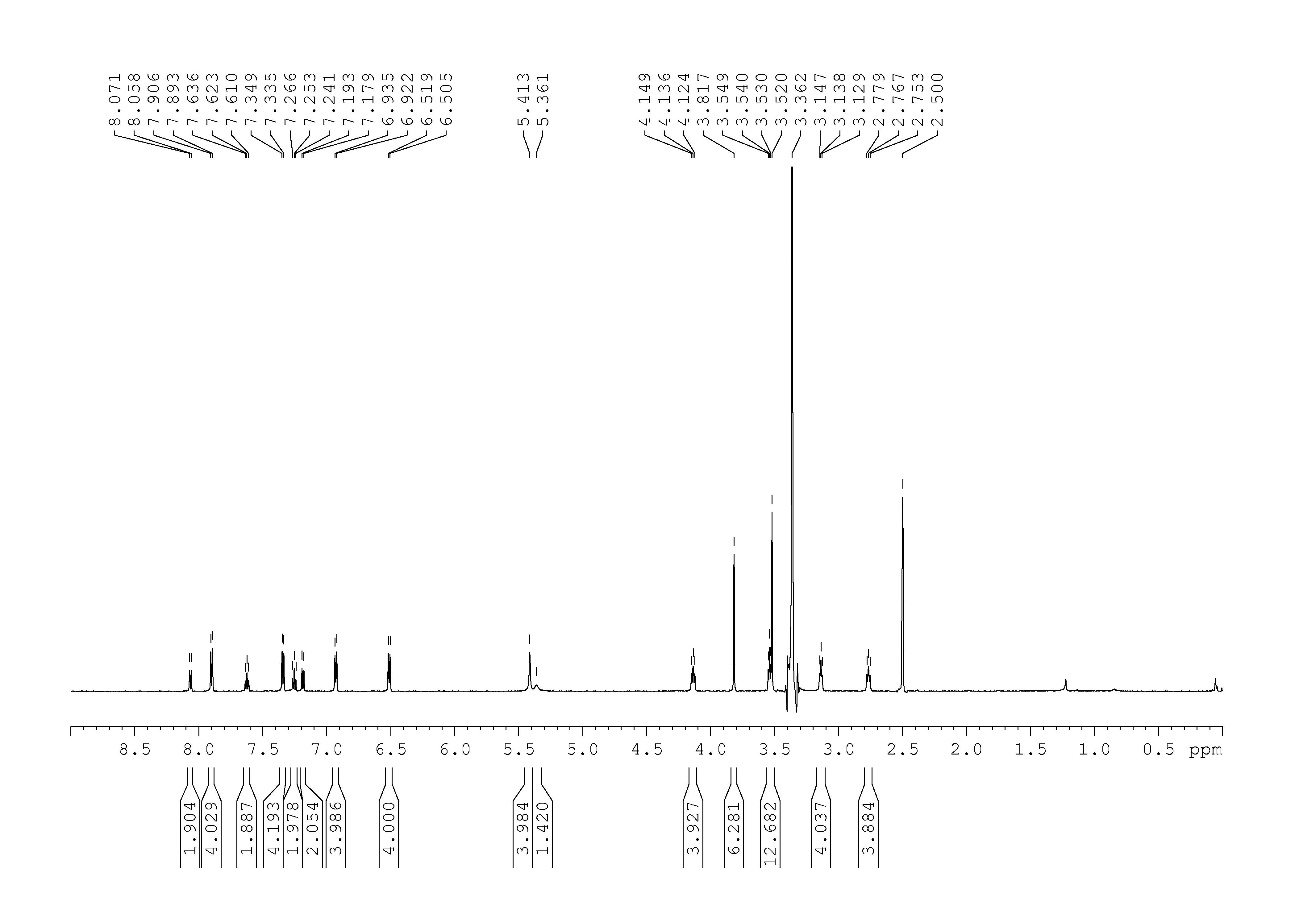


^13^C NMR of **5a** (150 MHz, DMSO-*d*_6_)

^^
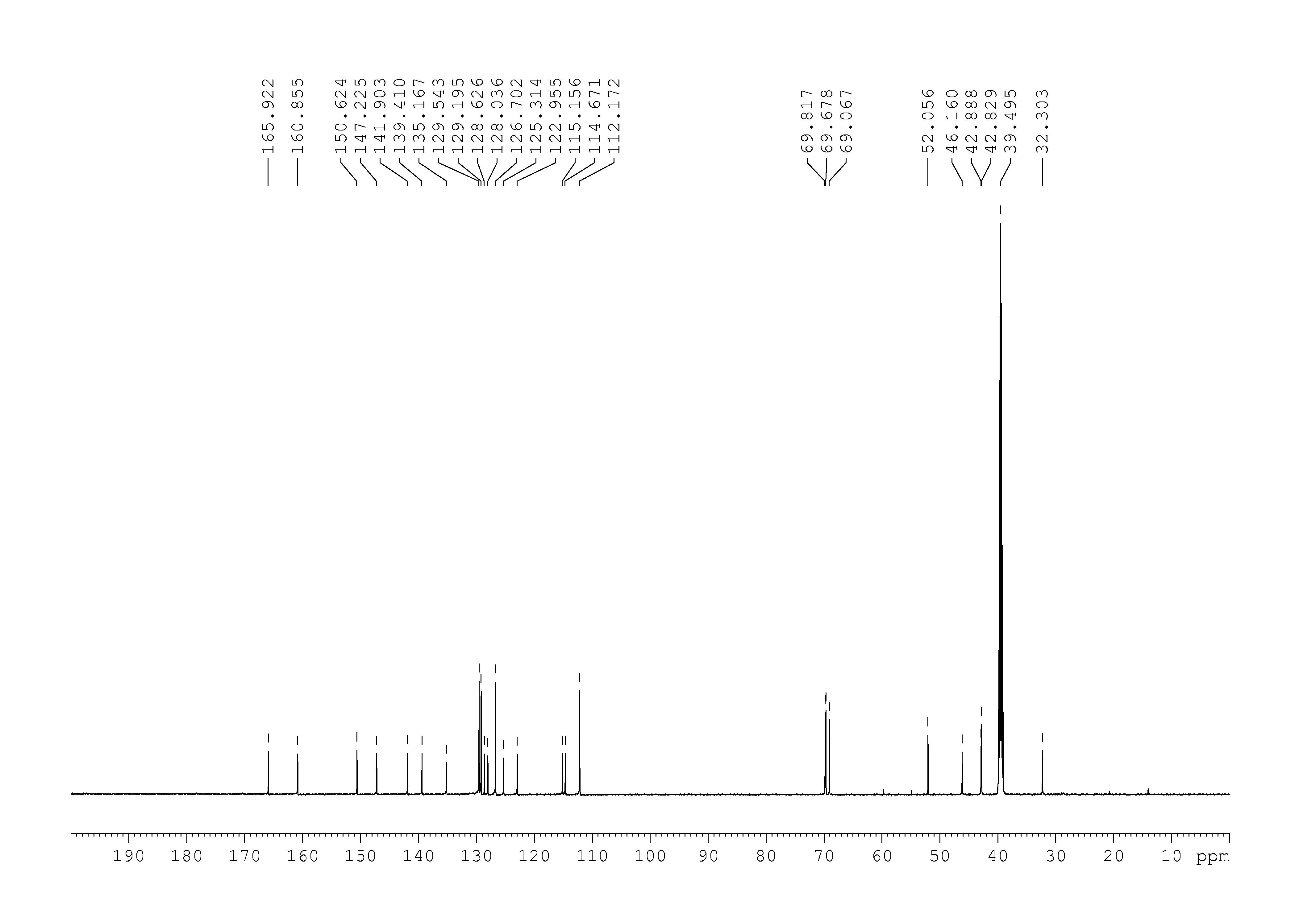


^1^H NMR of **5b** (600 MHz, DMSO-*d*_6_)

^^
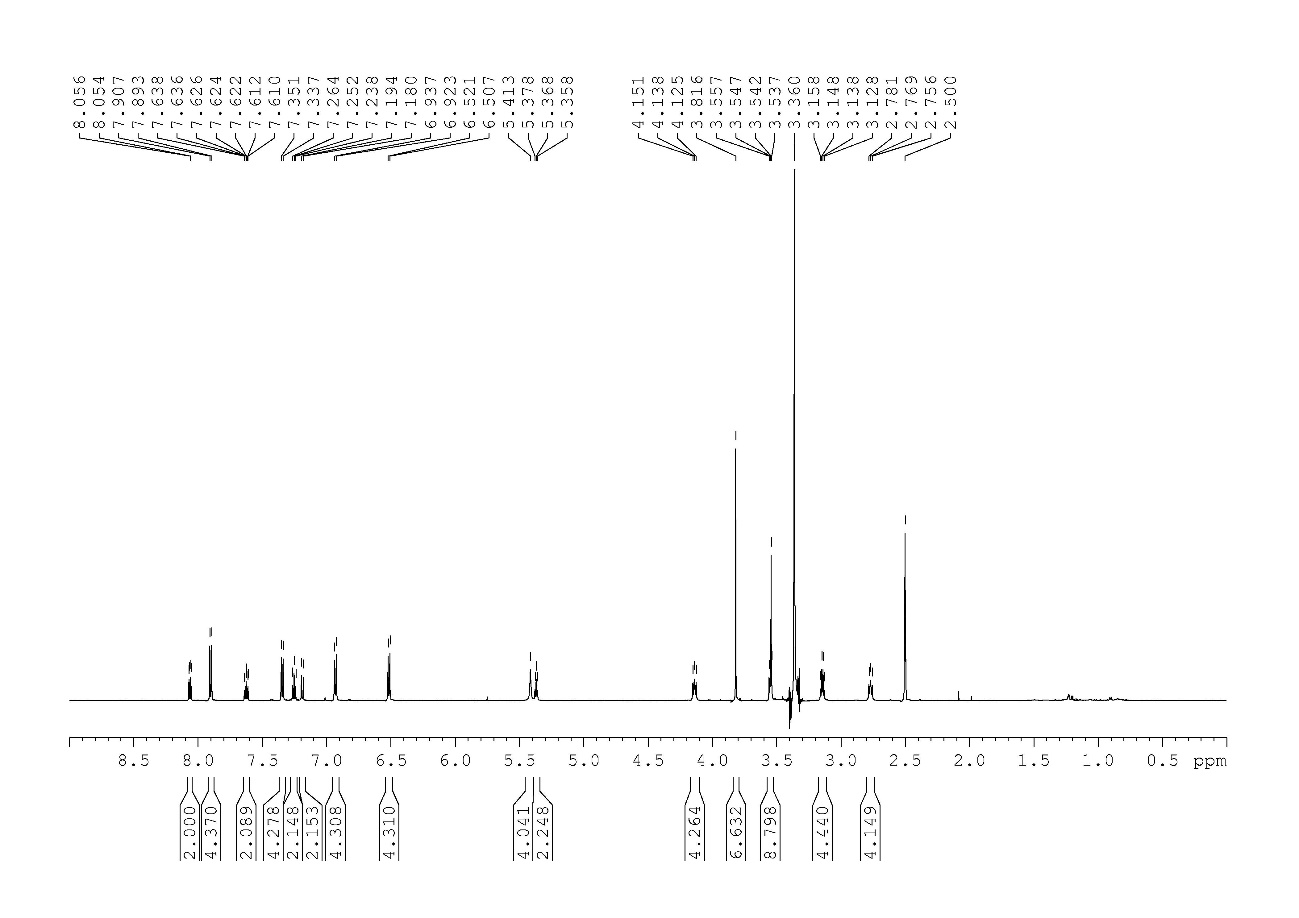


^13^C NMR of **5b** (150 MHz, DMSO-*d*_6_)

^^
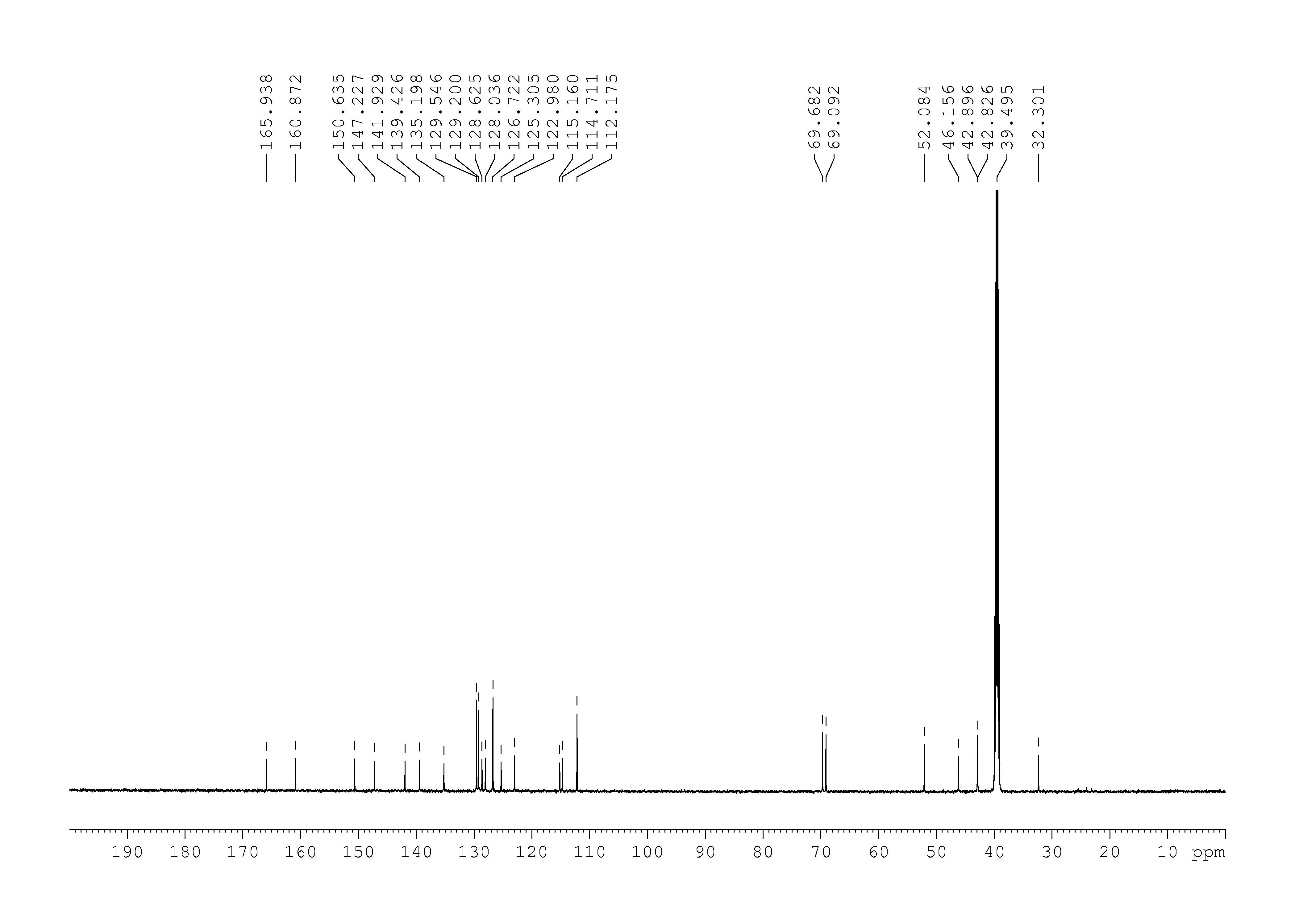


^1^H NMR of **5c** (600 MHz, DMSO-*d*_6_)

^^
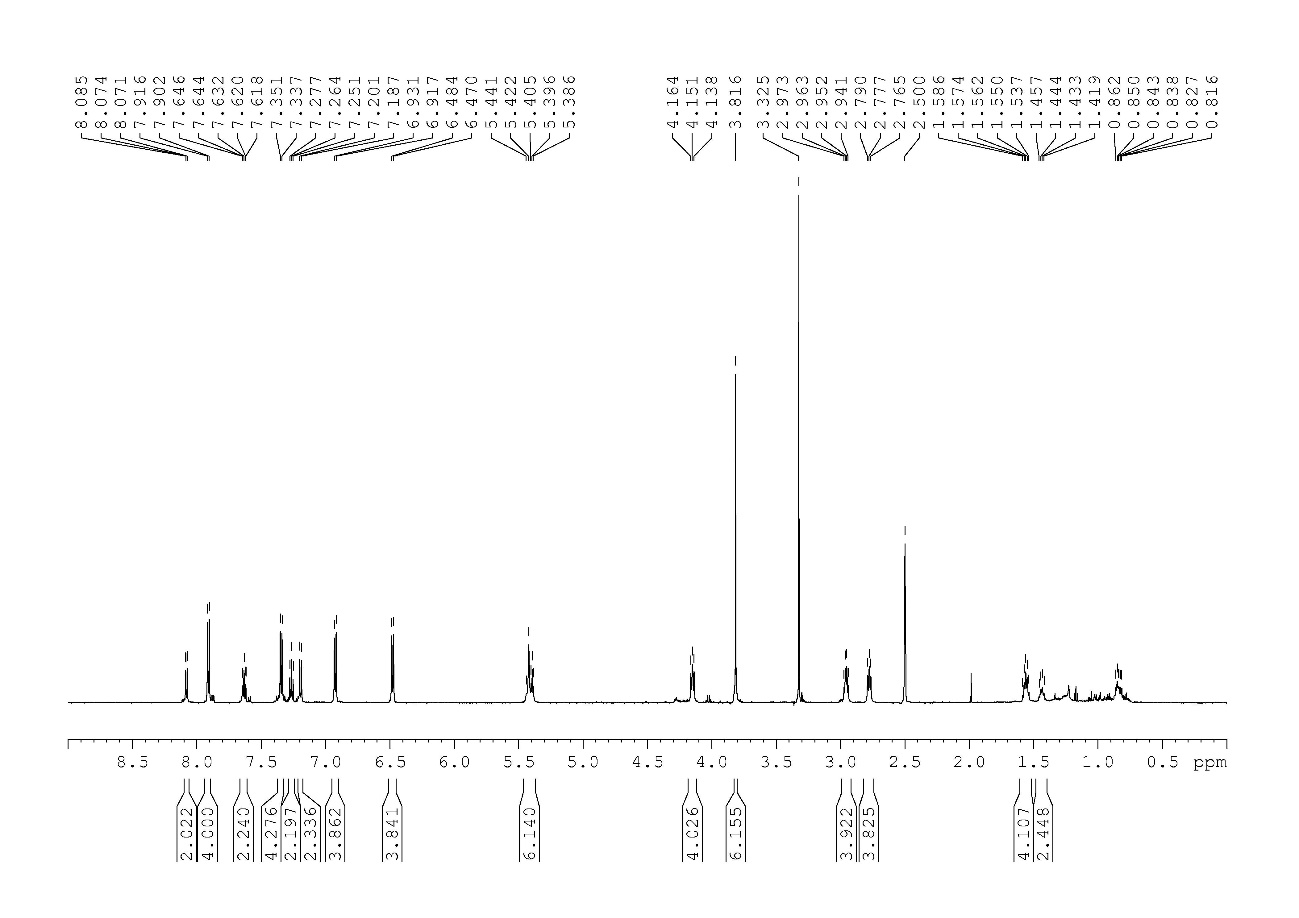


^13^C NMR of **5c** (150 MHz, DMSO-*d*_6_)

^^
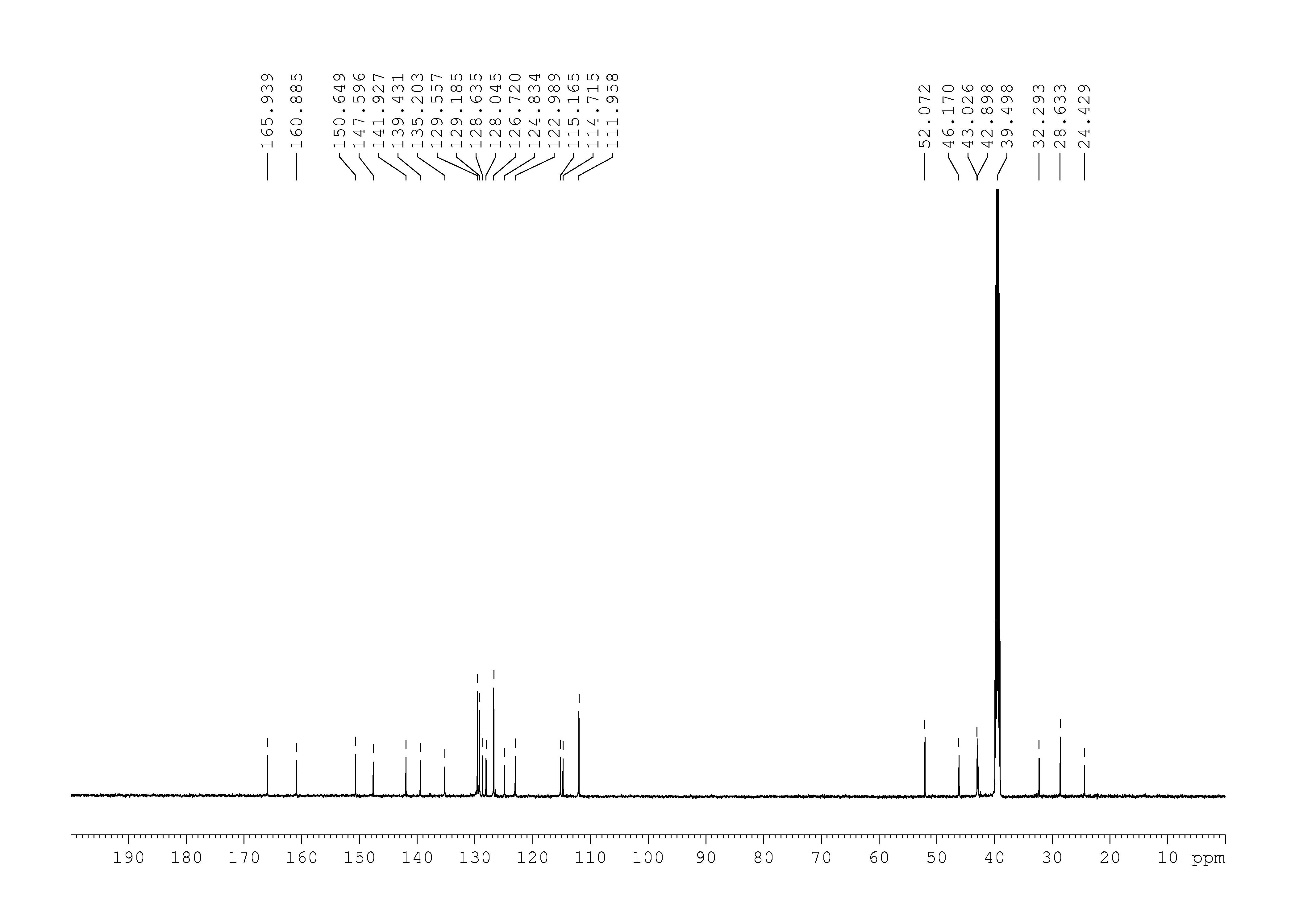


^1^H NMR of **6a** (600 MHz, DMSO-*d*_6_)


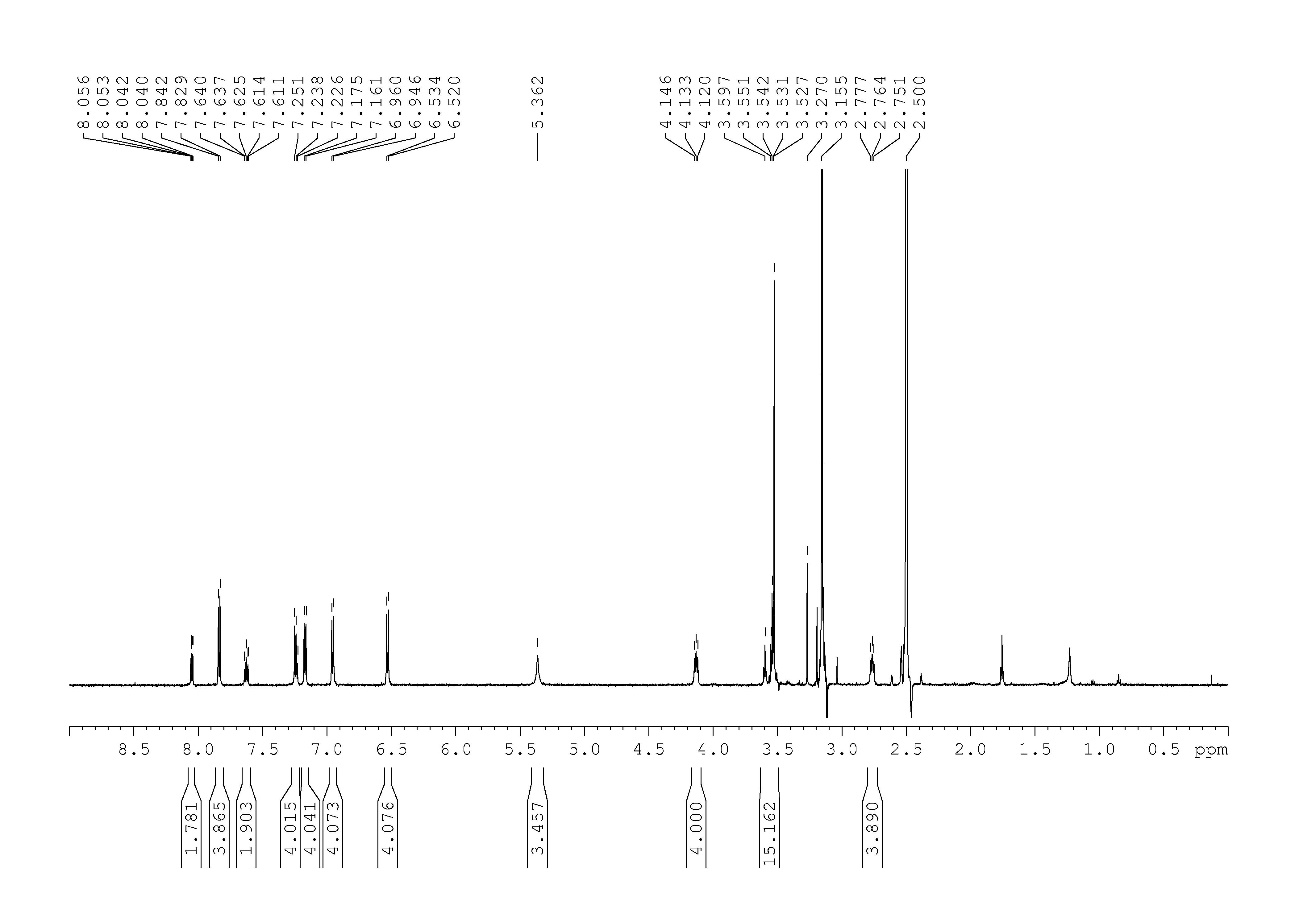


^13^C NMR of **6a** (150 MHz, DMSO-*d*_6_)

^^
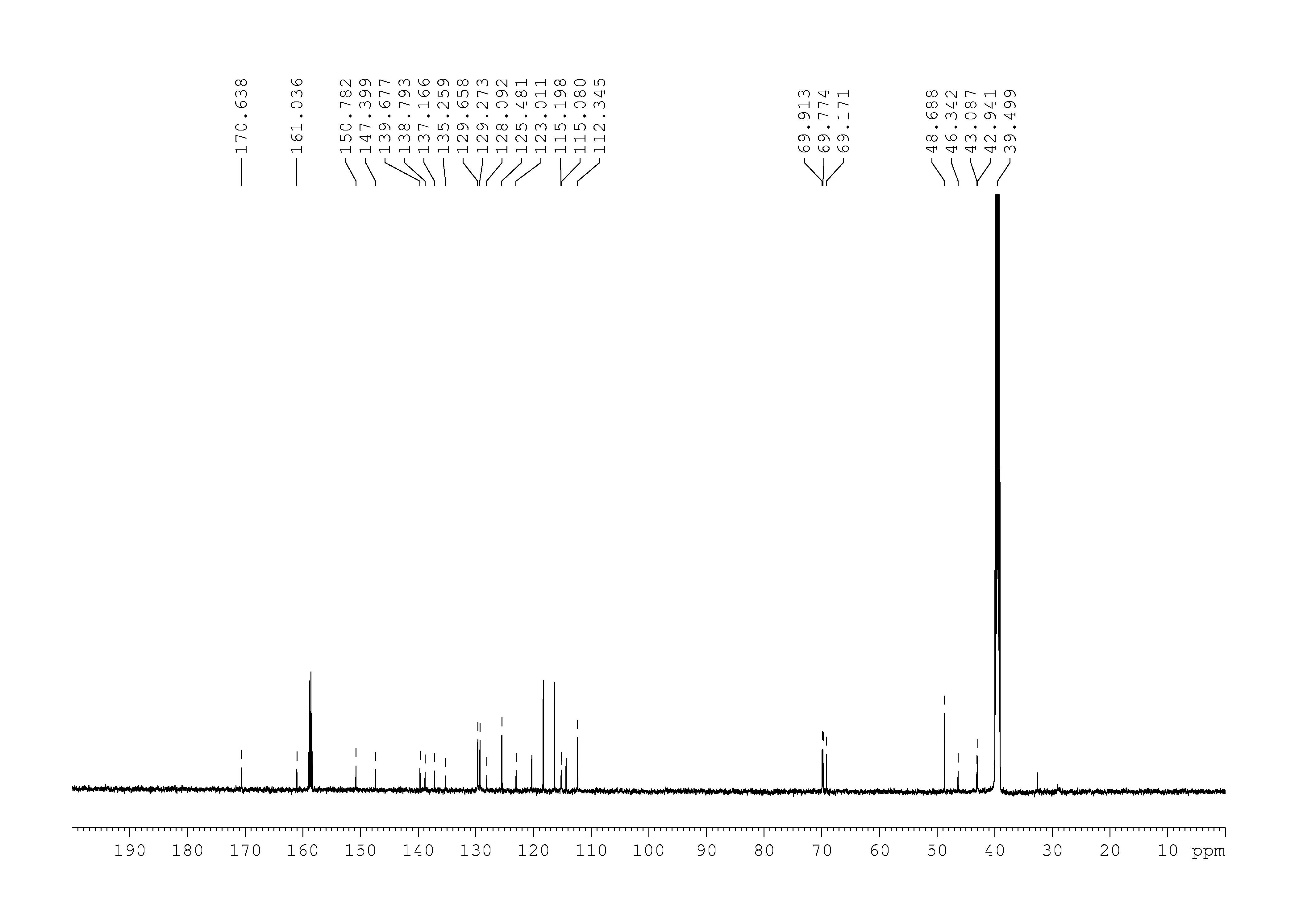


^1^H NMR of **6b** (600 MHz, DMSO-*d*_6_)

^^
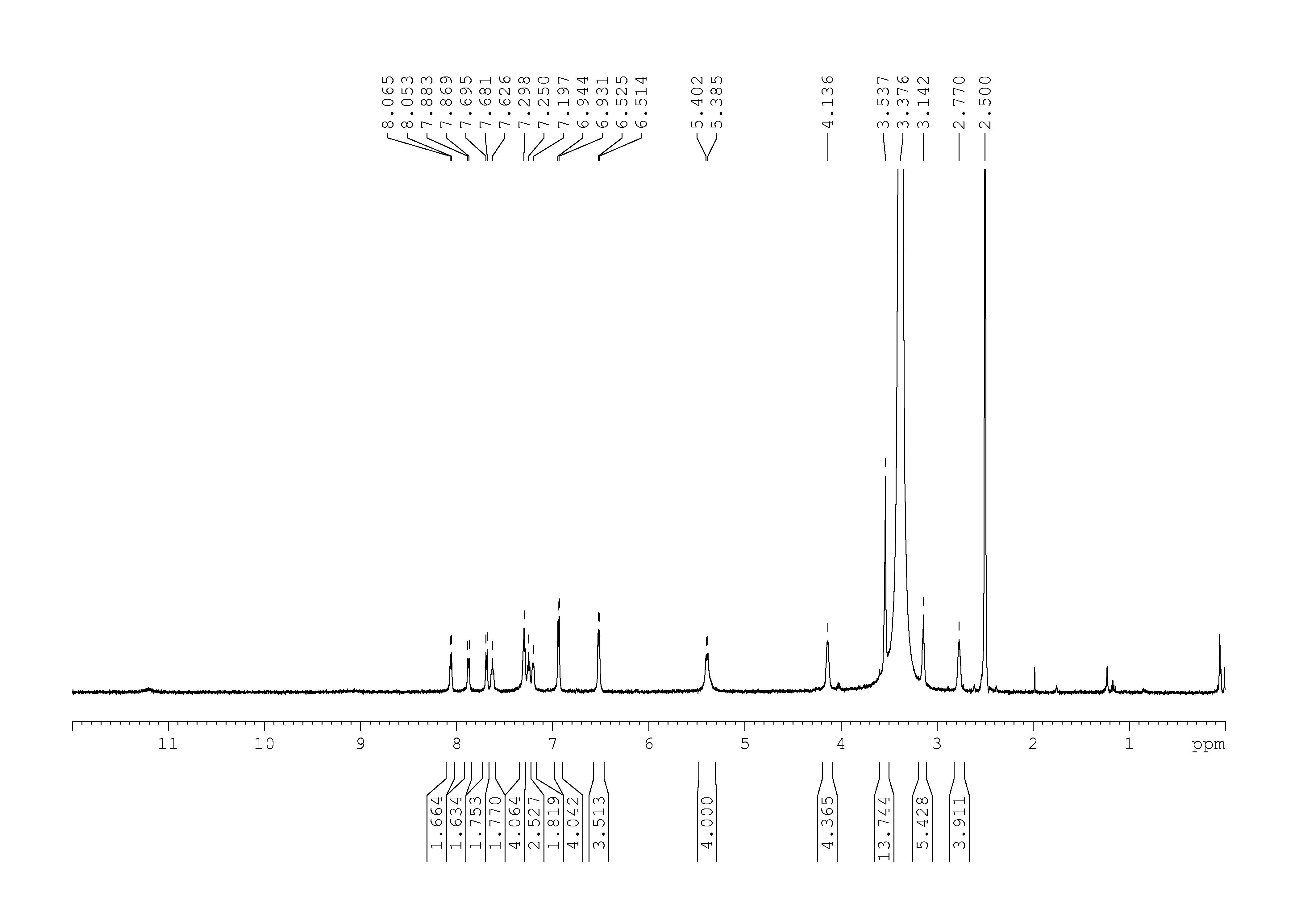


^13^C NMR of **6b** (150 MHz, DMSO-*d*_6_)


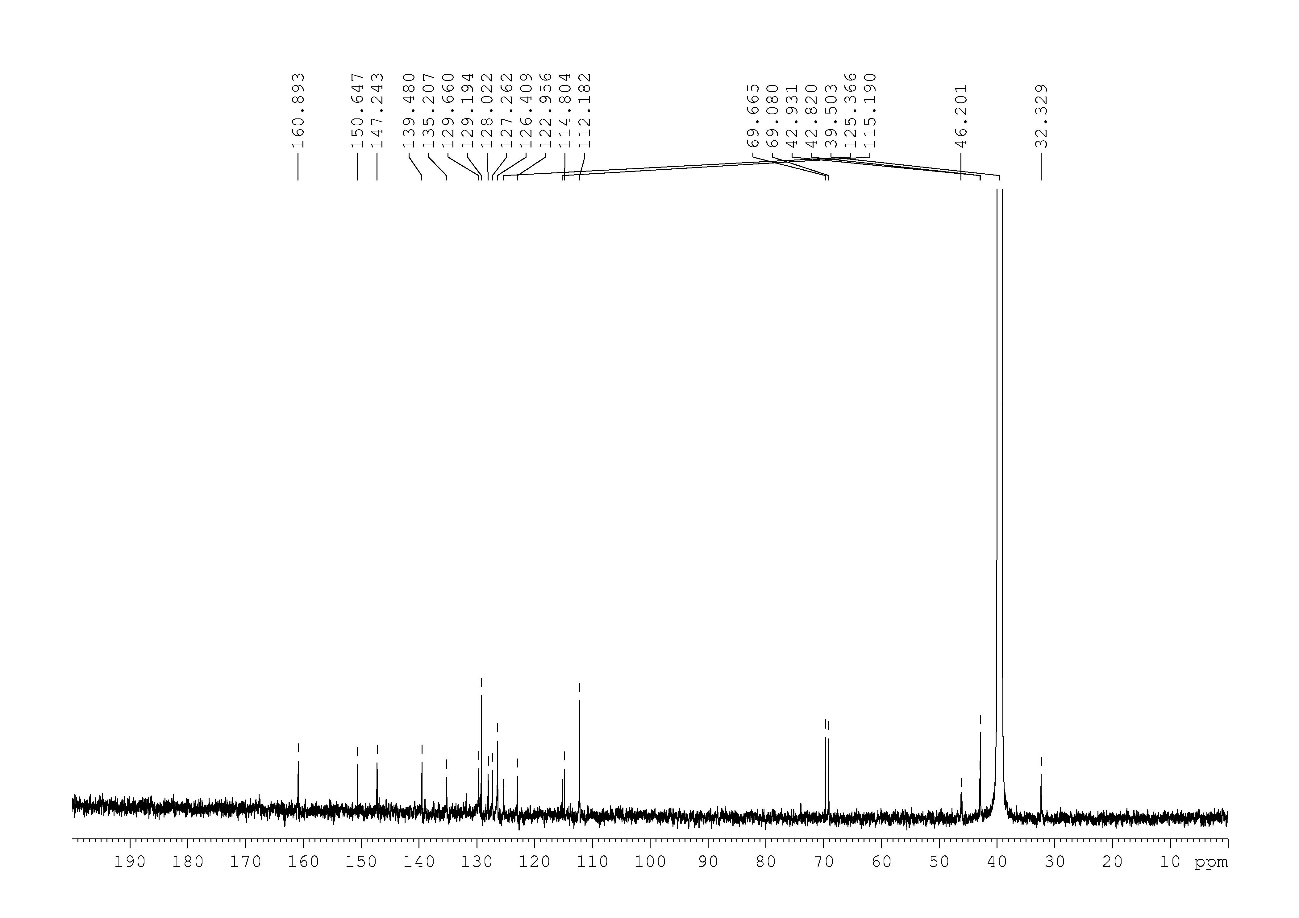


^1^H NMR of **6c** (600 MHz, DMSO-*d*_6_)

^^
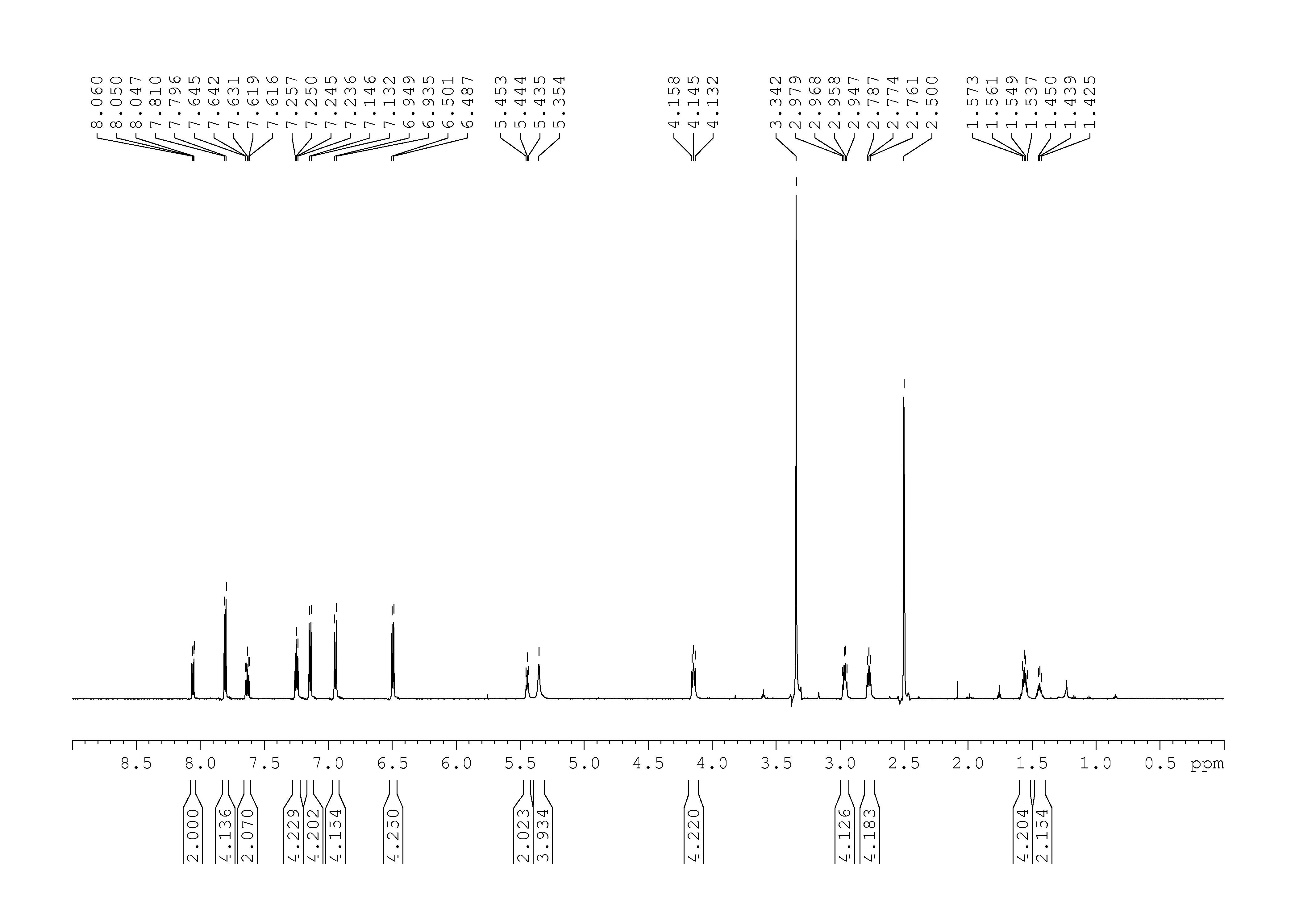


^13^C NMR of **6c** (150 MHz, DMSO-*d*_6_)

^^
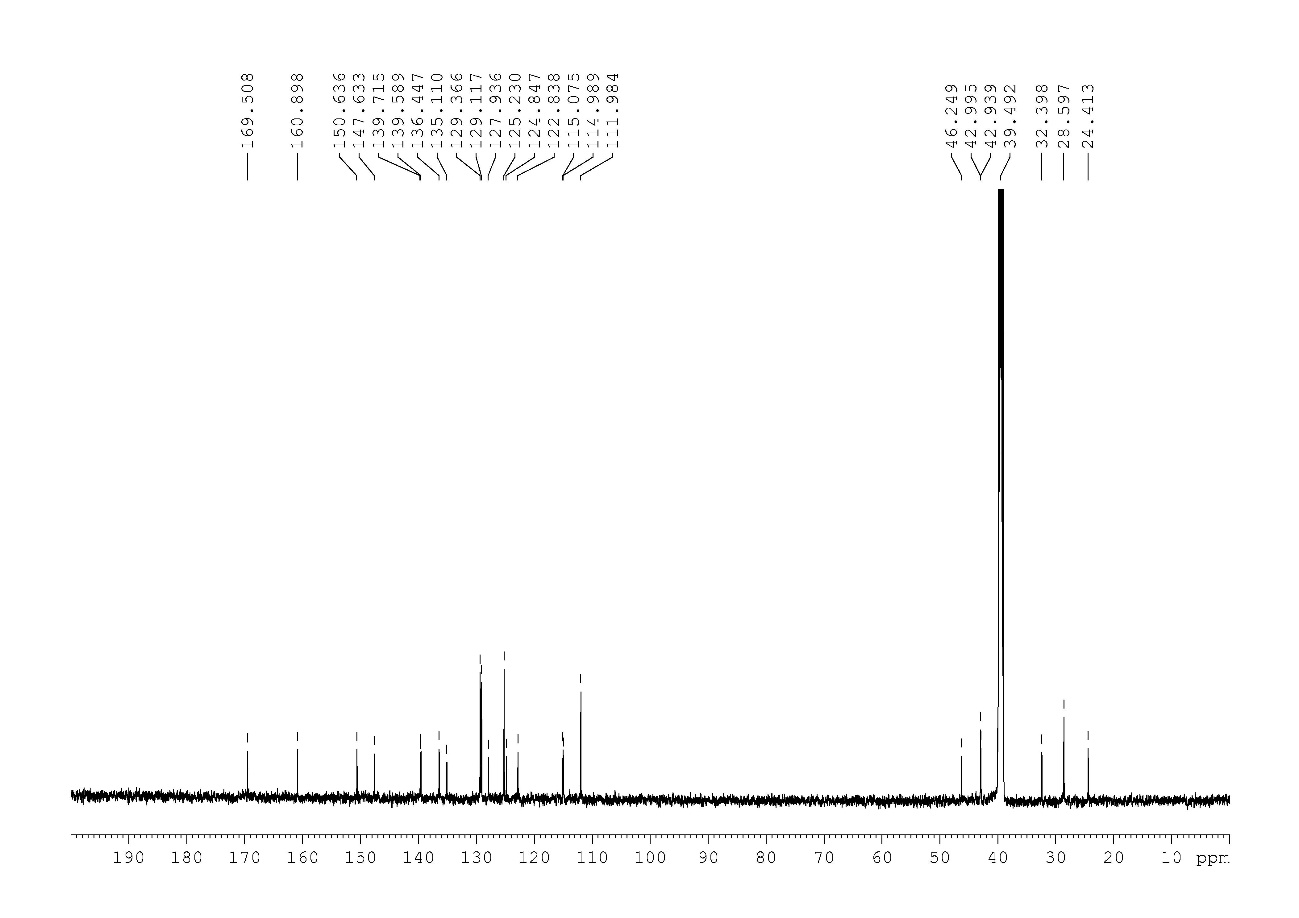


^1^H NMR of **7a** (600 MHz, DMSO-*d*_6_)

^^
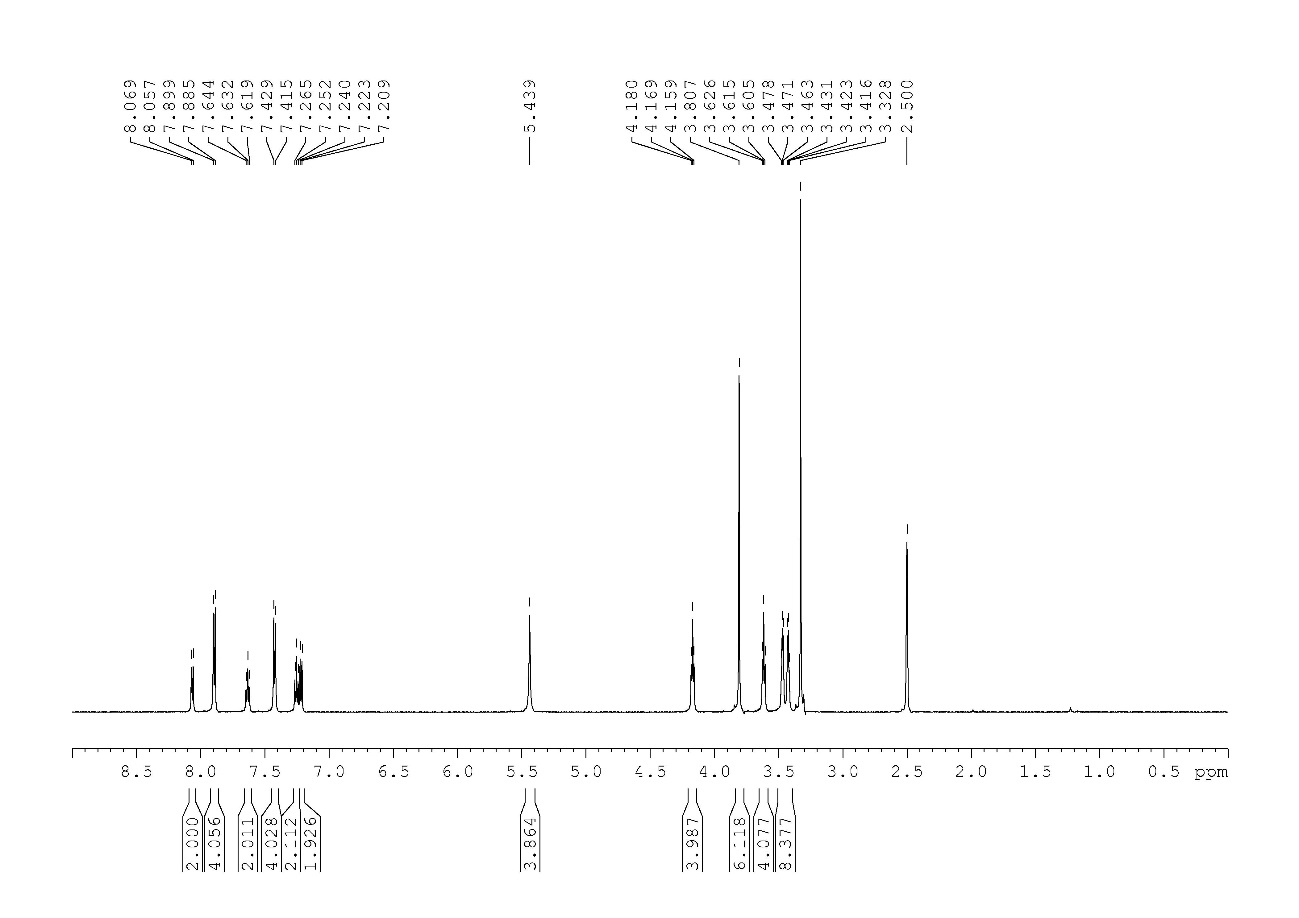


^13^C NMR of **7a** (150 MHz, DMSO-*d*_6_)

^^
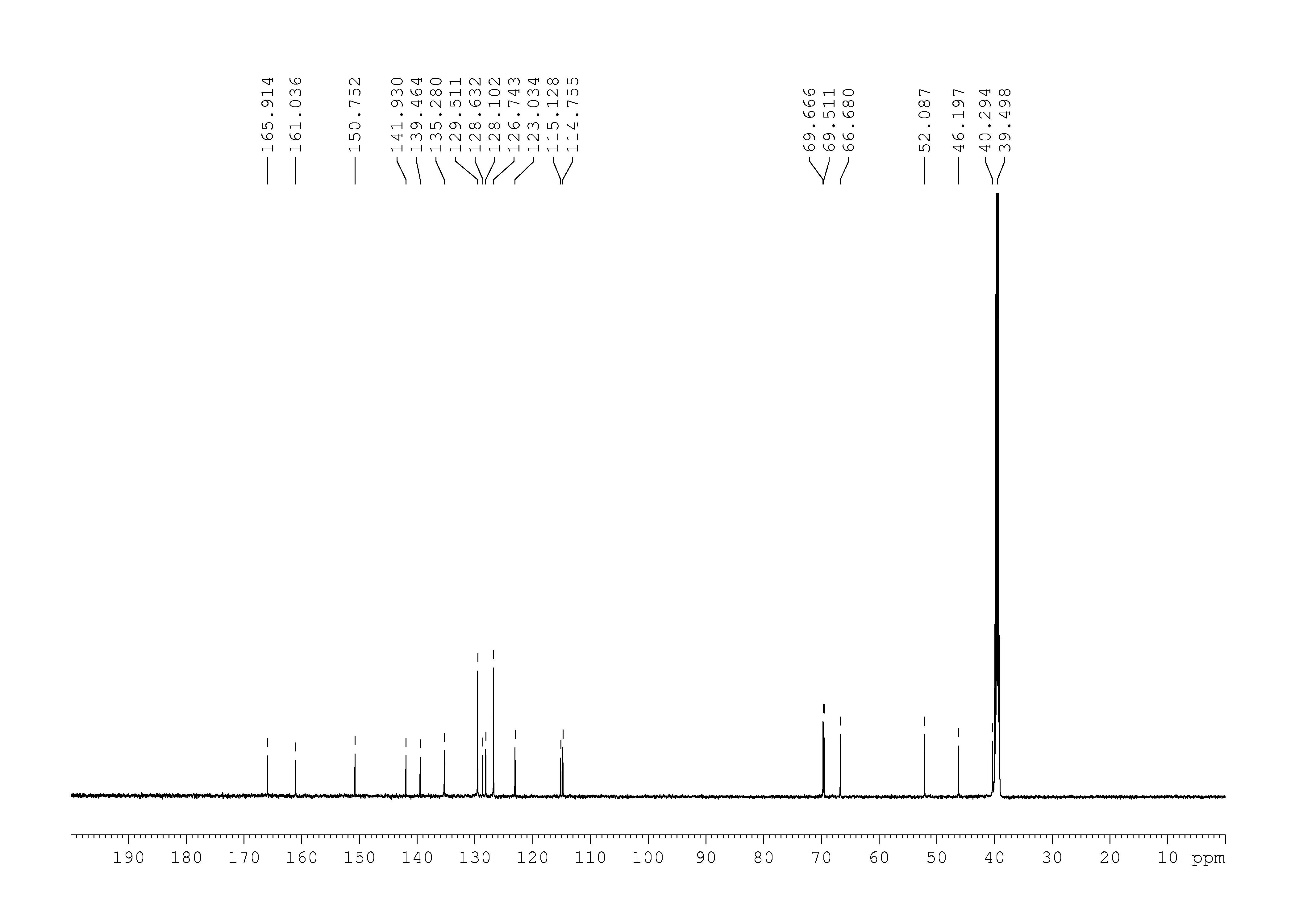


^1^H NMR of **7b** (600 MHz, DMSO-*d*_6_)

^^
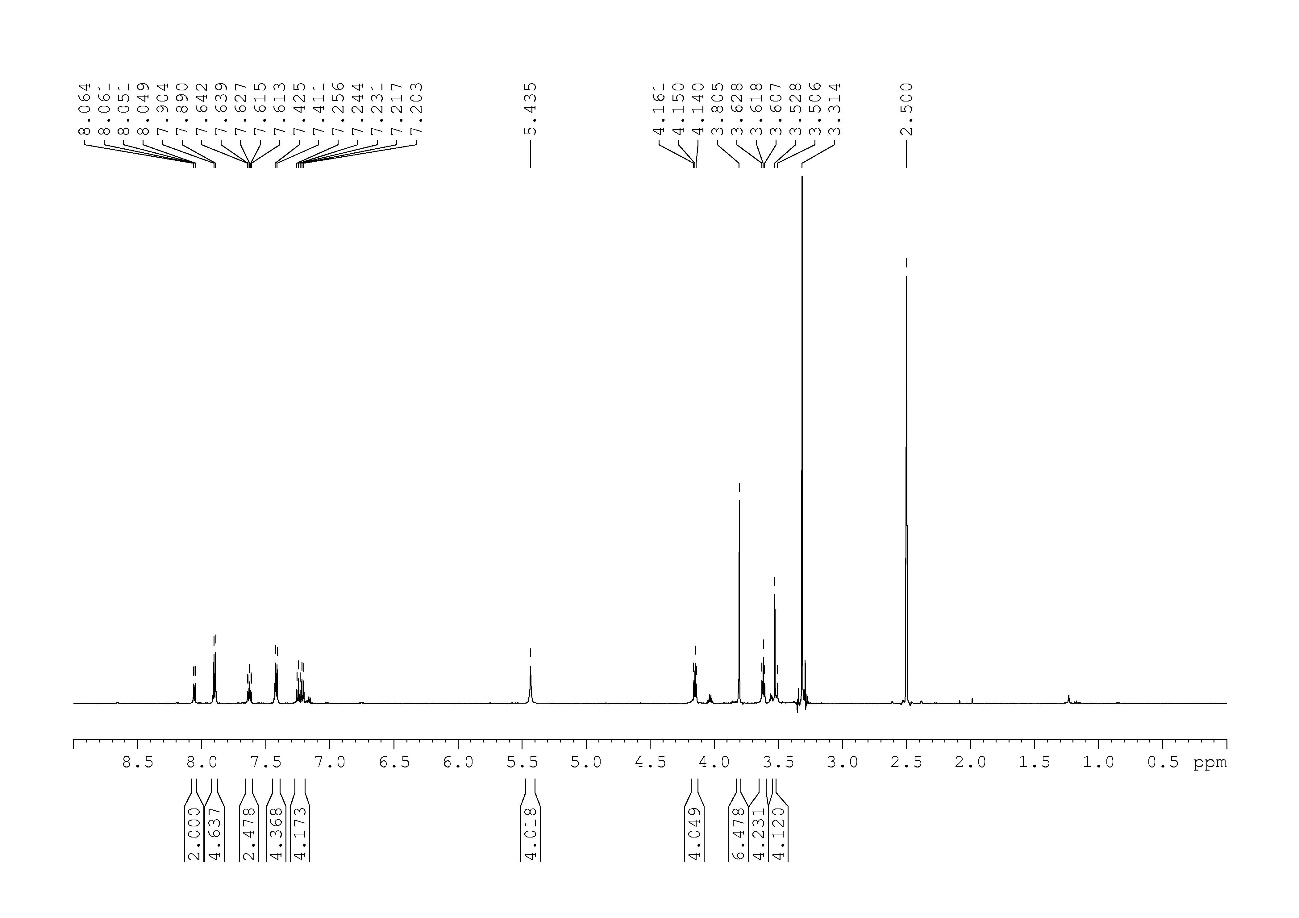


^13^C NMR of **7b** (150 MHz, DMSO-*d*_6_)

^^
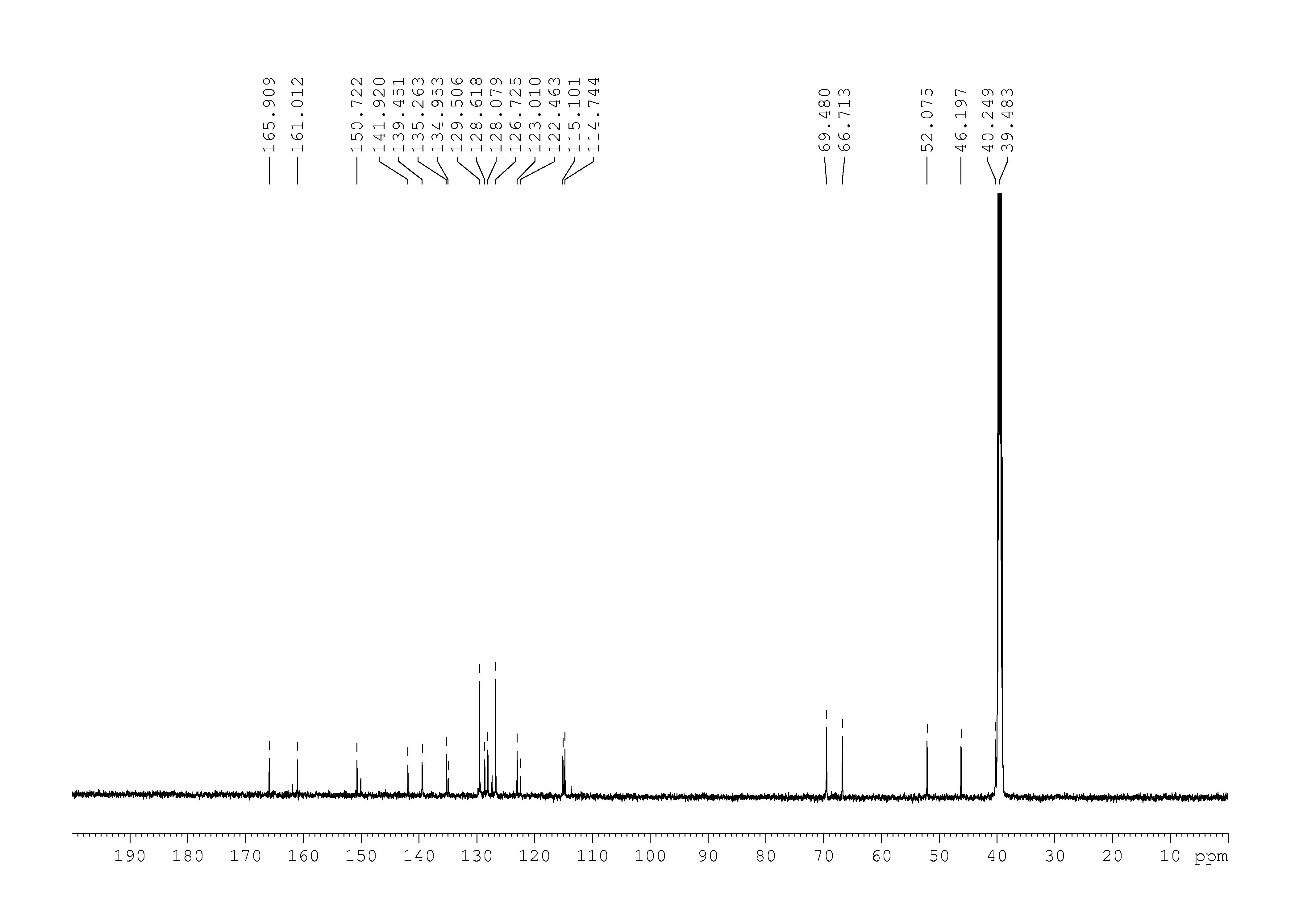


^1^H NMR of **7c** (600 MHz, DMSO-*d*_6_)

^^
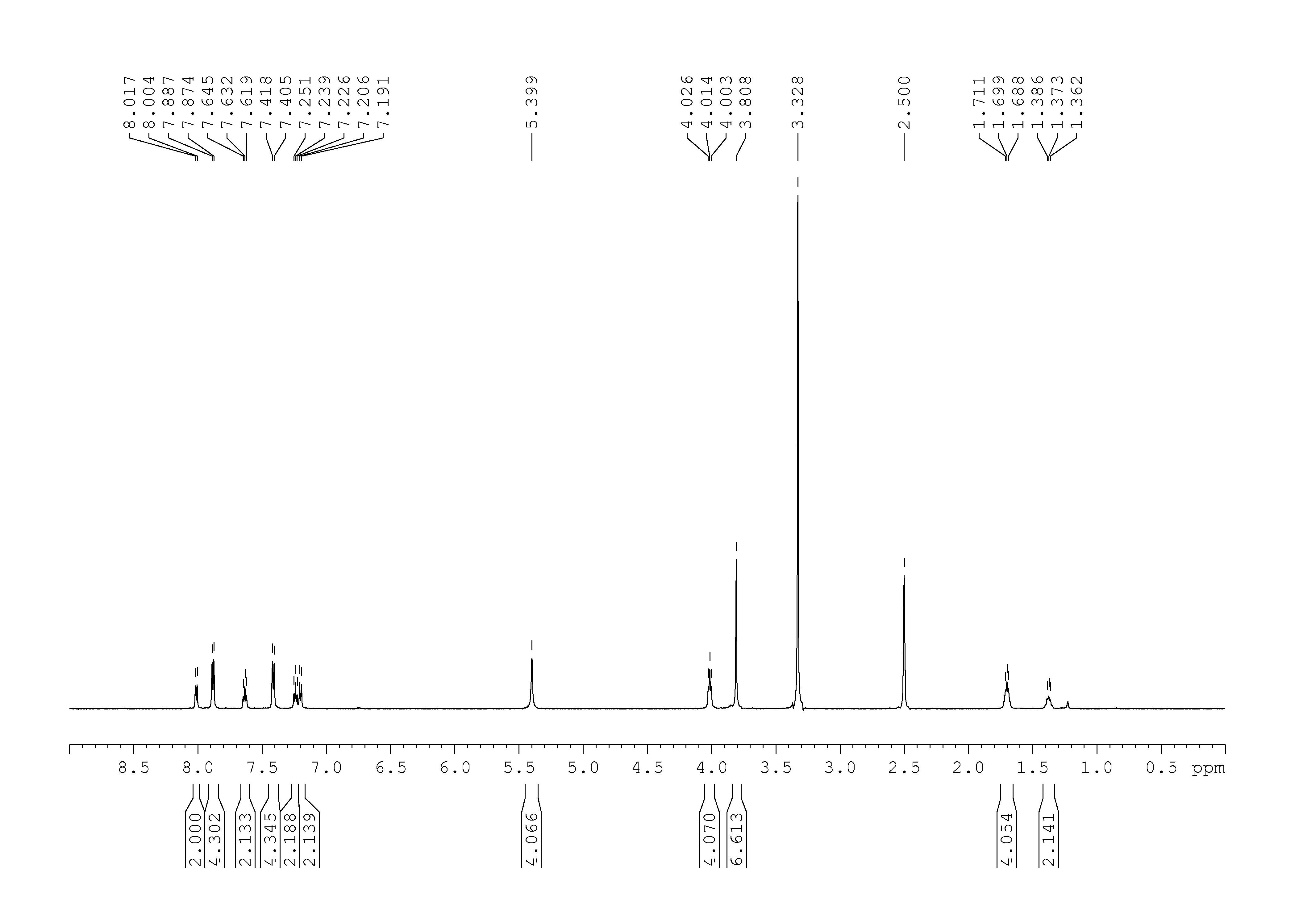


^13^C NMR of **7c** (150 MHz, DMSO-*d*_6_)

^^
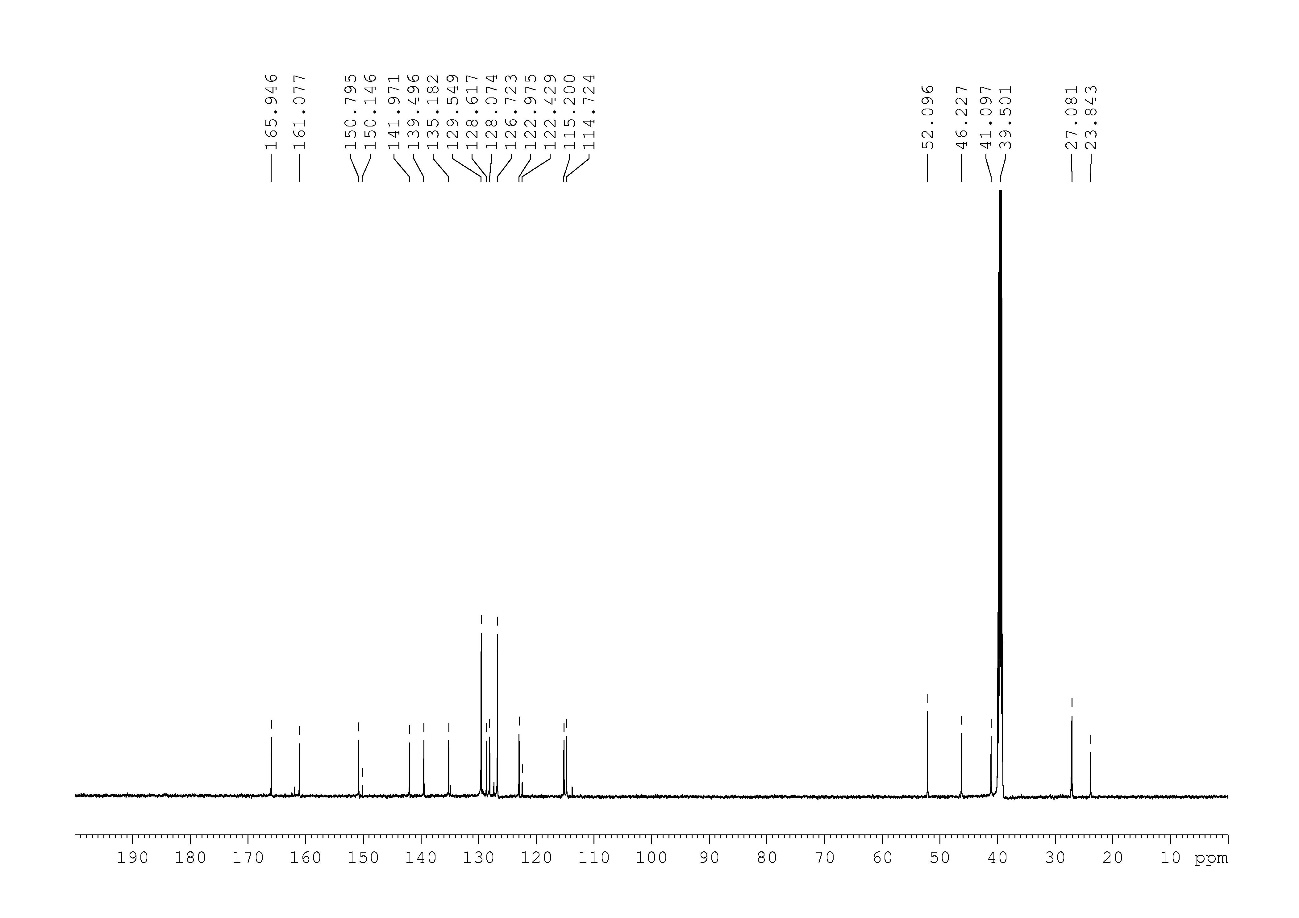


^1^H NMR of **8a** (600 MHz, DMSO-*d*_6_)

^^
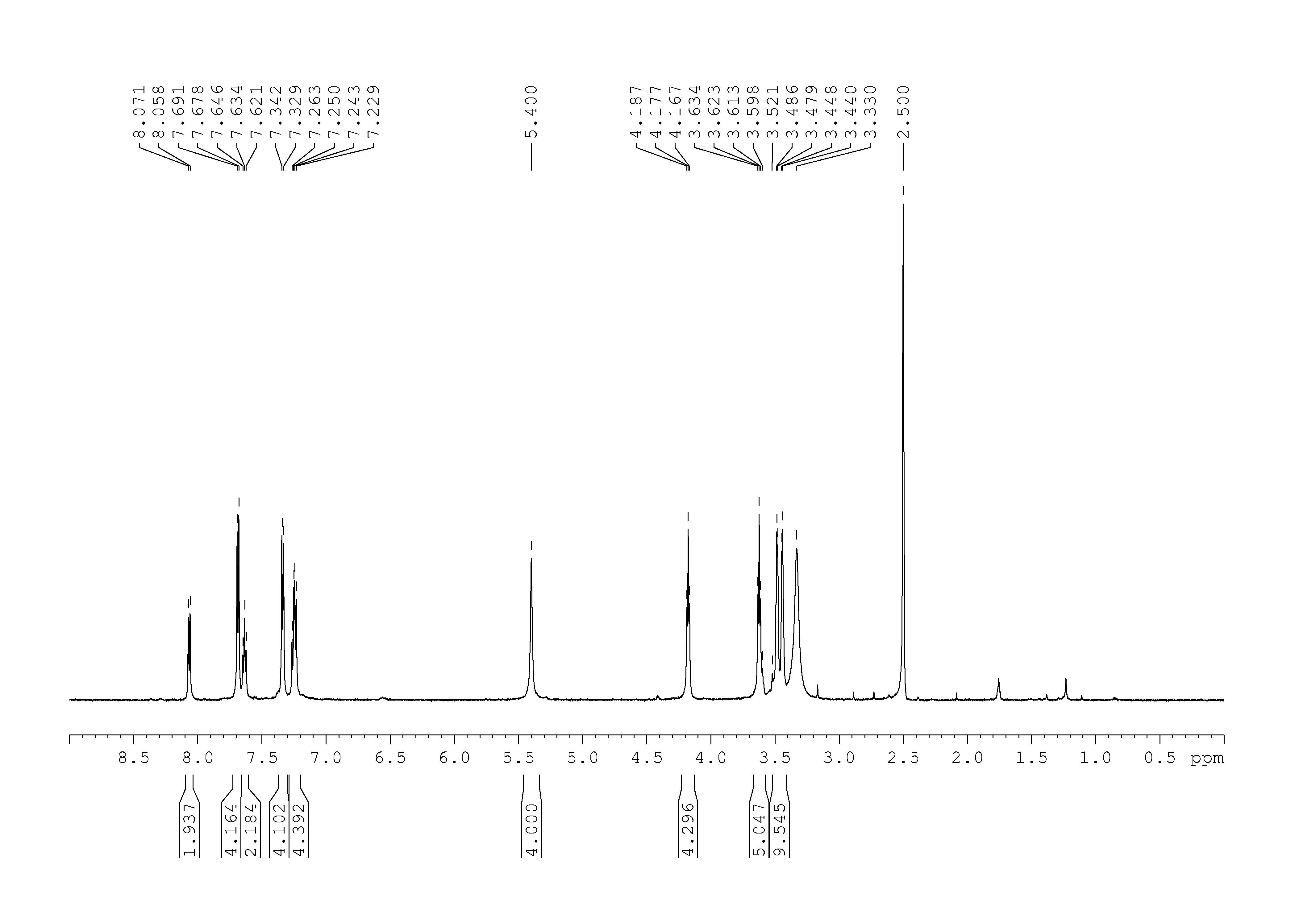


^13^C NMR of **8a** (150 MHz, DMSO-*d*_6_)

^^
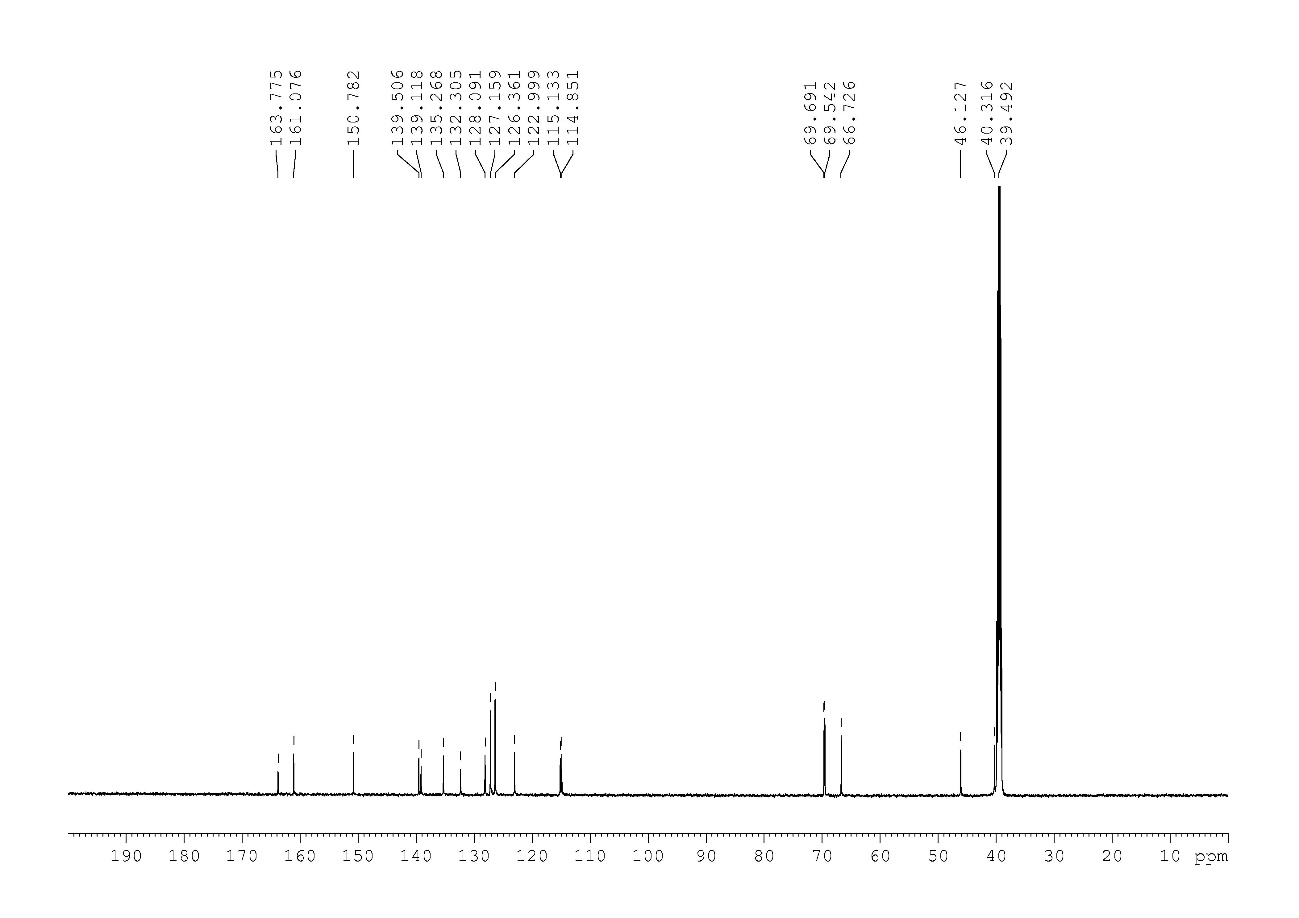


^1^H NMR of **8b** (600 MHz, DMSO-*d*_6_)

^^
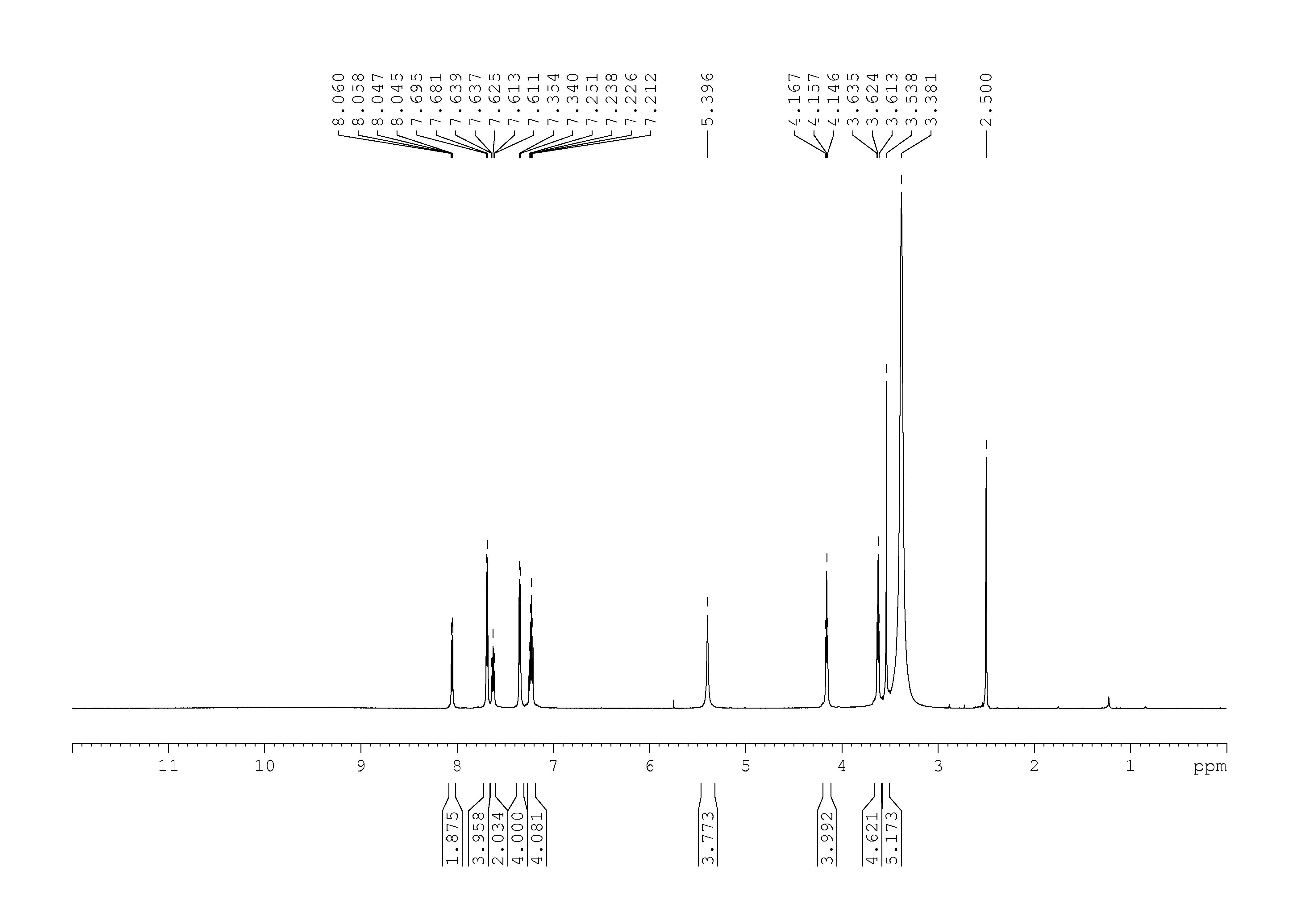


^13^C NMR of **8b** (150 MHz, DMSO-*d*_6_)

^^
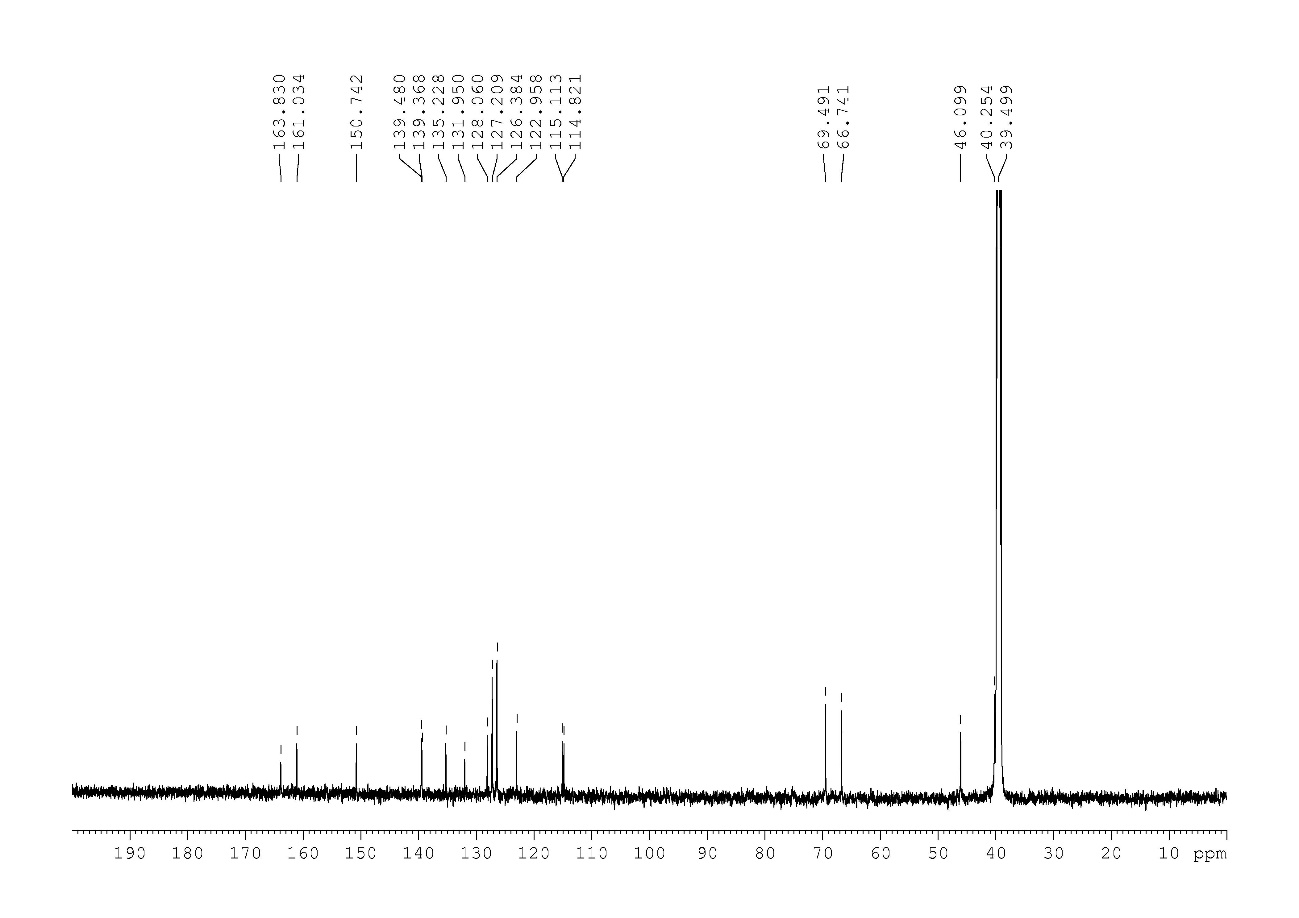


^1^H NMR of **8c** (600 MHz, DMSO-*d*_6_)

^^
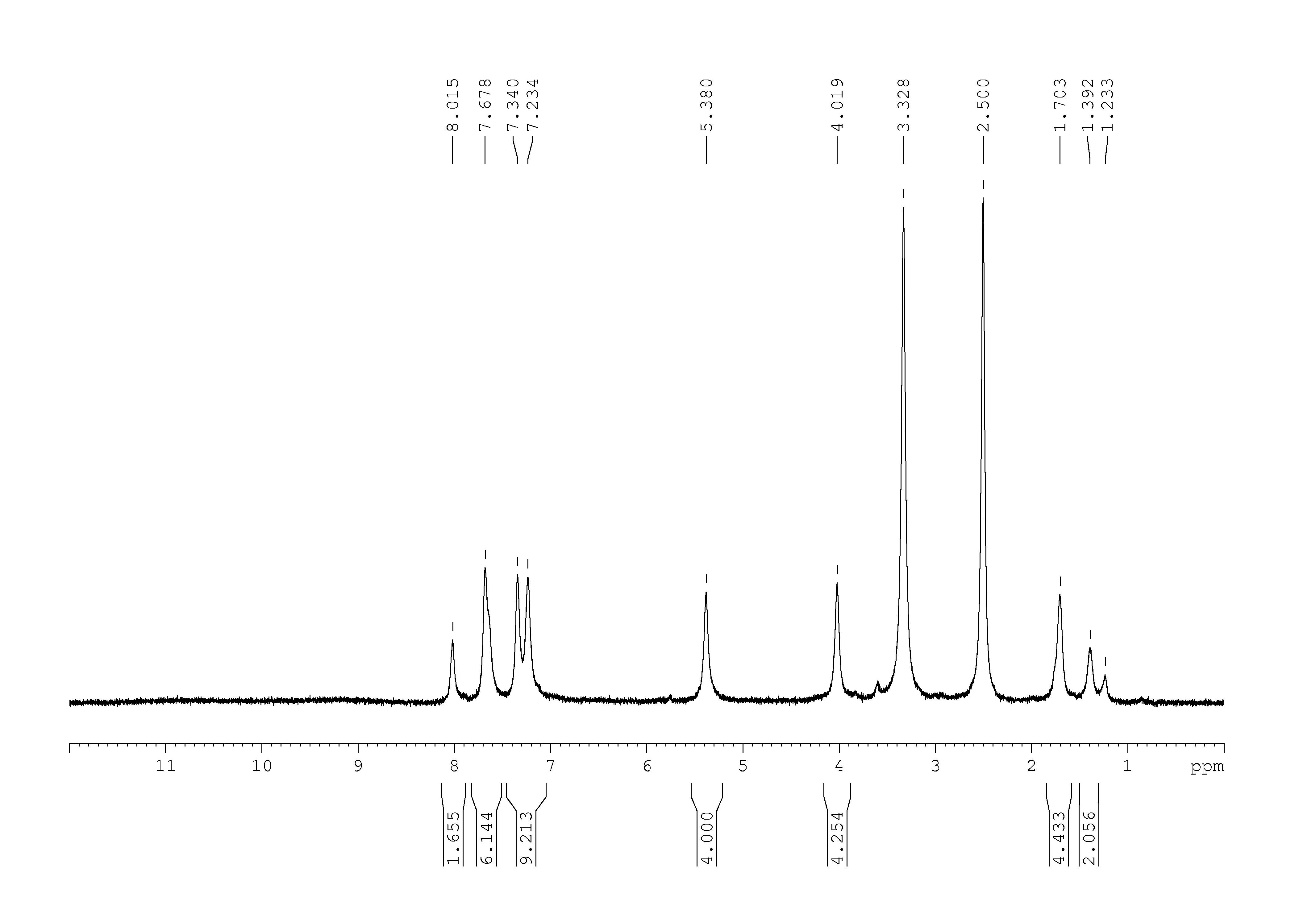


^13^C NMR of **8c** (150 MHz, DMSO-*d*_6_)

^^
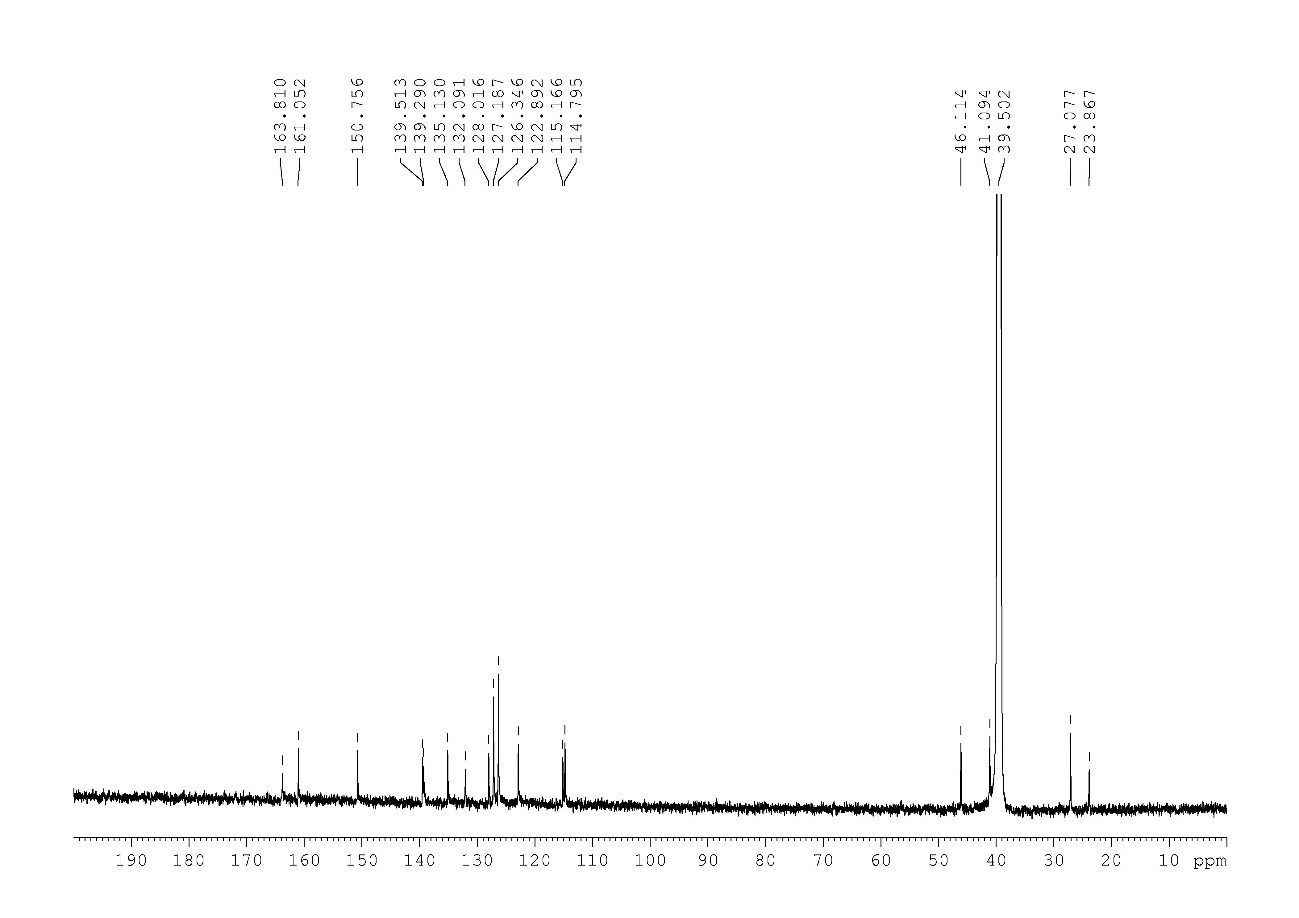


^1^H NMR of **9** (600 MHz, DMSO-*d*_6_)

^^
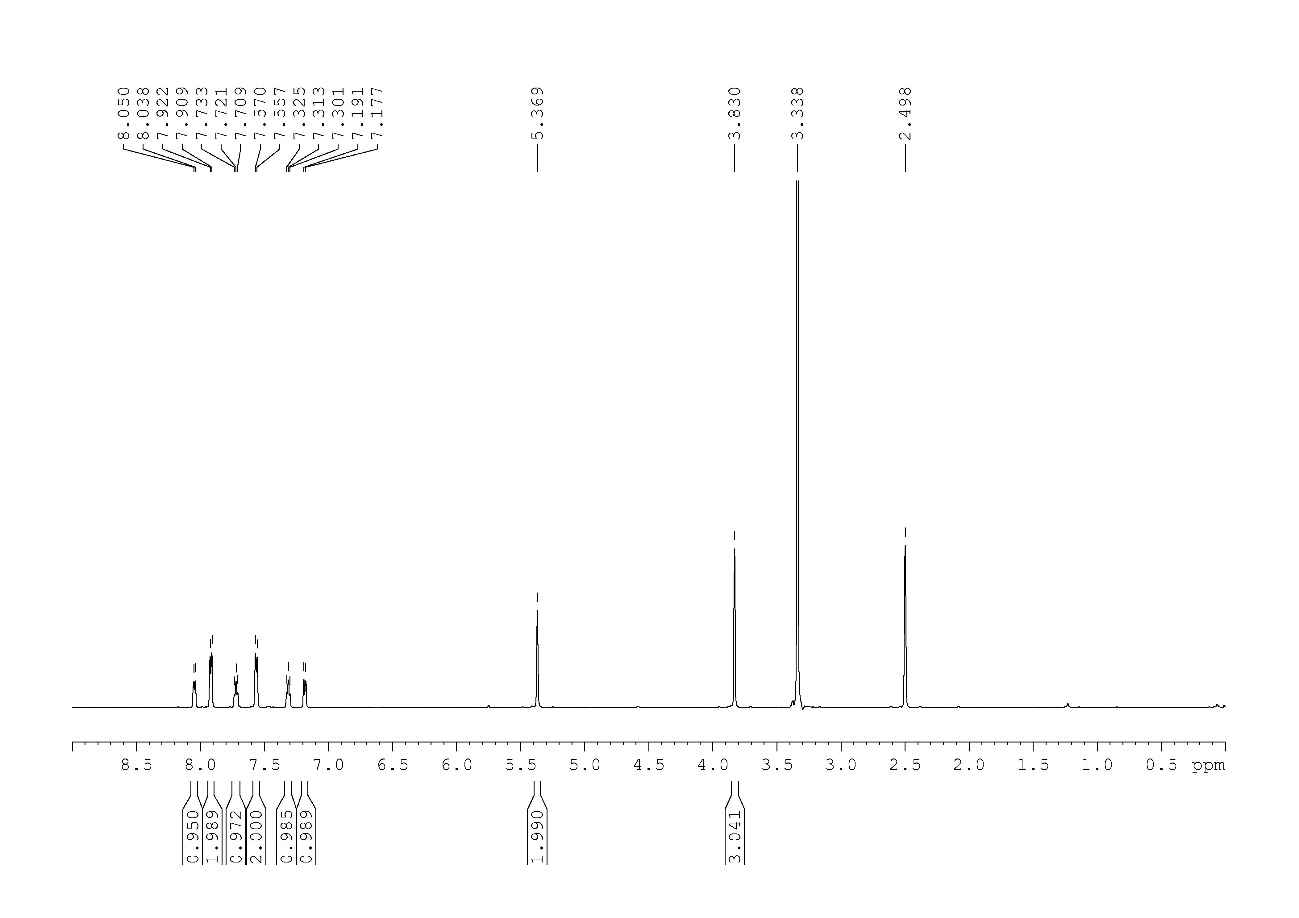


^13^C NMR of **9** (150 MHz, DMSO-*d*_6_)

^^
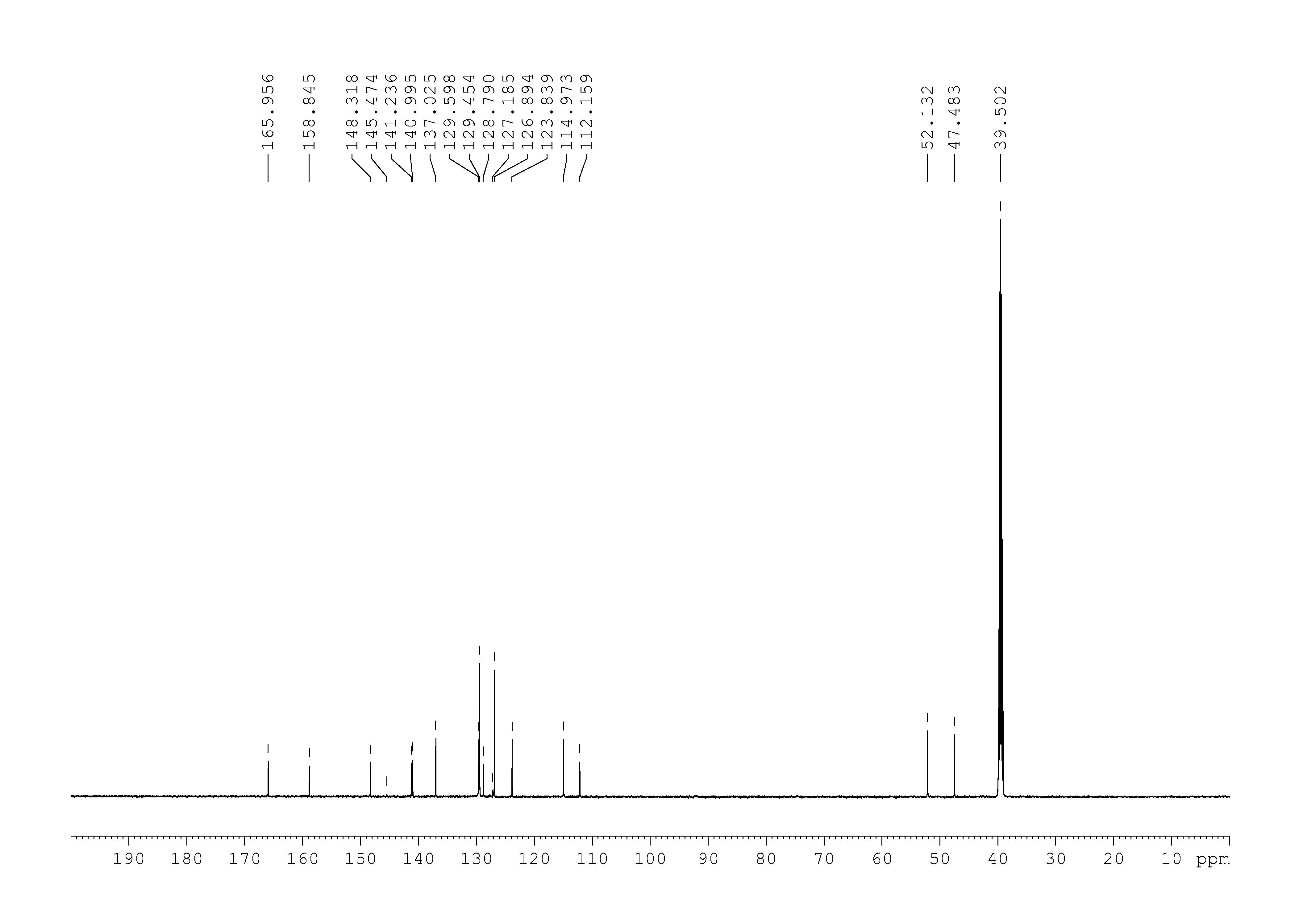


^1^H NMR of **10a** (600 MHz, DMSO-*d*_6_)

^^
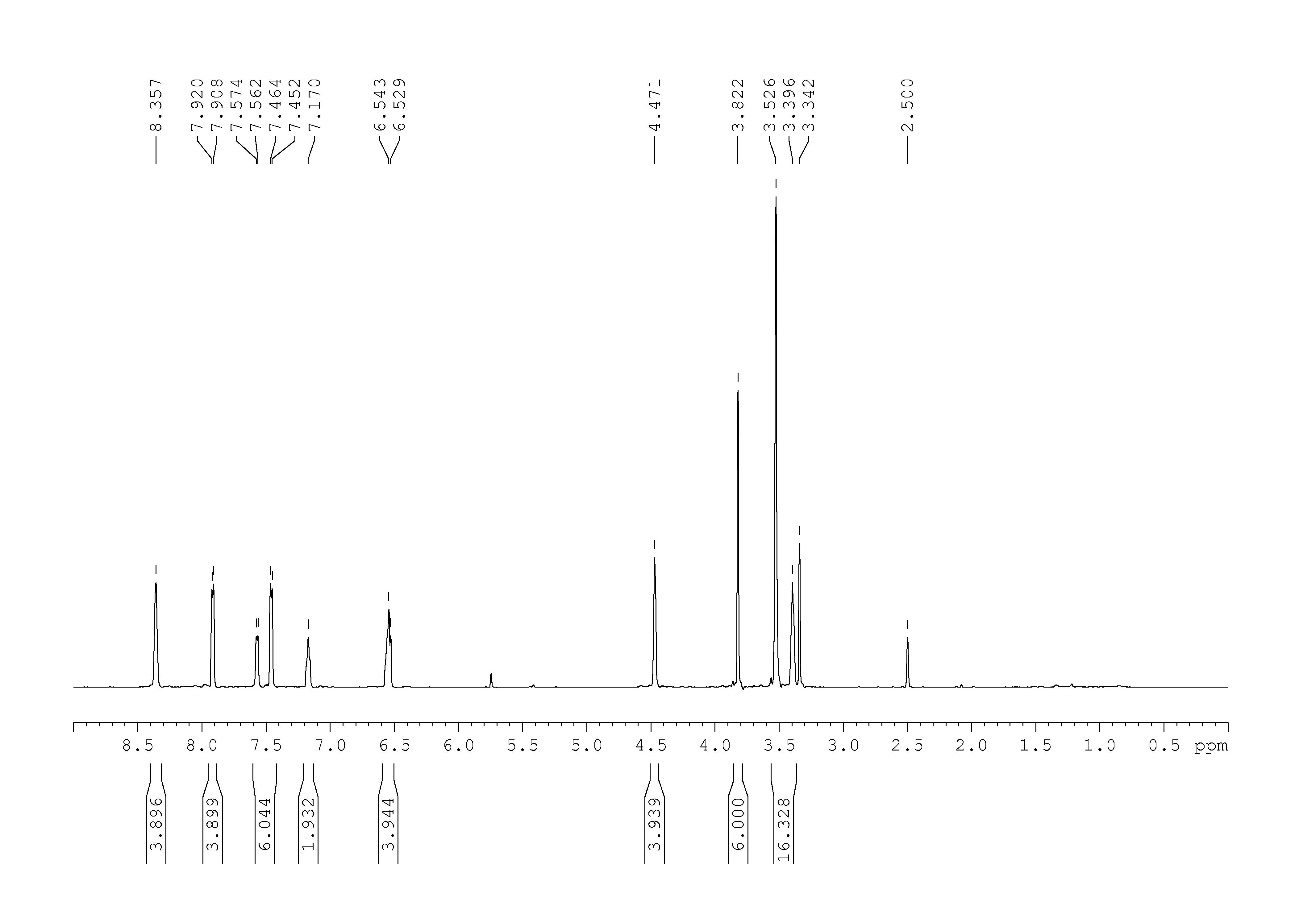


^13^C NMR of **10a** (150 MHz, DMSO-*d*_6_)

^^
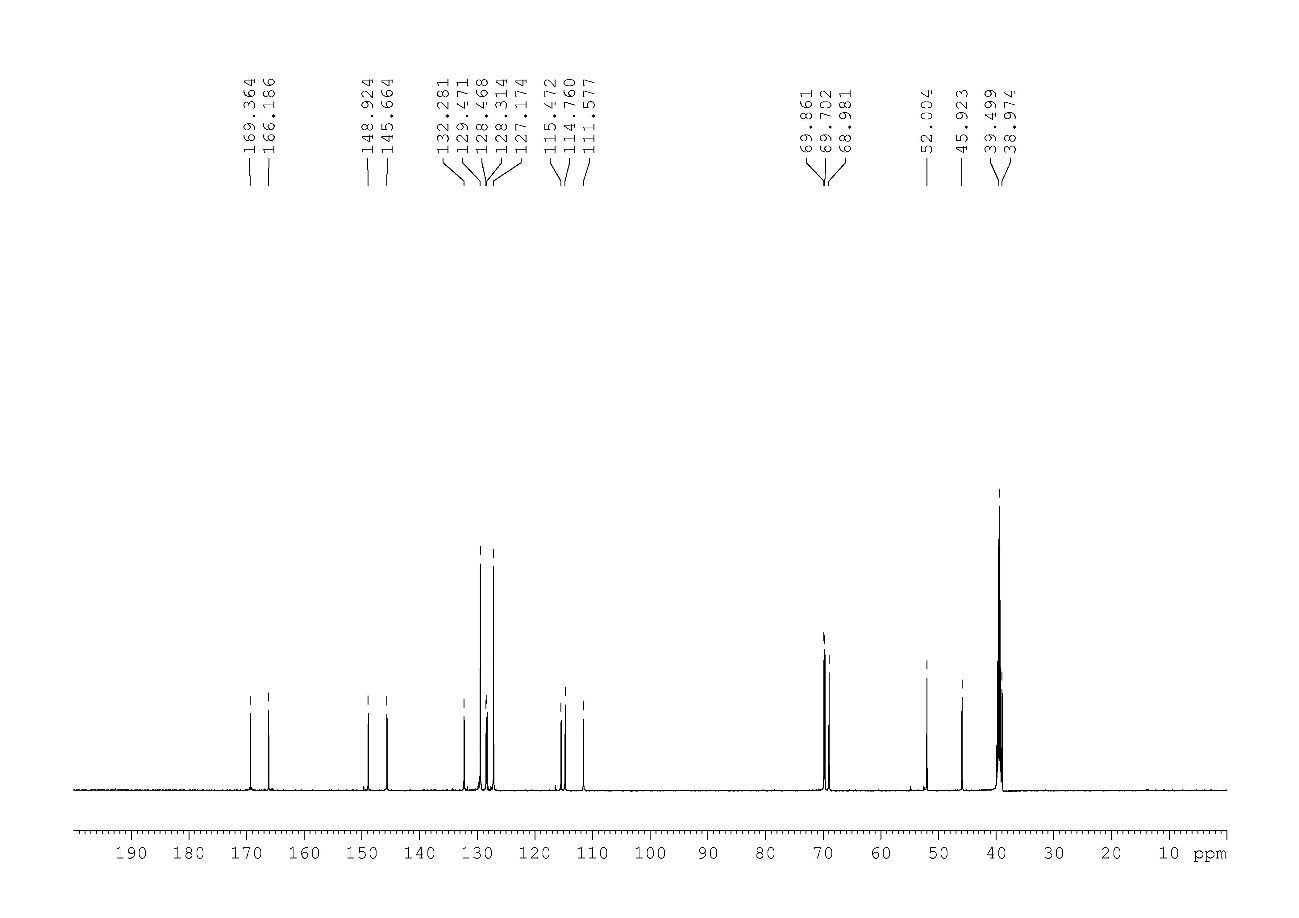


^1^H NMR of **10b** (600 MHz, DMSO-*d*_6_)

^^
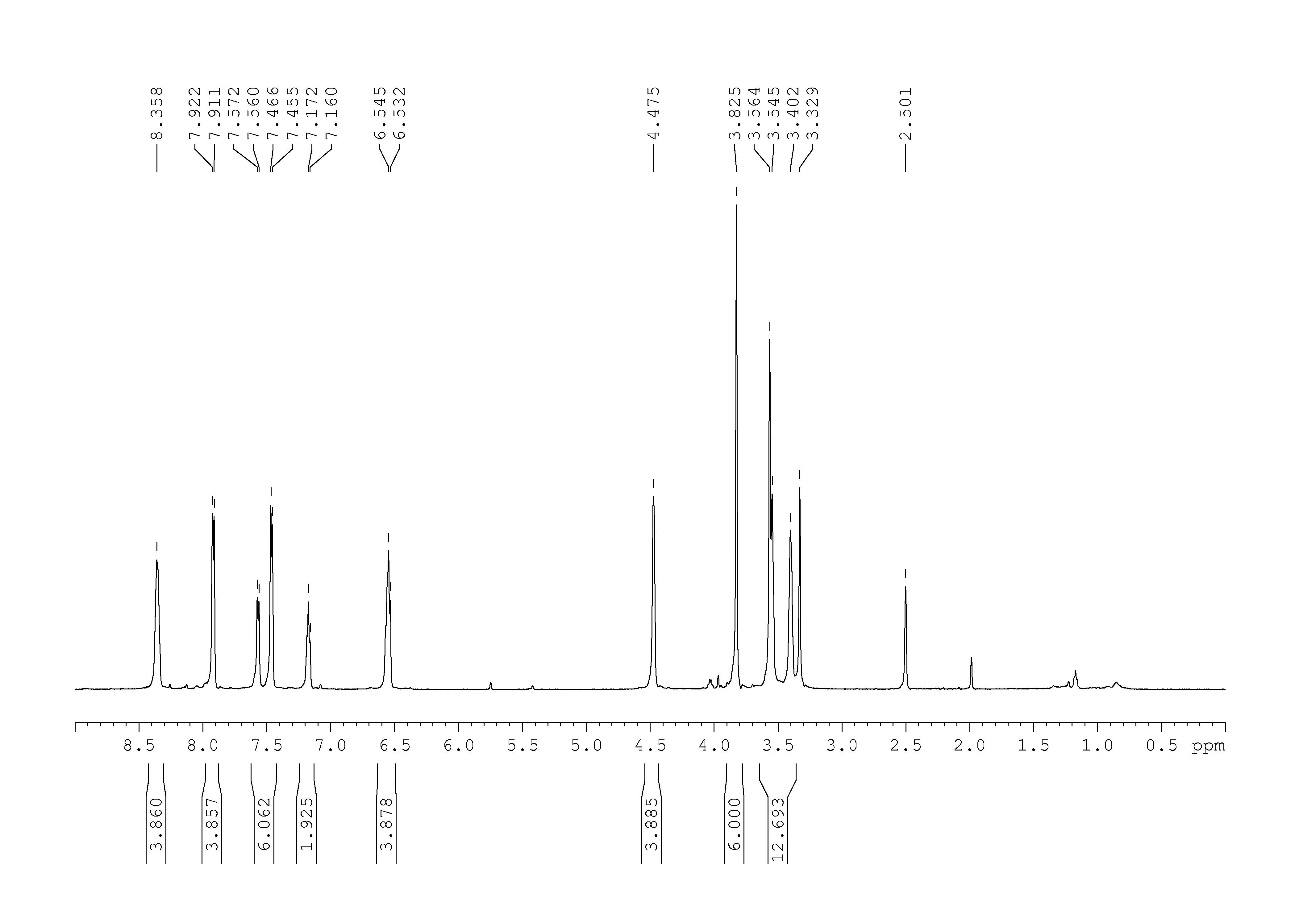


^13^C NMR of **10b** (150 MHz, DMSO-*d*_6_)

^^
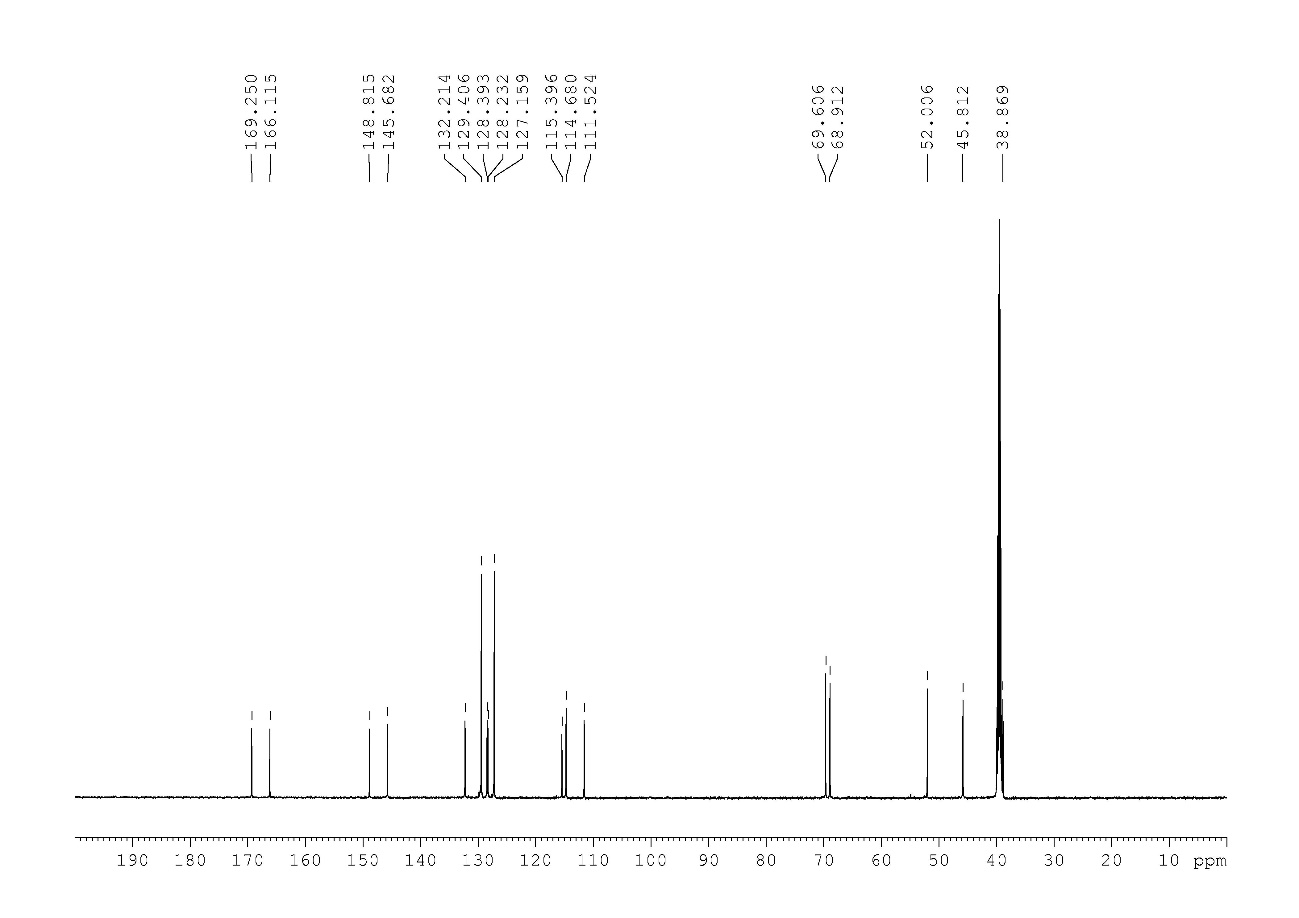


^1^H NMR of **10c** (600 MHz, DMSO-*d*_6_)

^^
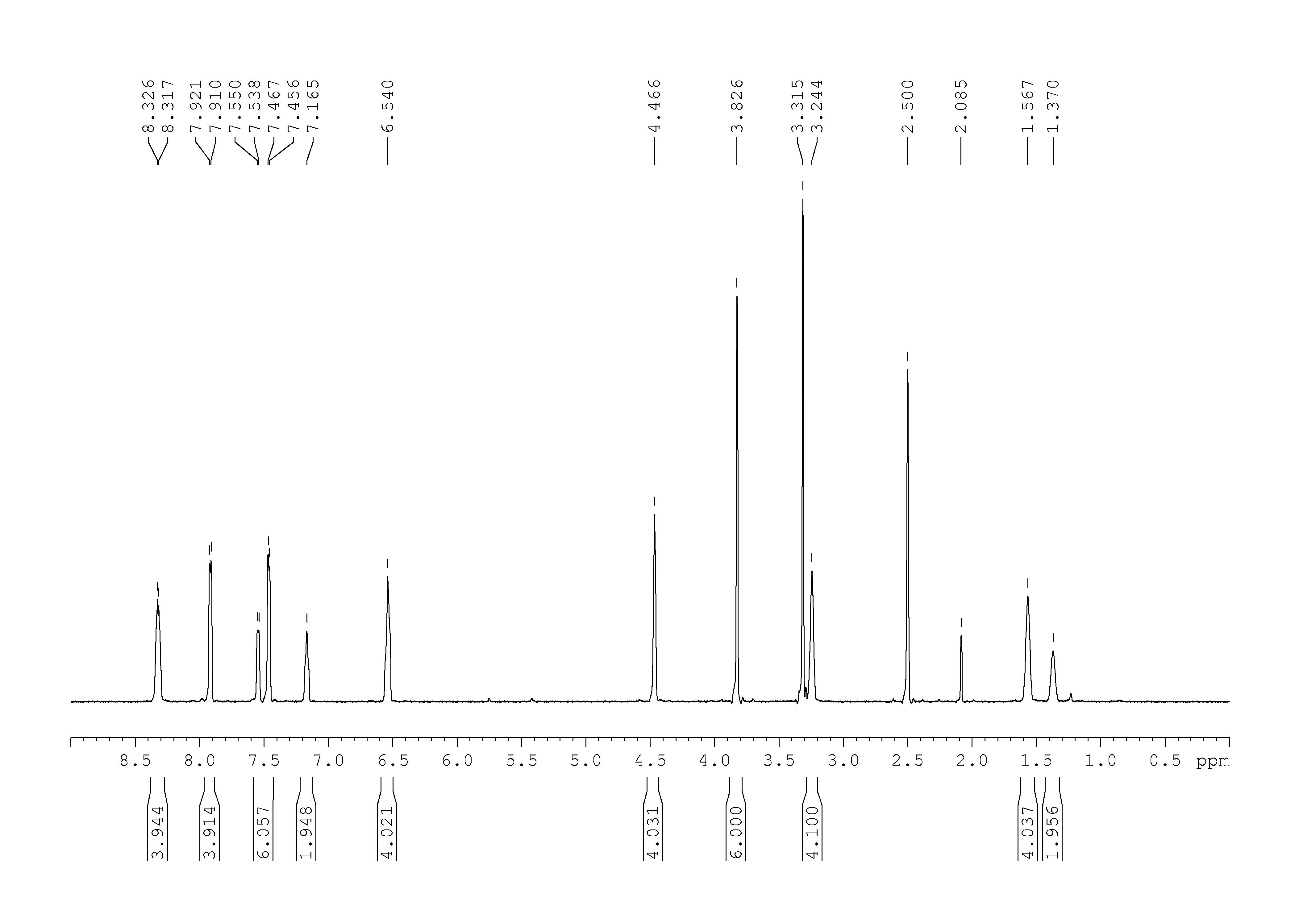


^13^C NMR of **10c** (150 MHz, DMSO-*d*_6_)

^^
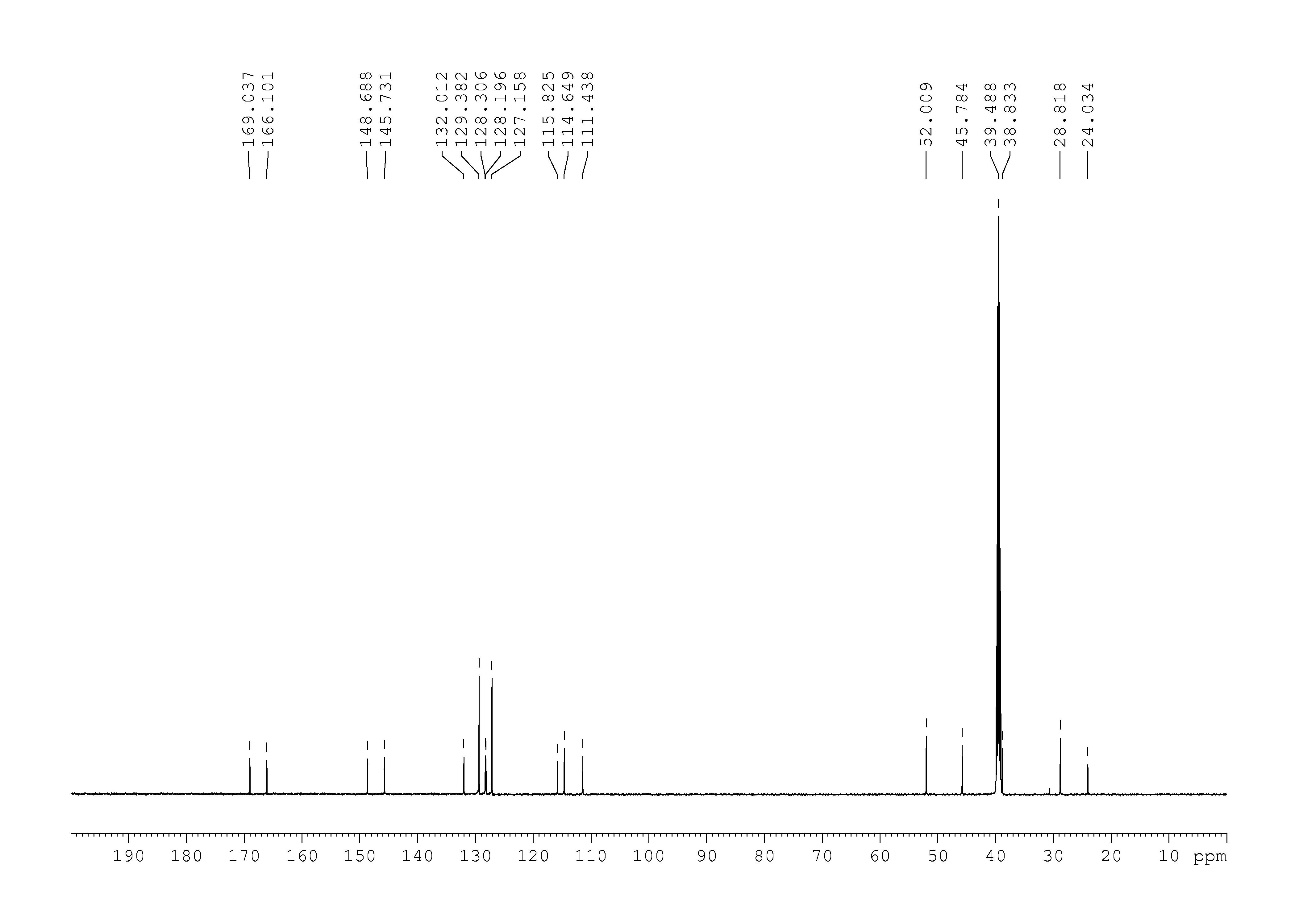


^1^H NMR of **11a** (600 MHz, DMSO-*d*_6_)

^^
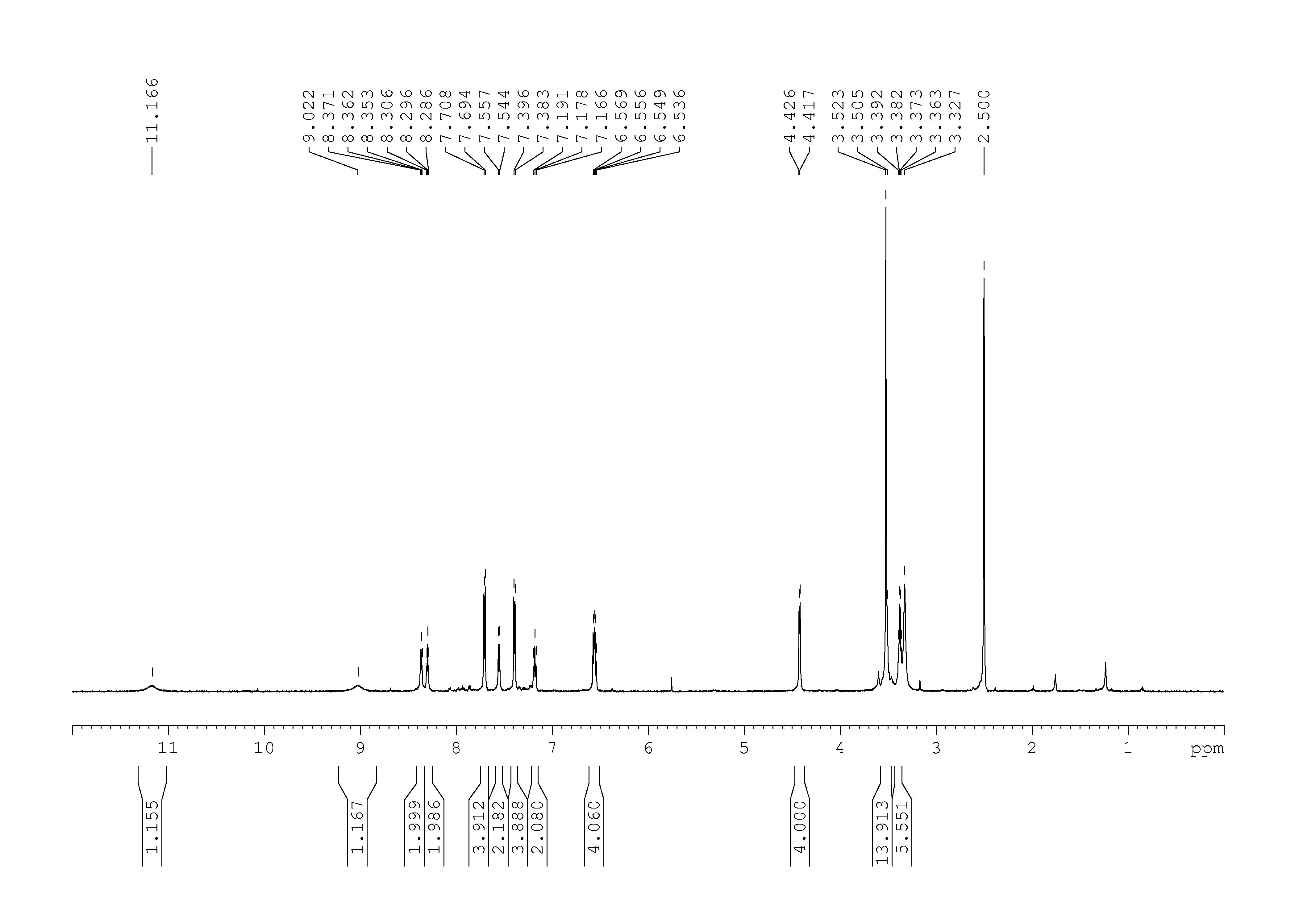


^13^C NMR of **11a** (150 MHz, DMSO-*d*_6_)

^^
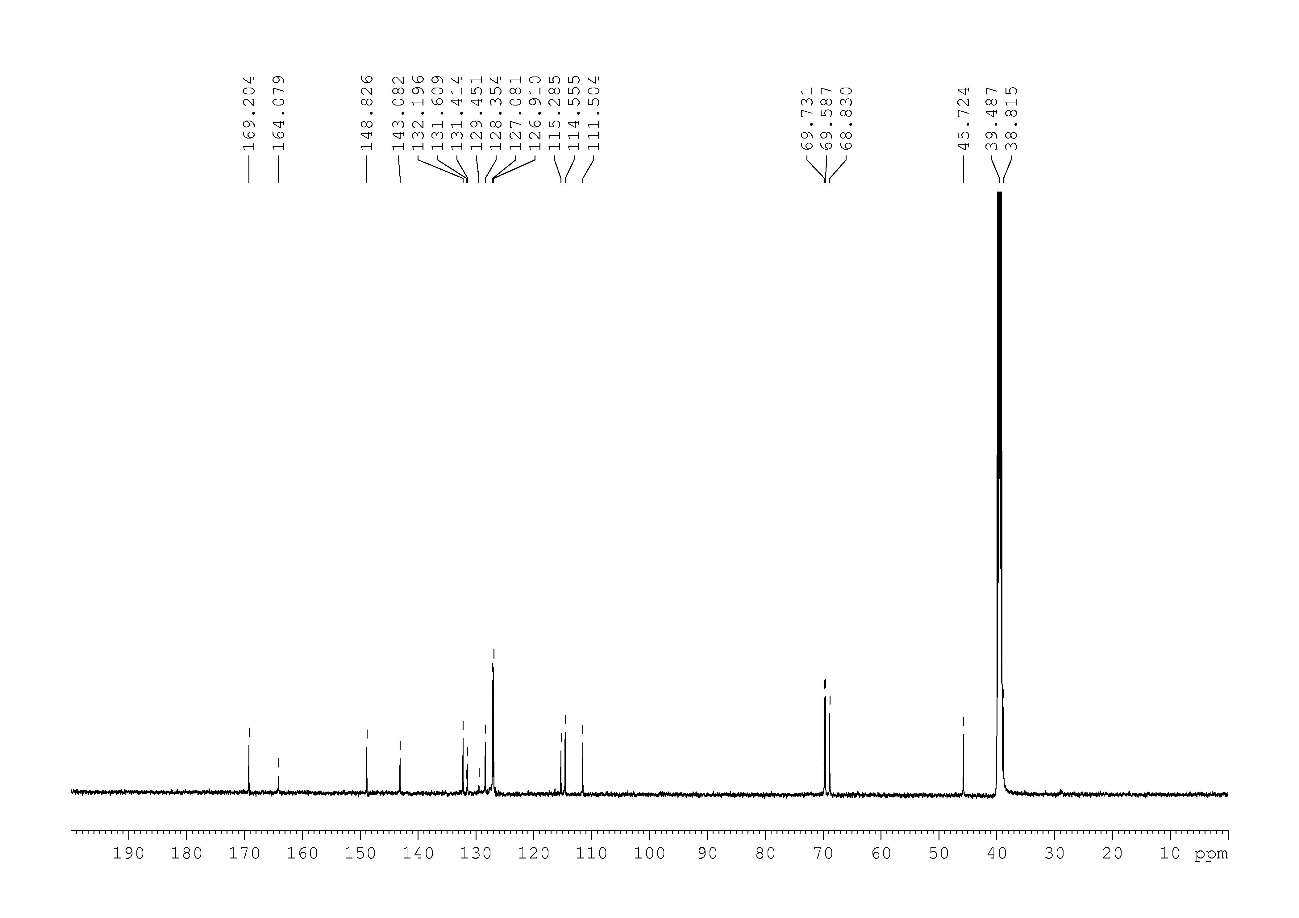


^1^H NMR of **11b** (600 MHz, DMSO-*d*_6_)

^^
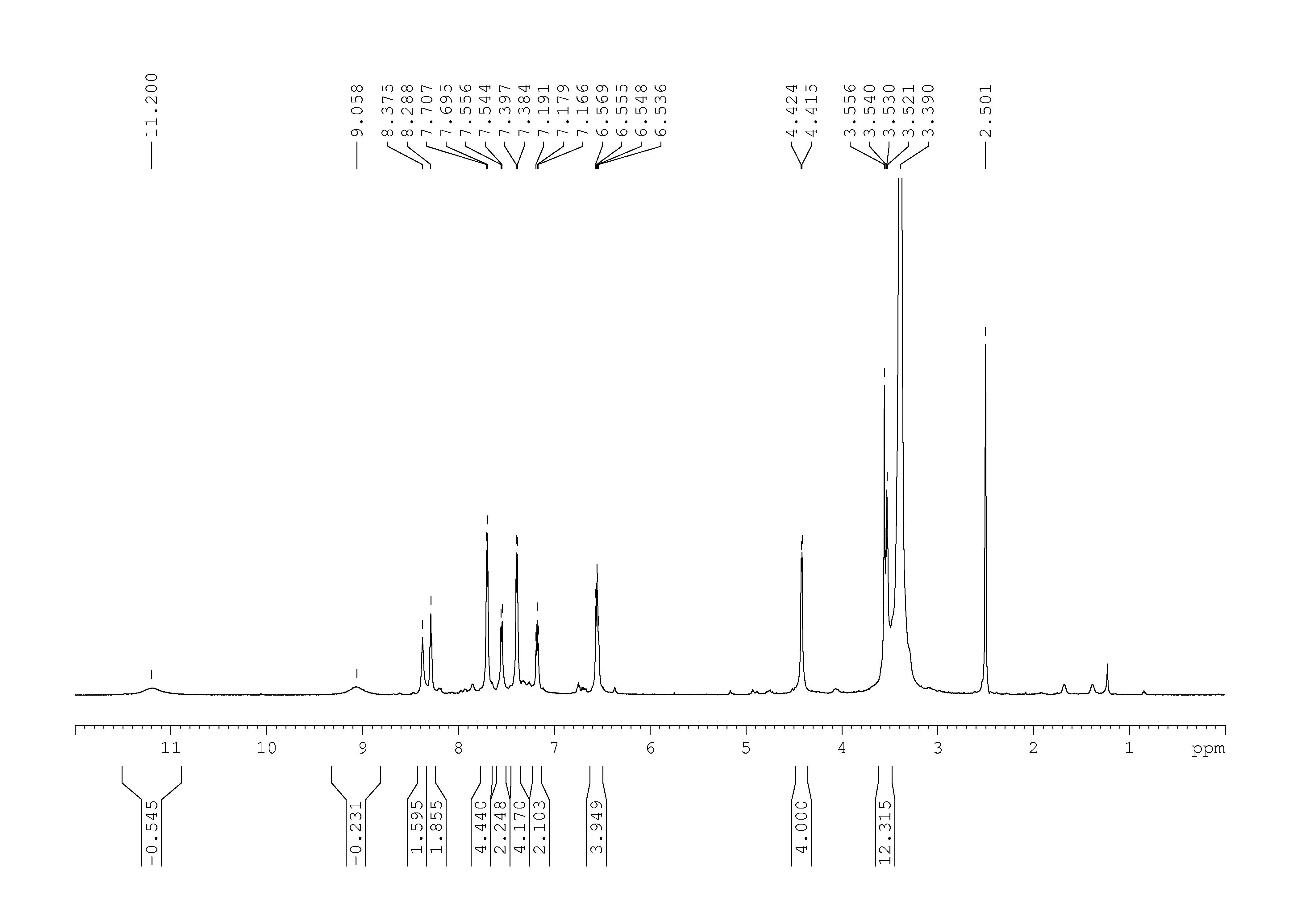


^13^C NMR of **11b** (150 MHz, DMSO-*d*_6_)

^^
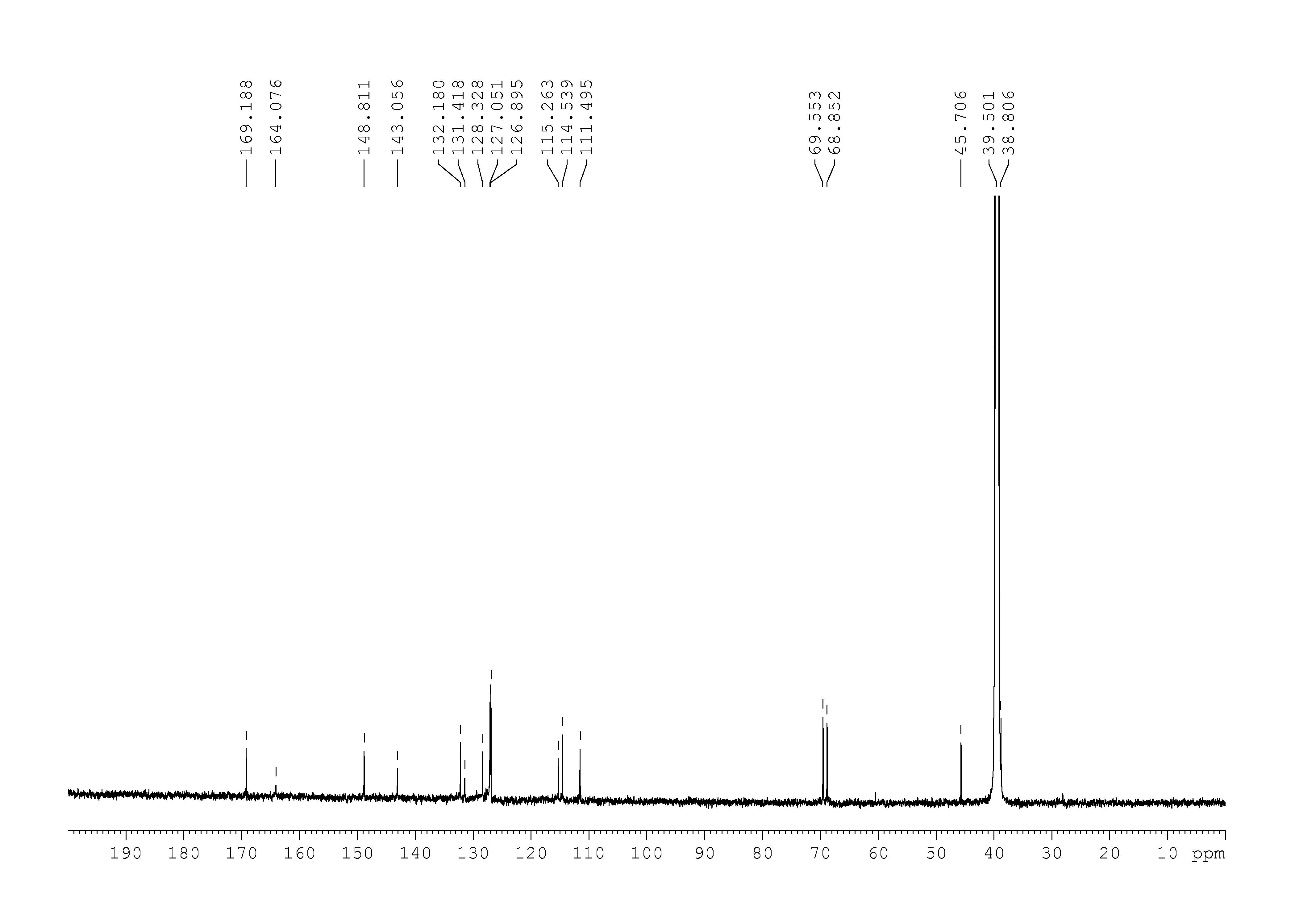


^1^H NMR of **11c** (600 MHz, DMSO-*d*_6_)

^^
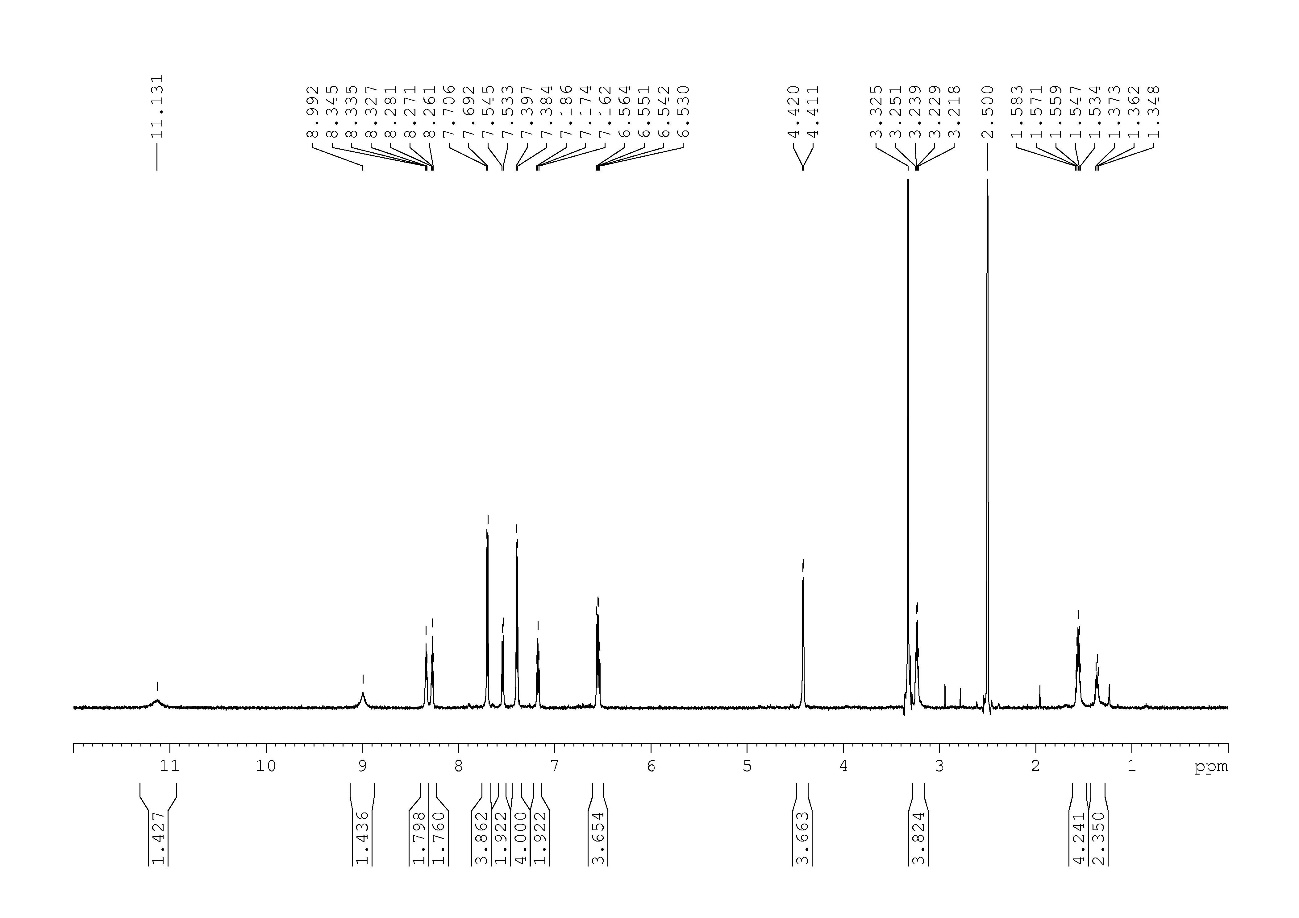


^13^C NMR of **11c** (150 MHz, DMSO-*d*_6_)

^^
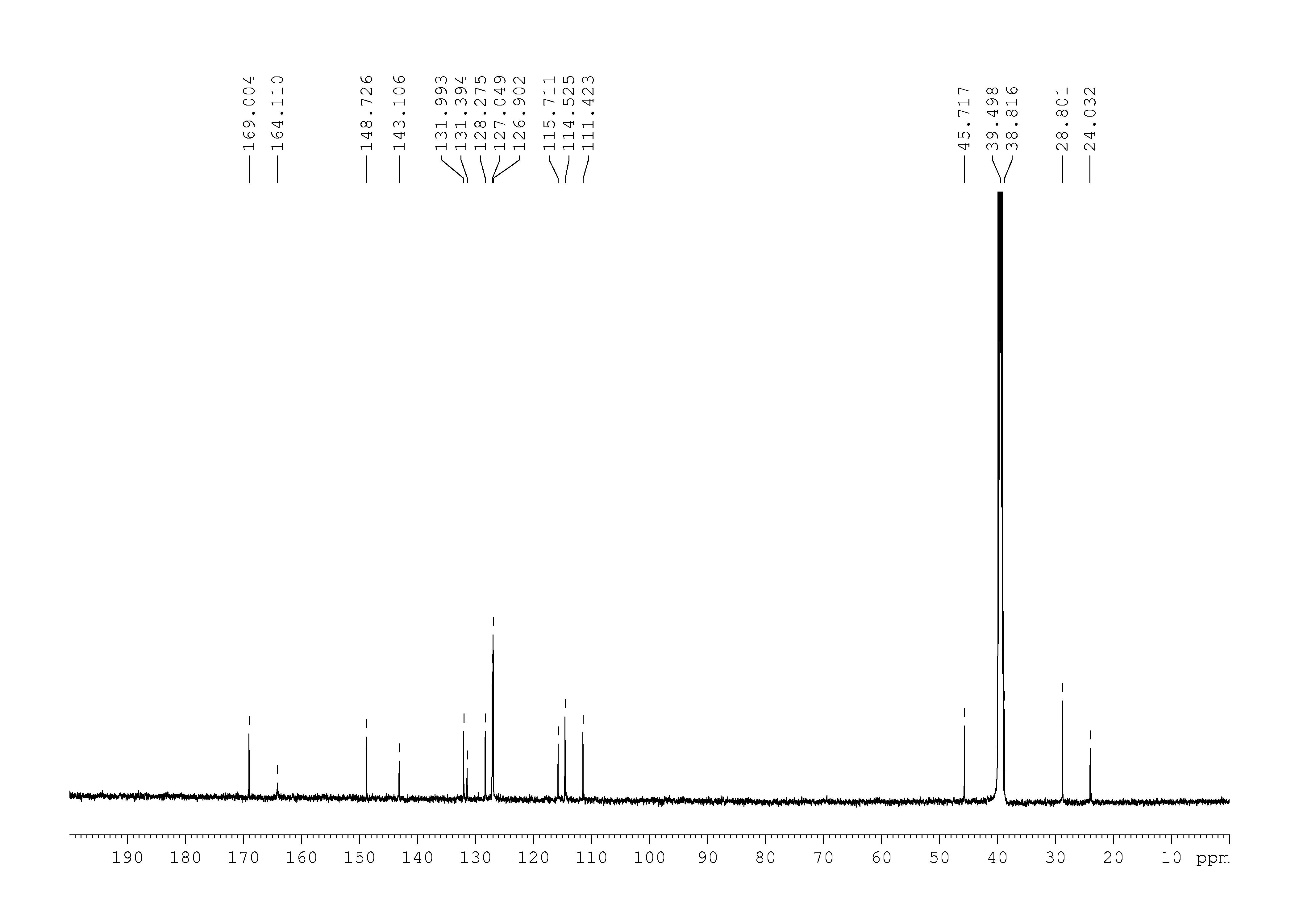


HPLC Spectrum of **4a**

**
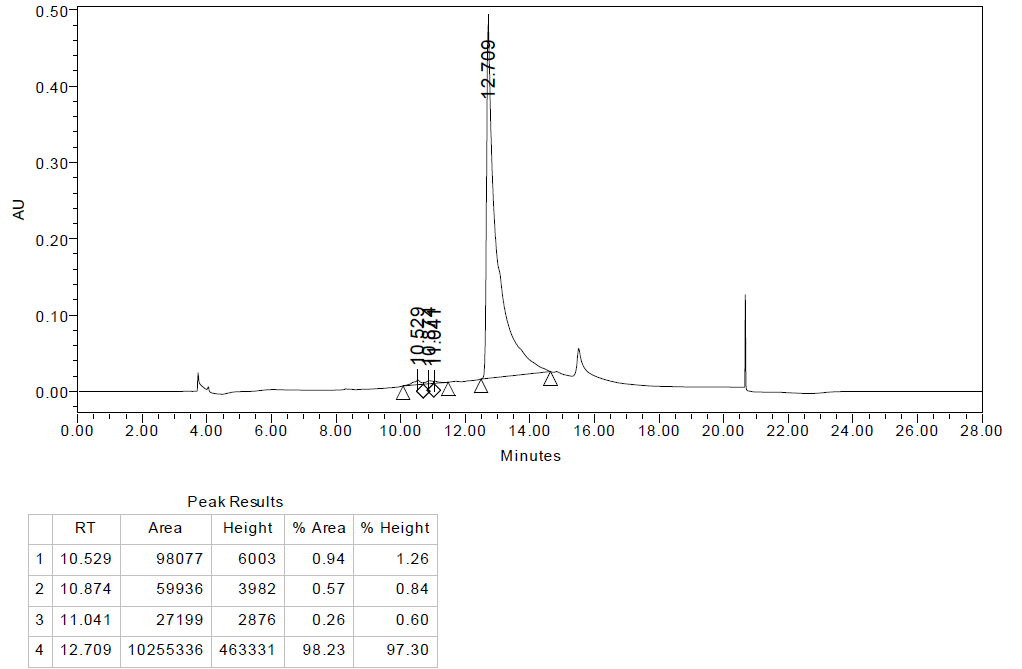
**

HPLC Spectrum of **4b**

**
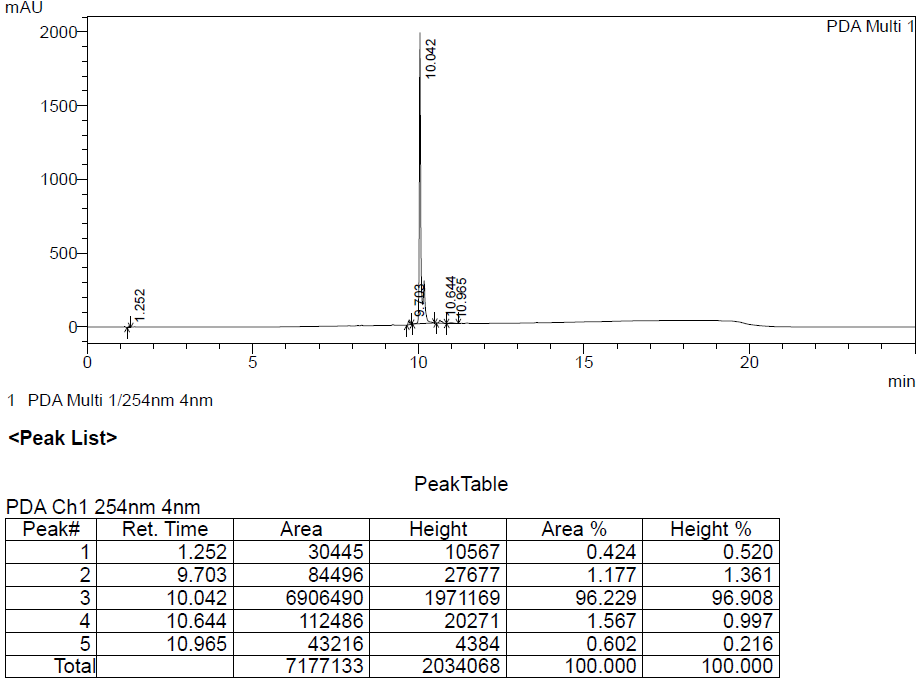
**

HPLC Spectrum of **4c**

**
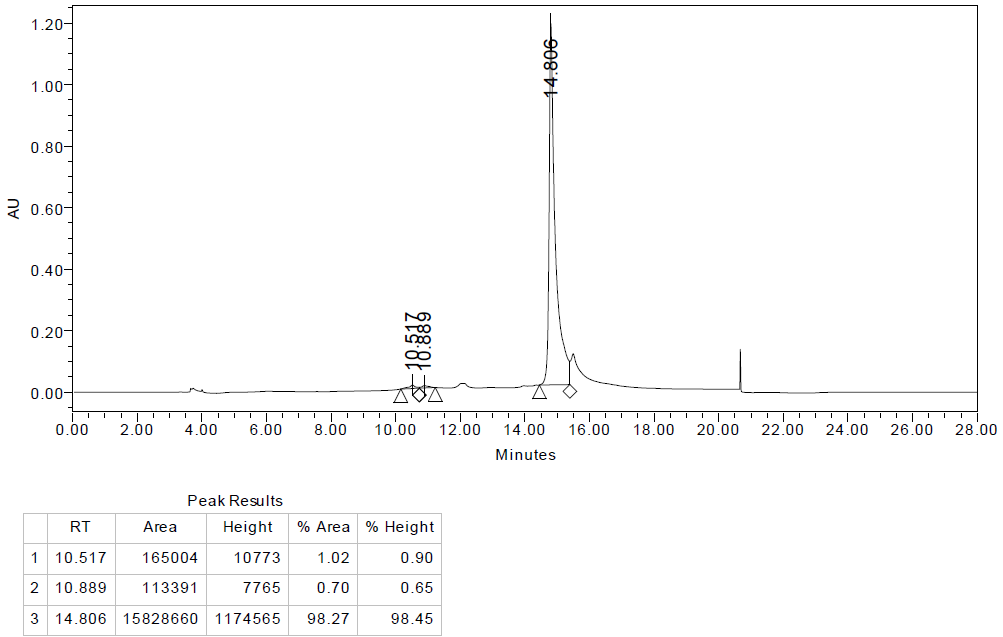
**

HPLC Spectrum of **6a**

**
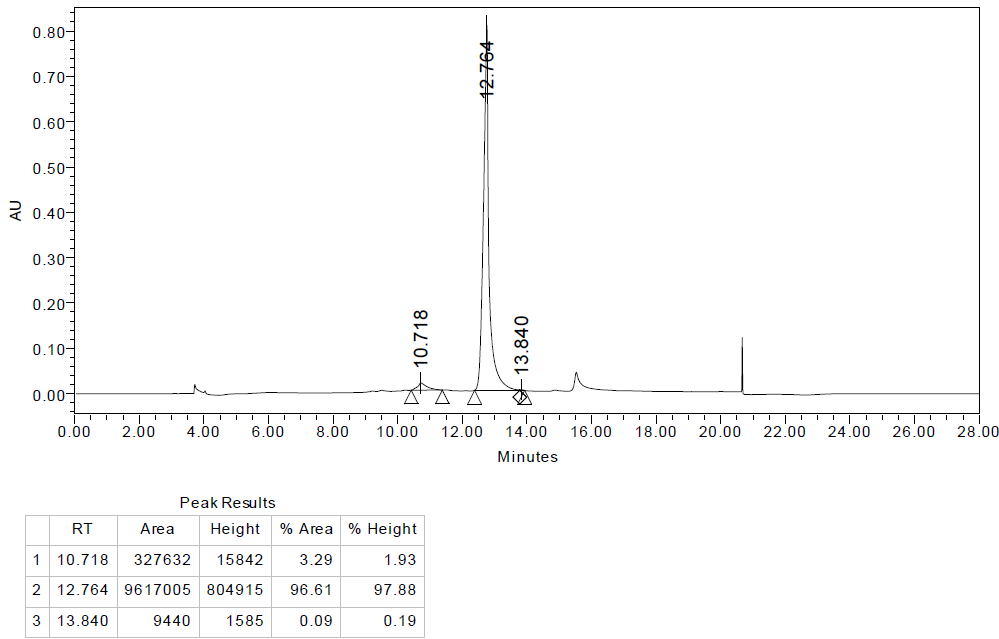
**

HPLC Spectrum of **6b**

**
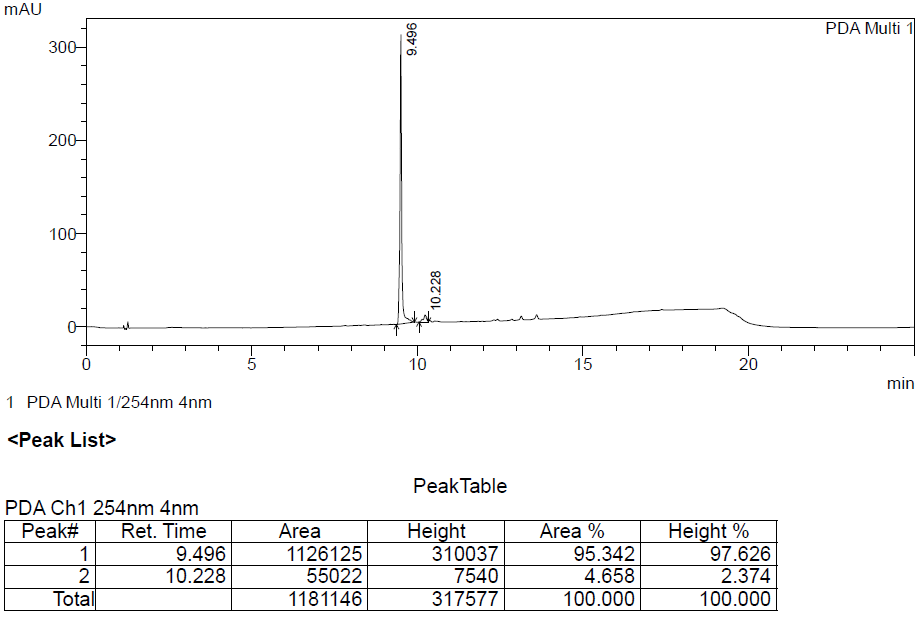
**

HPLC Spectrum of **6c**

**
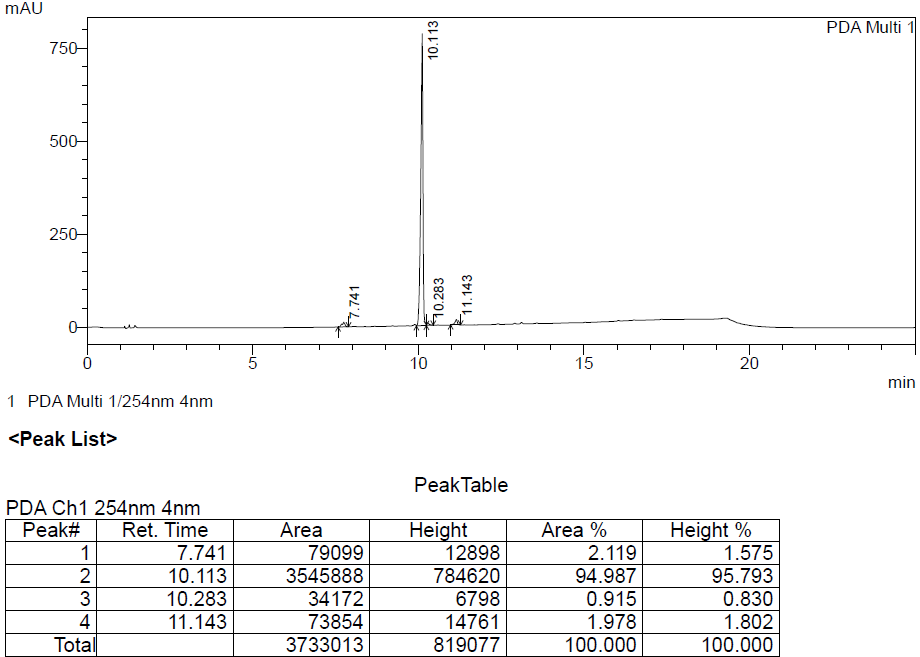
**

HPLC Spectrum of **8a**

**
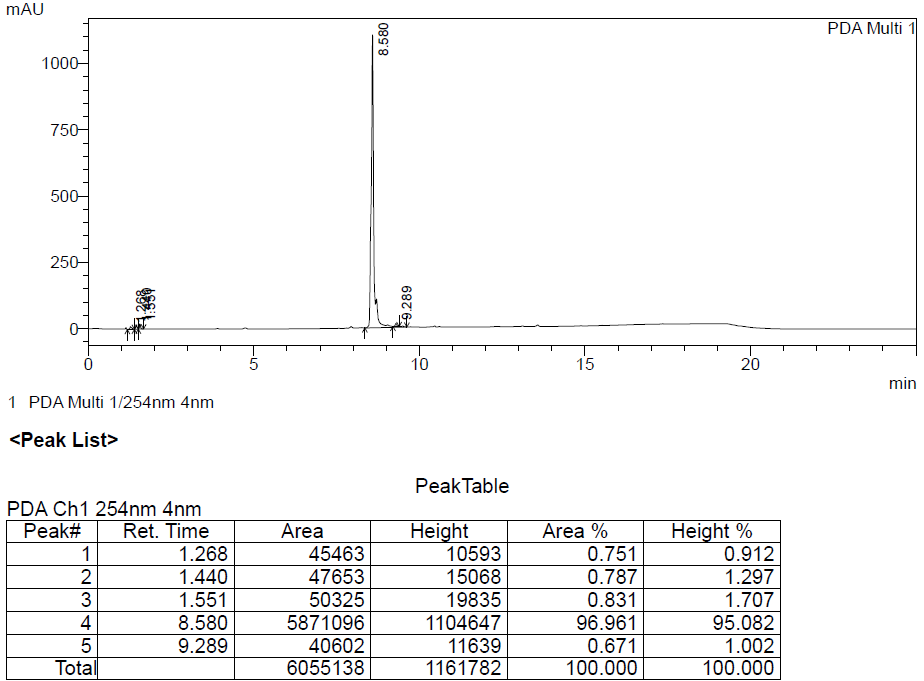
**

HPLC Spectrum of **8b**

**
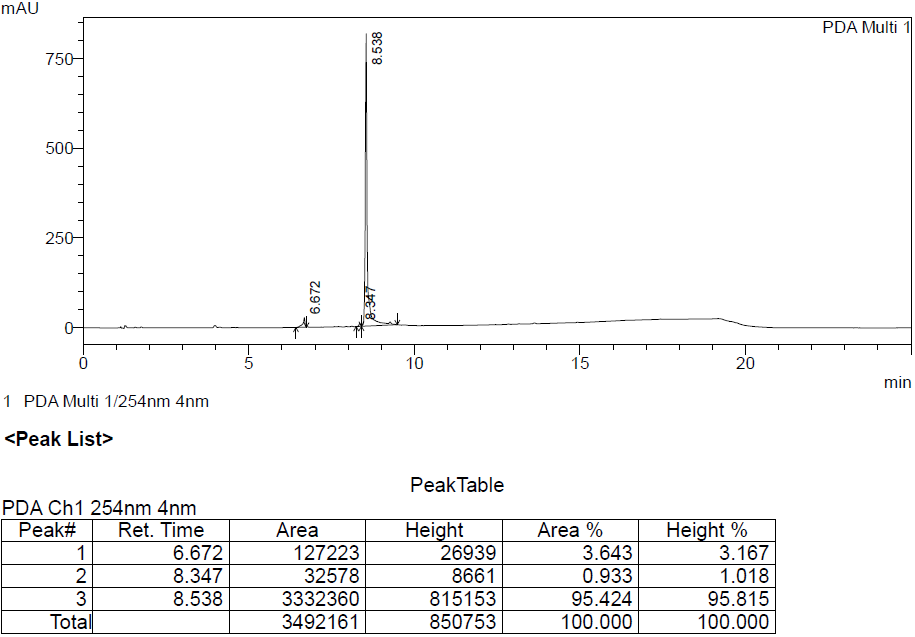
**

HPLC Spectrum of **8c**

**
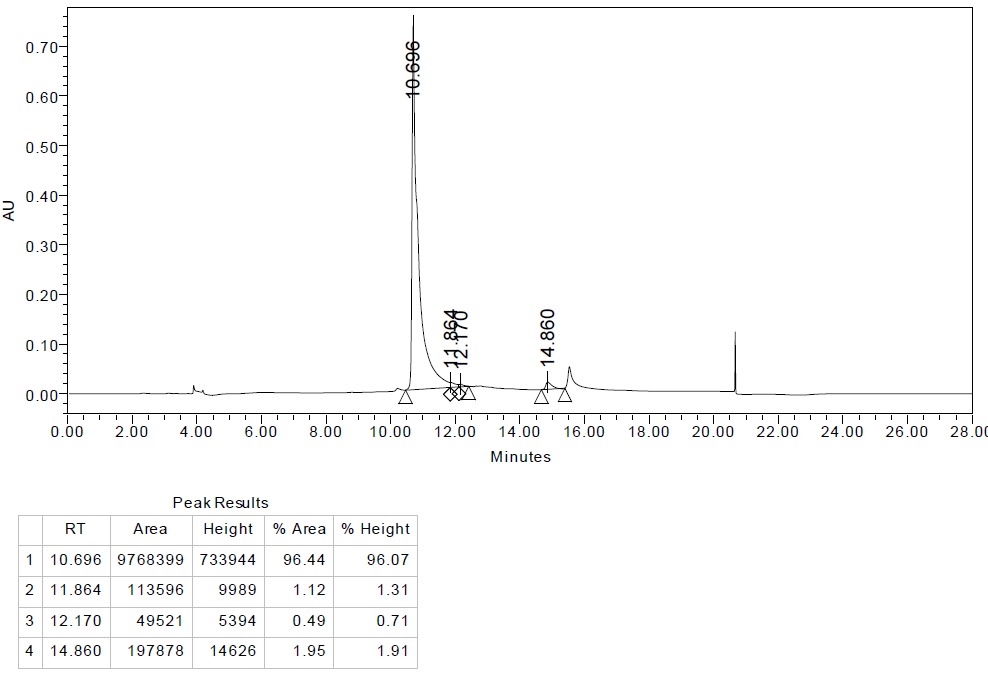
**

HPLC Spectrum of **11a**

**
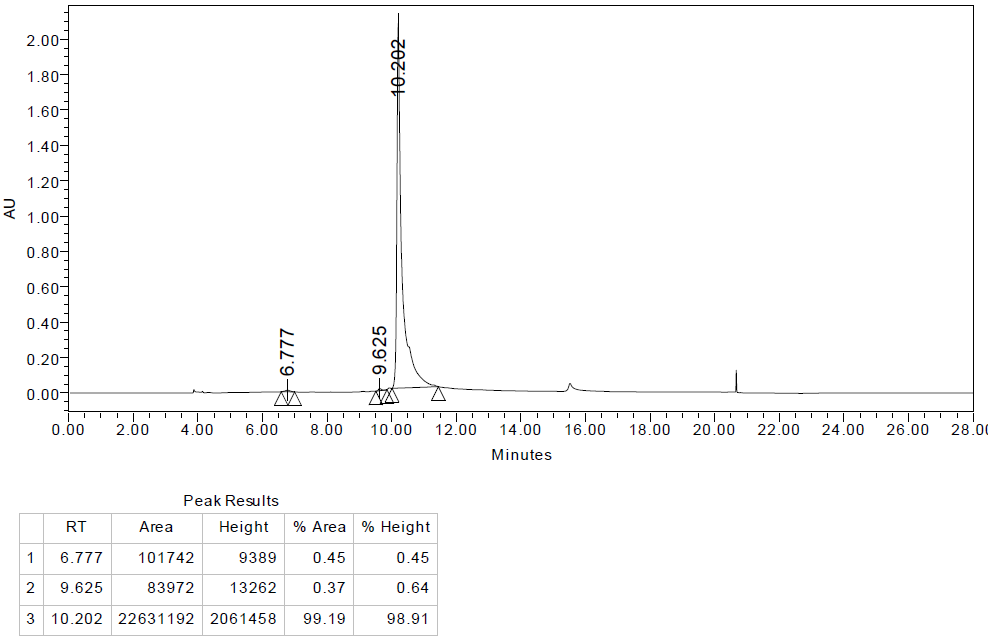
**

HPLC Spectrum of **11b**

**
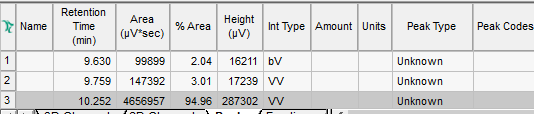

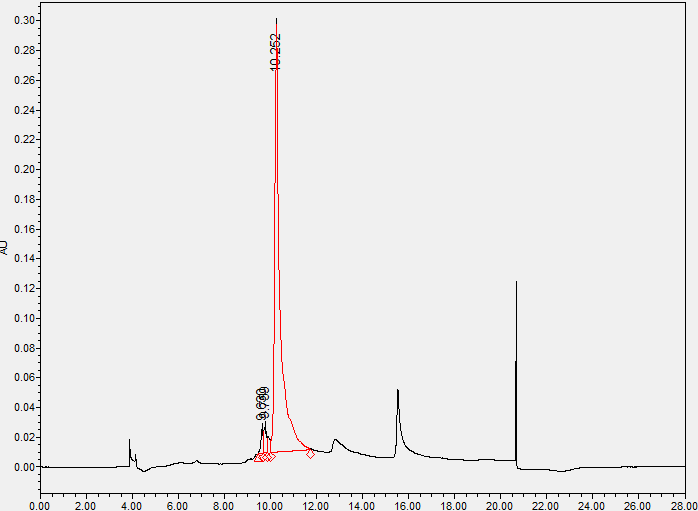
**

HPLC Spectrum of **11c**

**
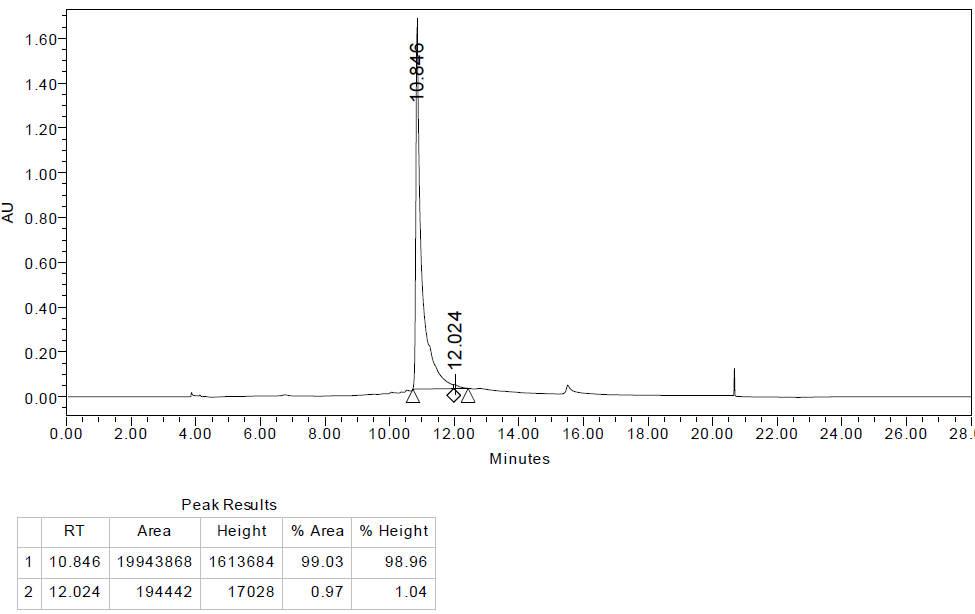
**
